# Identification of hyper-rewired genomic stress non-oncogene addiction genes across 15 cancer types

**DOI:** 10.1038/s41540-019-0104-5

**Published:** 2019-08-07

**Authors:** Jessica Xin Hjaltelin, Jose M. G. Izarzugaza, Lars Juhl Jensen, Francesco Russo, David Westergaard, Søren Brunak

**Affiliations:** 1 Novo Nordisk Foundation Center for Protein Research, University of Copenhagen, DK-2200 Copenhagen, Denmark; 2 Department of Bio and Health Informatics, Technical University of Denmark, DK-2800 Kgs. Lyngby, Denmark; 3 Copenhagen University Hospital, Rigshospitalet, Blegdamsvej 9, DK-2100 Copenhagen, Denmark

**Keywords:** Computational biology and bioinformatics, Cancer

## Abstract

Non-oncogene addiction (NOA) genes are essential for supporting the stress-burdened phenotype of tumours and thus vital for their survival. Although NOA genes are acknowledged to be potential drug targets, there has been no large-scale attempt to identify and characterise them as a group across cancer types. Here we provide the first method for the identification of conditional NOA genes and their rewired neighbours using a systems approach. Using copy number data and expression profiles from The Cancer Genome Atlas (TCGA) we performed comparative analyses between high and low genomic stress tumours for 15 cancer types. We identified 101 condition-specific differential coexpression modules, mapped to a high-confidence human interactome, comprising 133 candidate NOA rewiring hub genes. We observe that most modules lose coexpression in the high-stress state and that activated stress modules and hubs take part in homoeostasis maintenance processes such as chromosome segregation, oxireductase activity, mitotic checkpoint (PLK1 signalling), DNA replication initiation and synaptic signalling. We furthermore show that candidate NOA rewiring hubs are unique for each cancer type, but that their respective rewired neighbour genes largely are shared across cancer types.

## Using the computer to find the Achilles heel of cancer

Tumour cells are under many types of stresses from their environments and they activate certain genes to stay alive. These ‘Achilles heel genes’ could be potential new drug targets for cancer. A team led by Søren Brunak at Copenhagen University in Denmark used a catalogue of cancer data available online to identify these Achilles heel genes using network biology. They found several networks across 15 cancer types that are significantly more active in the stressed state. This study is the first to use systems level approaches to identify stress genes in 15 cancer types. A key result is that the stress-activated genes themselves are not shared across cancer types, but their rewired neighbours are. This underscores the idea of an overall stress-specific mechanism which is not necessarily cancer type-specific. Condition-specific genes will have mounting impact on cancer research and personalised cancer therapeutics.

## Introduction

Non-oncogene addiction (NOA) is the phenomenon where tumour cells present stress phenotypes that instigate a dependency on support genes for survival.^1–3^ This phenomenon was first characterised in Solimini et al.^1^ More specifically, NOA genes have been defined as non-mutated genes that are upregulated to uphold the stress phenotypes of cancer cells,^1,4^ making them stress-specific. Tumour cells are under various types of stresses and possess fewer regulatory mechanisms to compensate for perturbations (i.e. buffering capacity) compared with their normal counterparts.^3^ The activation of NOA genes is unnecessary for normal cells but essential for cancer cells that are burdened by the stress caused by for example oncogenes. A new generation of treatments are starting to exploit these Achilles’ heels, owing to experimental and clinical evidence showing that they have a much wider space that can be mined for drug targets compared with the classical oncogenes.^5,6^

Tumours can be affected by both intrinsic and extrinsic stresses, such as genomic, proteotoxic, metabolic, hypoxic stress etc. In this study, we focus on genomic stress. Many solid tumours present widespread aneuploidy or DNA damage,^7^ and these extreme manifestations of genomic instability constitute a stress phenotype. Recent findings demonstrate that patients with high-mutation-burden tumours have improved survival.^8,9^ Genomic instability can in turn instigate other stress forms such as mitotic and proteotoxic stresses. Although some NOA genes have been studied extensively,^4^ they have not been identified or analysed from a systems perspective or characterised as a group that may be subdivided further.

Differential coexpression has recently been employed as a supplement to traditional differential gene expression.^10,11^ This method identifies changes of coexpression between two states, hence focusing more on the dynamics, or the ‘rewiring’ of the network interactions, as opposed to simply quantitative measures of gene expression.

We present the first systems level analysis for NOA gene and module discovery, where we have investigated 15 cancer types. We used copy number alteration (CNA) data and gene expression profiles from The Cancer Genome Atlas (TCGA) to identify significant differential coexpression modules between high and low genomic stressed tumours. We furthermore applied physical protein interactome information to identify NOA rewiring hub genes. We identified 101 candidate NOA modules and 133 candidate NOA rewiring hub genes. We highlighted four activated NOA modules and used these as examples to demonstrate the unique rewiring roles of NOA genes in the human protein interactome. We found that even though the NOA rewiring hub genes are mainly cancer-specific, they regulate highly similar neighbour genes across cancer types.

## Results

### Defining the genomic instability stress phenotype

**Fig. 1:**
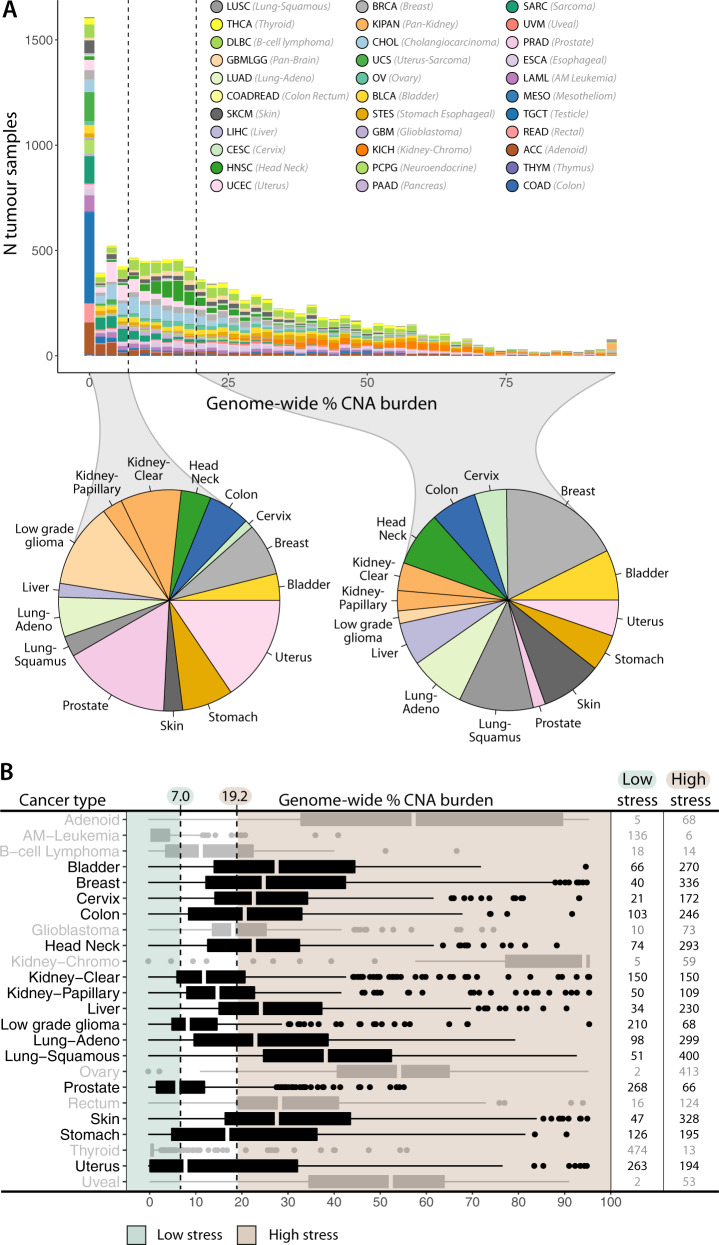
Defining stress phenotypes. **a** Genome-wide % CNA burden distribution quartiles for defining stress groups. The CNA burden distribution for all TCGA cancer types (*n* = 33) with CNA profiles (*n* = 11,034) was used to define the stress phenotype groups indicated by the dashed lines at 7.0% (low stress) and 19.2% (high stress). The pie charts represent the distribution of cancer types within the selected samples that are used for analysis (*n* = 5367) covering the 15 cancer types. Some of the cancer types are altered to match the RNA-Seq data set such as KIPAN (to Kidney-Clear and Kidney-Papillary) and STES (to Stomach). The grey cancer names are those used in this paper (for full names see Supplementary Table S1). **b** CNA burden distributions for tumour samples applied in the analyses. Of the 33 cancer types, 24 cancer types have both RNA-Seq and CNA samples, which is needed for our analyses. Furthermore, cancer types with at least 20 samples in both high and low-stress groups were included in the comparative analyses. This resulted in including 15 cancer types (black) and excluding 9 (grey). The cancer types left have in concert 3356 stress and 1601 non-stress phenotype samples. Onwards, we will refer to ‘Brain’ as LGG, since only this brain cancer type is used for further analyses

Aneuploidy and copy number alterations are known hallmarks of cancer^12^ and their frequency vary largely between tumours even within the same cancer type. Genome instability can be a side effect of oncogenic perturbations and result in an enormous stress burden for the tumour. To estimate tumour groups of high and low degree of genomic instability, we used as a proxy the CNA burden, which is the fraction of the autosomal genome that is affected by copy number alterations (both gains and losses).^13^ We calculated the CNA burden for each of the 11,034 CNA profiles available from TCGA, based on which we derived a CNA burden distribution for 33 cancer types. From the distribution, tumours were grouped into low and high-stress categories based on the first quartile and the median, respectively (Fig. 1a). We specifically applied this approach, leaving out samples with medium-level CNA burden, to perform differential analyses on the low- and high-end CNA cases. We thus define high-stress phenotype tumours as those with more than 19.2% genome-wide copy number variation and low-stress phenotypes as those with <7.0% genome-wide CNA.

To perform differential gene and coexpression analysis between high and low stress, we selected TCGA patients with both CNA and gene expression profiles available. In addition, cancer types with fewer than 20 samples in any of the stress groups were excluded from the comparative analysis. Analyses were thus performed on the remaining 15 TCGA cancer types comprising overall 3356 high-stress and 1601 low-stress phenotype samples, respectively (Fig. 1b). A schematic of the computational method is shown in Supplementary Fig. S1.

### Widespread loss of coexpression during genomic stress

We performed a differential gene expression analysis for the two stress conditions for all cancer types and examined the upregulated genes for NOA stress-relief processes (Log_2_ fold change ≥ 1.5 and FDR < 0.05) (Supplementary Fig. S2). A Gene Ontology (GO) enrichment analysis showed that these genes are involved in cell differentiation, multicellular development and neurogenesis amongst several cancer types. Differential gene expression analysis can complement a network-based differential coexpression analysis, and we therefore integrated the differential gene expression profiles into the subsequent analyses as additional information.

Differential coexpression analysis can be used to understand condition-specific activation of functional modules based on the idea of guilt-by-association.^14,15^ We carried out differential coexpression analysis using DiffCoEx^15^ for each of the 15 cancer types separately to identify modules that are activated by genomic stress (see Methods section for details). This method is an extended version of the Weighted Gene Co-expression Network Analysis (WGCNA) tool.^14^ Instead of calculating coexpression modules given one condition, DiffCoEx calculates the differential coexpression modules for two given conditions; in this case high and low genomic stress. The approach can thus minimise generic cancer interactions, such as proliferation-related modules, which can be non-specific for the condition in question.^16^

**Fig. 2:**
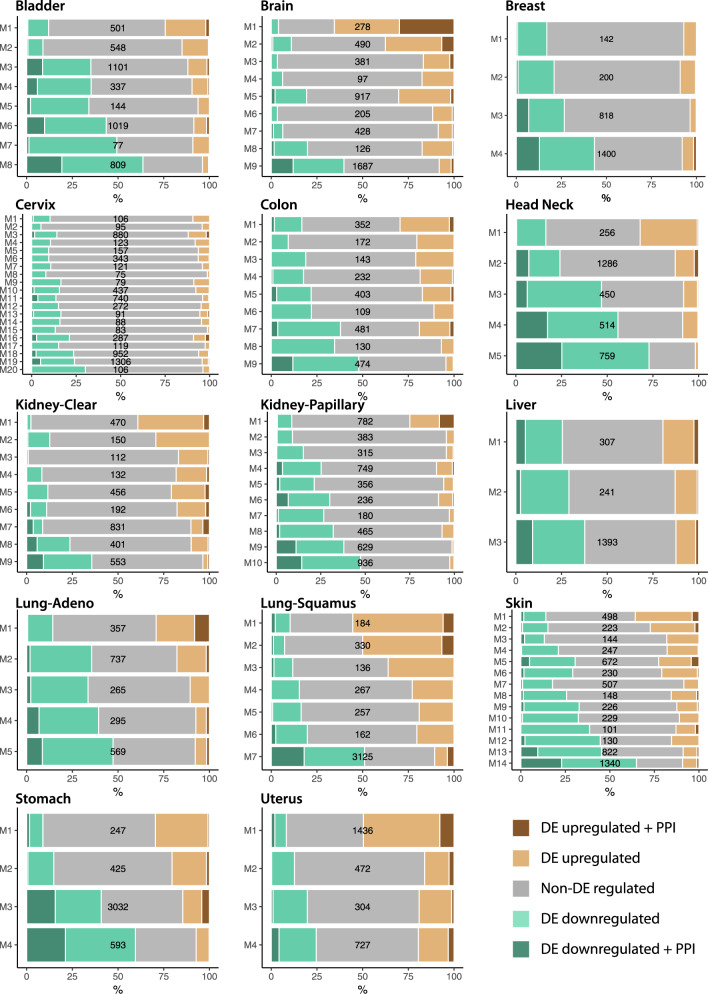
High stress differential coexpression modules across 14 cancer types. Differential coexpression modules were obtained for high stress vs. low-stress samples using differential coexpression analysis^14,15^ for 15 cancer types. No modules were found for prostate. The differential coexpression modules were mapped to a physical human protein interactome.^19^ In addition, differential gene expression was performed on the same stress groups. The brown and turquoise fractions illustrate significant differentially up or down expressed genes, respectively. Darker areas are genes that participate in a physical protein–protein interaction. Grey areas are genes that are not differentially expressed. Numbers within each bar are the number of genes per module. DE: differential expression; PPI: protein–protein interaction

We used this strategy to identify differential coexpression modules for all cancer types. Genomic CNA levels can correlate with coexpression changes, which can give rise to results linked to altered stoichiometry and not the stress response itself. To avoid this, we performed differential coexpression analyses only for genes uncorrelated for copy number and gene expression (see Methods for details). No significant modules were found for prostate cancer, which is unsurprising, since it is one of the cancer types with lowest mutation burden profile^17^ (Fig. 1b). A total of 125 differential coexpression modules were identified across the 14 cancer types ranging in size from 75 to 3125 genes with a median of 315 genes (Supplementary Fig. S3). We found that 100 of the 125 differential coexpression modules lose coexpression in the high stress compared with the low-stress condition. Cervical and skin cancers have the highest numbers of differential coexpression modules, 21 and 15, respectively, whereas liver cancer represents the minimum with four modules. To acquire more knowledge on the functional properties of these modules, we investigated both the differential gene expression and physical protein–protein interactions within the modules (Fig. 2). Here we observe that even though cervical cancer has the most differential modules, these were comprised of few differentially expressed genes and physical interactions.

To assess the robustness to subsampling, we performed a fivefold cross validation test where each cancer type was subsampled using 90 and 70% of the data. For the 90% cross validation analysis, we obtained gene set overlaps ranging from 61 to 94% and for the 70% cross validation gene set overlaps from 59 to 93% (Supplementary Fig. S4).

Single-gene differential expression can detect upregulated NOA genes within modules. Despite the massive loss of coexpression in the high-stress state, single genes within the module can rewire and participate in other functional modules. The human protein interactome has, for example, been shown to be dominated by many weak stoichiometric interactions and thus be highly dynamic.^18^ Moreover, since many proteins exert their function in protein complexes, physical protein–protein interaction modules can be useful to deduct functional knowledge on novel modules. Hence, we mapped the stress-specific differential coexpression modules to an annotated and high-quality human interactome InWeb_InBioMap (InWeb_IM)^19^ obtaining 101 condition-specific differential coexpression modules with physical protein–protein interactions (with a minimum module size of five) (Supplementary Table S2). The modules range in size from 5 to 1651 proteins.

### Single-gene rewiring in differentially activated stress modules

It can be considered that stress-relieving NOA genes behave as multifunctional hub genes capable of rewiring and participating in several functionalities when dealing with fluctuating stress levels.^20^ Therefore, we are especially interested in genes that are differentially upregulated themselves while having several deregulated physically interacting neighbours. These were the most promising candidates for regulators that shift functionality when experiencing stress. We defined the degree of rewiring as the number of genes that (1) undergo significant differential gene expression and (2) physically interact with the NOA rewiring hub gene. We defined rewiring genes as genes that were significantly upregulated (FDR < 0.05, Log_2_FC ≥ 0.5) and have a degree of rewiring of at least three. We defined the latter threshold because our stringent definitions of NOA rewiring interactions leads to a very sparse network. In addition, three is the minimal number of interactions for a hub protein to configure a functional submodule.^21,22^ We thus ended up obtaining 133 NOA rewiring hub genes in total (Supplementary Table S3). To compare these genes to previous findings, we searched the literature for each gene and found associations to the NOA mechanism for 26% of them (Supplementary Table S4). These are enriched for functions such as chromosome maintenance, oxireductase activity, mitotic checkpoint, DNA replication initiation and synaptic signalling. Surprisingly, 19% of the genes take part in synaptic signalling, which may be due to brain cancer being the cancer type with most NOA rewiring hubs. However, only two genes overlap between the 25 synaptic genes and 44 brain cancer rewiring hubs.

**Fig. 3:**
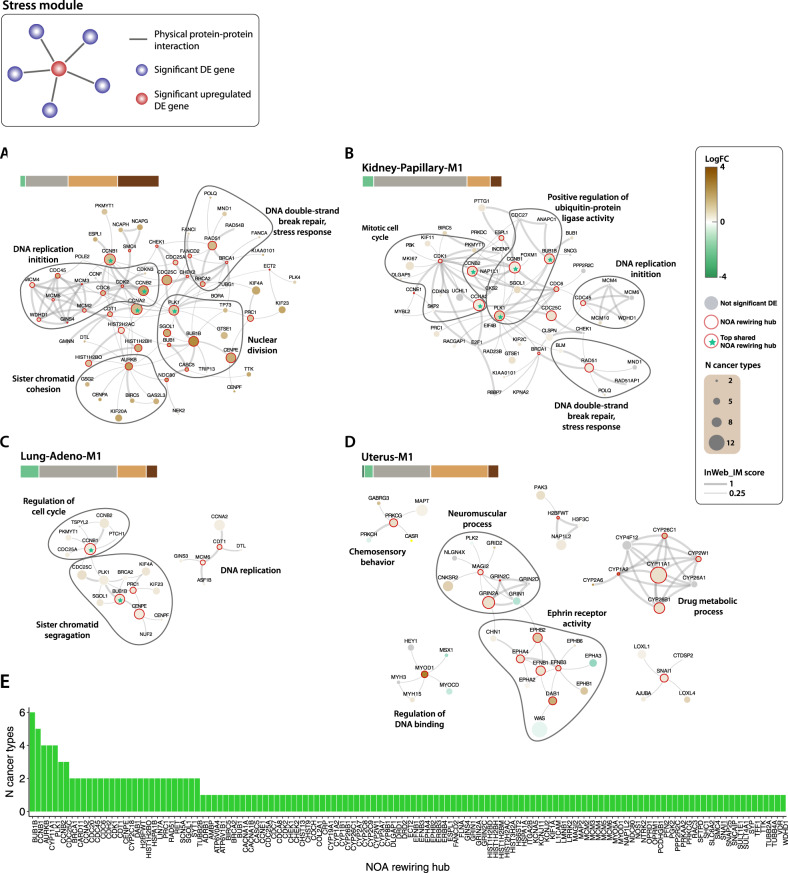
Four stress-activated modules. We selected the four modules with the highest fraction of upregulated genes with physical interactions. Here, we show the NOA rewiring hubs with their first-level interactors. The modules are enriched for cell stress maintenance functions such as DNA double-stranded break repair, stress response, chromatid segregation and DNA replication regulation. The sizes of the nodes are the number of differential modules that the gene is part of across cancer types. Grey nodes indicate that these genes were not significantly differentially expressed. **a** Brain module 1. **b** Kidney-Papillary module 1. **c** Lung-Adeno module 1. **d** Uterus module 1. **e** LogFC: Log_2_ fold change

The four modules with highest fraction of upregulated genes in their physically interacting subsets are Brain-M1, Kidney-Papillary-M1, Lung-Adeno-M1 and Uterus-M1 (Fig. 3a–d). From these, we extracted the NOA rewiring hub genes and their first-order interacting neighbours to functionally examine. These were functionally annotated using the gene enrichment tool gProfileR^23^ revealing that they take part in mitotic checkpoint, DNA repair and chromatin segregation regulation processes. We also found regulation of ubiquitin-ligase activity, Ephrin receptor activation and oxireductase activity. Ephrin and Ephrin receptors have been linked to tumorigenesis, but bidirectional roles and divergent expression patterns have been observed across tumours.^24^ This could imply a stress-buffering role, since their multifunctional characteristics benefit from the fluctuating stress environments that tumours experience.

Some rewiring hubs are found in stress modules across several cancer types. For example, we observe that *PLK1, CCNB1/2, CCNA2, AURKB* and *BUB1B* are shared across 6–9 cancer types (Fig. 3e) and physically interact with several deregulated neighbouring genes in our activated modules (Fig. 3a–c). PLK1, AURKB and BUB1B are mitotic kinases and regulatory genes during mitotic cell cycle and especially PLK1 and AURKB have been studied as promising NOA drug targets.^2,25^ PLK1 is a multifunctional protein during cell cycle following DNA damage^26^ regulating both mitotic entry/exit, DNA damage checkpoint, spindle formation, etc. BUB1B is involved in the spindle assembly checkpoint and ensures proper chromosome segregation. Recent studies highlight that BUB1B dependency is especially elevated in brain cancer.^27,28^ This is supported by our analysis showing that BUB1B is the most upregulated NOA rewiring hub in brain cancer.

### Overlapping stress-activated genes across cancer types

**Fig. 4:**
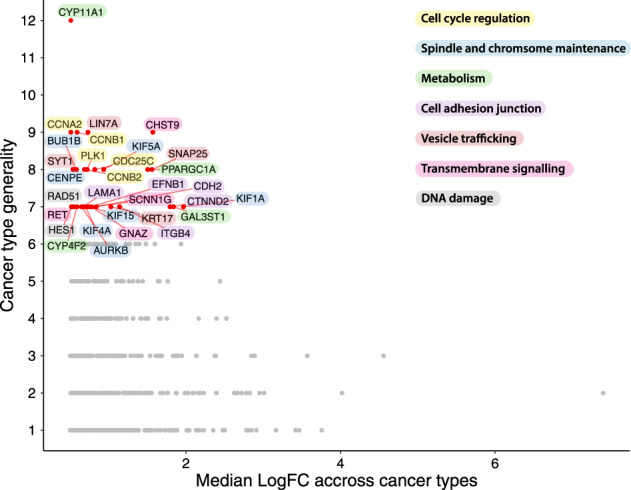
Upregulated stress module genes across cancer types. The generality of upregulated stress module genes is shown across cancer types. Genes with Log_2_FC ≥ 0.5 participating in more than six cancer types (differential modules) are labelled (see Supplementary Table S5 for rest). The functions of the genes have been grouped into seven main categories illustrated by the colours of the gene labels

Genes found in more than one cancer type may be generically activated genes essential for stress response and can function as targets for broader therapeutics that are not specific for a certain cancer type. To acquire an overview of genes participating in more than one tumour, we counted the number of times a gene was found in the differential modules across cancer types. To estimate the expression activity for each gene, we used their median LogFC across cancer types (Supplementary Table S5). Since NOA genes are often upregulated to uphold a robust cancer state, we looked into the functionality of genes with high differential expression (Log_2_FC ≥ 0.5) that are also shared across more than six cancer types. These are mainly involved in cell cycle regulation, spindle and chromosome stabilisation, cell adhesion junction, transmembrane signalling, metabolism or synaptic processes (Fig. 4). Interestingly, several of these genes (*BUB1B, CDC25C, CENPE*, kinesin and *CCNB1*) are involved in the PLK1 signalling pathway, which is a regulator of several DNA damage control and genomic instability processes. We found that the hub genes (*BUB1B, CDC25C, CENPE*, kinesin and *CCNB1*) make up an essential response across many cancer types. More studies are needed to validate this as a novel NOA pathway. We also identified a group of upregulated synaptic genes involved in vesicular trafficking (*LIN7A, SYT1* and *SNAP25*). This is consistent with mounting evidence showing that cancer cells depend on intercellular communication via the release of extracellular vesicles for viability purposes. Extracellular vesicles have been shown to contain oncogenes and RNAs and are proposed to have a mechanism of transport as neurocytological transport.^29^

### NOA rewiring hubs regulate similar neighbours across cancer types

**Fig. 5:**
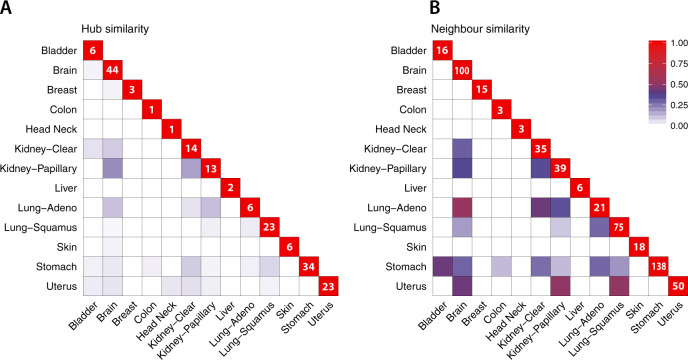
Rewiring characteristics across cancer types. **a** Rewiring gene similarity. The overlaps between NOA rewiring hub genes were calculated using the Jaccard index. Numbers in the diagonal represent the number of NOA rewiring hub genes per cancer type. **b** Neighbour similarity. The overlaps between neighbour genes of NOA rewiring hub genes were calculated using the Jaccard index. Numbers in the diagonal represent the number of neighbour genes of the NOA rewiring hub genes per cancer type

Next, we investigated the amount of overlap of the NOA rewiring hubs across cancer types to establish whether the stress response is cancer type-specific. For this analysis we used the Jaccard index as a measure of overlap between lists of hubs for each cancer type pair (Fig. 5a).

Brain, stomach, lung-squamous and uterus cancer are the cancer types with most rewiring hubs and neighbours. The cancer types kidney, lung, breast and brain cancer have the highest overlap of NOA rewiring hubs. However, most of the cancer types have low hub overlap with an average Jaccard index of 0.07 (the average of all gene set comparisons within a cancer type). The most shared rewiring hubs are BUB1B (brain, lung-adeno, kidney-clear, kidney-papillary and stomach cancer), CYP11A1 (bladder, brain, kidney-clear and uterus cancer) and CCNB1 (brain, kidney-papillary, lung-adeno and lung-squamous cancer). BUB1B is involved in mitotic checkpoint and is in normal tissue enriched in testis. Recent studies have shown that it interacts with known NOA genes, PLK1 and APC/C, to induce delay of anaphase due to spindle defects.^30,31^ CYP11A1, mostly abundant in testis and adrenal gland, is involved in drug biotransformation and could be an indicator of cancer therapy responses. Finally, the cyclin CCNB1 involved in cell cycle regulation is expressed in all tissues. We searched the human pathology atlas^32^ to verify if the upregulation of these genes are negatively correlated with survival. Indeed, both upregulation of BUB1B and CCNB1 were found to be a marker for unfavourable prognosis across several cancer types (Supplementary Fig. S5). Next, we investigated if the rewiring hubs, despite their low overlap, rewire the same group of neighbouring genes. Hence, we calculated the Jaccard index between lists of neighbouring genes of the hubs. The requirements of being a rewired neighbouring gene is that the gene needs to be significantly differentially expressed and physically interact with the rewiring hub. We found that the overlaps of the rewired neighbour genes are higher with an average Jaccard index of 0.31 (Fig. 5b) (Supplementary Table S9). To ensure that the overlap is significant, and not due to topological characteristics of InWeb_IM, we permuted InWeb_IM 1000 times. We generated 1000 random subnetworks using a within-degree node label permutation approach, where the subnetworks kept their degree distribution but had randomly re-assigned nodes. This showed that none of the permutation rounds achieved a higher overlap score than our finding (Supplementary Fig. S6). This observation suggests further that each cancer type activates distinct rewiring hubs. However, during stress, these hubs affect similar pathways.

### Validation using external datasets

To validate our stress modules, we obtained two breast cancer datasets from METABRIC (Molecular Taxonomy of Breast Cancer International Consortium),^33^ which included samples with both gene expression data and CNA profiles. The two datasets comprised of *N*_1_ = 997 (METABRIC discovery data set) and *N*_2_ = 995 patients (METABRIC validation data set). For data set 1 we obtained 336 high-stress phenotype samples and 319 low-stress phenotype samples. For data set 2 we obtained 300 high-stress phenotype samples and 375 low-stress phenotype samples.

Although the breast cancer data set was not the optimal cancer type for validation, due to the limited results (four modules), we did obtain a significant overlap of stress module genes between the TCGA set and both the METABRIC datasets (Supplementary Fig. S7). We furthermore validated two (AURKB and CCNB1) of the five NOA rewiring hub genes.

To explore the essentiality of our NOA rewiring hubs, we used the Achilles CRISPR-Cas9 data set from the Broad Institute. The data set provides gene dependency scores for 341 cancer cell lines that have been systematically perturbed. The gene dependency score reflects the gene knock down viability effects of cell lines when screened using an shRNA/siRNA library.^34^ We found that the NOA rewiring genes have a significantly higher dependency than the average gene in the Achilles data set with a *P* = 4.75e−05 (Supplementary Fig. S8). We, furthermore, performed a permutation test, where the Achilles data set were randomly sampled 10,000 times by gene sets of 128 genes. For each sampling set, we calculated the mean of dependency and recorded whether this was below the mean of the NOA hubs gene set (lower dependency than the NOA set). For none of the rounds, this was the case (*P* < 0.0001).

## Discussion

Tumour states seem to be largely robust despite a disrupted homoeostasis, compared with their normal counterpart, suggesting that they rewire to obtain a new state of homoeostasis.^3,22^ A generic buffering mechanism against stresses can be suggested since most tumours have highly diverse mutation profiles, but more similar expression profiles.^35^ In this study, we present the first systems-based method to understand NOA genes and pathways conditioned upon the intrinsic stress: genomic instability. We focused on genomic stresses, including DNA damage stress, proteotoxic stress and mitotic stress, but the approach can be extended to other types of stresses as well. We used the publicly available TCGA tumour profiles (CNA and RNA-Seq data) for 15 cancer types and grouped samples into high and low genomic stress based on their CNA burden.

Most studies have focused on gene-centric mutation-driven NOA genes (synthetic lethality), where perturbation of a specific gene, e.g. an oncogene, promotes the dependency on a NOA gene. An example is PARP1 inhibitors used to treat BRCA2-deficient tumours.^36^ Since BRCA2 tumours have a deficiency in the DNA repair pathway, they are even more dependent on PARP1 for DNA repair. A separate type of NOA genes is independent of any oncogenic mutation;^37^ these genes are instead activated when the cell is burdened by an generic cellular stress. An example is HSF1, a transcription factor that controls heat-shock proteins and is activated upon proteotoxic stress. HSF1 deficiency in mice and in cancer cell lines lower tumourigenesis.^38^ HSF1 provides essential stress relief through proteasome-mediated protein degradation pathways by induction of heat-shock proteins, such as HSP90, which is overexpressed in many cancers.^39,40^

We performed comparative analyses obtaining differential coexpression modules for the high-stress groups for each of the 15 cancer types. Most of the modules were negatively coexpressed compared with the low-stress group, suggesting that these rewire into other modules with processes more needed for the new burdened tumour state. We mapped the modules to physical interactions and gene-specific differential gene expression values and identified 101 modules and 133 rewiring hubs related to genomic instability. We found that the rewiring hubs are mainly enriched for processes involved in chromosome maintenance, oxireductase activity, mitotic checkpoint, DNA replication initiation and synaptic signalling. Moreover, 19% of the rewiring hubs are involved in synaptic signalling, which can be related to the fact that brain tissue has a higher level of gene expression compared with other tissue.^41^ However, recent evidence corroborates that (1) cancer cells can repurpose neuronal communication mechanisms to fuel tumourigenesis^42^ and (2) that there is no association between high mutation burden and improved survival in brain cancer, unlike other cancer types.^8^ In contrast to classical oncogenic targets, which are essential for tumour transformation events, our NOA rewiring hubs and modules are involved in maintenance functions for homoeostasis purposes; some of which are comparable to the effects of tumour suppressor genes. We found that despite the small overlap of hub rewiring genes across tumour types, the overlap of their respective neighbours is much larger. This suggests that stress-activated mechanisms are shared across cancer types.

Thus, increasing efforts are being directed towards mapping out cancer dependencies for example by systematically performing genome-wide vulnerability screens on large numbers of cancer cell lines,^34,43,44^ or by using advanced computational algorithms.^45^

Limitations arise when working with collected tumour data that are not optimised for tumour stress analysis. For example, TCGA samples can be highly heterogeneous and co-vary with tumour stage, other stresses, treatment effects, gene expression etc. To measure tumour stress environments, more tailored technologies and strategies are more fitting. For example, single-cell technology has the potential to measure varying levels and types of stresses across subpopulations of cells.^46,47^ Other examples are synthetic dosage lethality, in which gene dosage is taken into account, or conditional synthetic lethality that takes into account the tumour microenvironment.^6^ Another limitation is that the NOA hubs need further experimental and clinical tests to validate their causal effects as drug targets.

Few experimentally validated NOA genes exist that have been associated with related stresses, let alone entire databases. Based on a manual literature search, we found that the 26% of the NOA rewiring hubs have been associated to the NOA mechanism. Some of the NOA rewiring hubs, such as AURKB,^48,49^ PLK1,^50,51^ tubulins, CHEK1/2^52^ and HSPA1B,^53^ are already being considered and tested as promising cancer therapeutics. The general mechanisms behind these are either to exploit a ploidy-burdened phenotype by inducing ploidy overload (AURKB, PLK1, tubulins, CHEK1/2) or to reduce levels of stress-reflief proteins (HSPA1B). Aurora kinases, such as AURKB, are undergoing phase III clinical trials in various cancer types due to their multifunctionality and promising effects when treated in combination with, e.g. chemotherapy.^54^

Our findings identify 101 genomic stress-activated NOA modules comprising of 133 NOA rewiring hub genes across 14 cancer types. Our method shows that activated NOA modules are enriched for homoeostasis maintenance functions, such as PLK1 signalling, DNA repair and spindle and chromosome maintenance. Finally, we observe the tendency that NOA rewiring hub genes share many neighbours across cancer types suggesting a generic stress response. We suggest that these shared neighbour genes could be important candidates for use in combinatorial therapy together with cancer-specific oncogenes as a step towards cancer precision medicine. The approach provides the first comprehensive view of condition-specific NOA genes and can pave the way to better understanding of the NOA concept in future studies. We believe that the next era of cancer therapeutics will exploit intrinsic and extrinsic conditional vulnerability mapping to leverage how tumour stresses can be utilised for drug targeting.^6^

## Methods

### Data

Copy number alteration (CNA) data (*n* = 11,034) were obtained from The Cancer Genome Atlas (TCGA) (https://cancergenome.nih.gov/) on 7 April 2017. TCGA RNA-Seq data (raw read counts) were obtained from the GEO (Gene Expression Omnibus) (https://www.ncbi.nlm.nih.gov/geo/) data set GSE62944^55^ as input to the pre-processing and differential gene expression analysis in DESeq2.^56^ From the literature we manually curated a list of experimentally validated non-oncogene addiction genes using full article texts (Supplementary Table S4).

### Data pre-processing

Genes with zero variance and a low raw count (<10 in more than 90% of the samples) were excluded from the analysis as recommended by the WGCNA pipeline authors for pre-processing of RNA-Seq data (https://labs.genetics.ucla.edu), as they tend to reflect noise. This also ensures that genes not expressed in a certain cell type are left out of the comparative analysis. DESeq2 (version 1.10.1)^56^ was used to perform pre-processing on the raw RNA-Seq counts. The DESeq2 function varianceStabilizingTransformation^57^ is a global normalisation approach performed to minimise variation caused by technical errors. Unwanted variation in the data, such as age and sex, were corrected for using the svaseq function implemented in the SVA R package (version 3.18.0).^58^ Age and sex information on the TCGA samples are available at firebrowse (http://firebrowse.org/). We furthermore corrected the breast cancer samples for PAM50 subtypes using svaseq.

Genes with a significant RNA-Seq copy number correlation were removed from the analysis to avoid confounding effects due to stoichiometric alterations. This was done from pre-calculated TCGA correlation tables available at firebrowse (version 2016_01_28). The correlation coefficients were calculated based on the Log_2_ CNA and RNA-Seq expression for each corresponding feature using Pearson correlation (“Correlations between copy number and RNA-Seq expression,” 2016) (http://firebrowse.org/). We used genes that did not show significantly RNAseq-CNA correlation, thus genes with a Benjamini and Hochberg-corrected *q*-value > 0.05.

### Defining low and high-stress groups

Pre-processed TCGA mutation and copy number segment tables were used to group tumour samples into stress and non-stress phenotypes. Copy number segment tables were used to calculate the percentage-wise base pairs affected by CNA for each tumour.

1 $${\mathrm{CNV}}\,{\mathrm{burden}} = \frac{{{\sum} {n_{{\mathrm{loss}}}} + n_{{\mathrm{gain}}}}}{{n_{{\mathrm{autosome}}}}}$$

To calculate the percentage-wise CNA burden, segments of CNA gains and losses were determined, and their total genomic length (in number of base pairs: *n*_loss_ and *n*_gain_) was summed and calculated as a percentage of the size of the autosomal genome (*n*_autosome_).^13^ The autosomal genome length was calculated from NCBI’s Build 36 (hg18) from UCSC (https://genome.ucsc.edu/), since this is the reference used for the TCGA CNA SNP-array data. We excluded CNAs with a segment mean in the range −0.2 < segment mean < 0.2 to provide a threshold for when to infer gain and loss of CNAs.^59^ Segment mean is the Log_2_ ratios of the tumour copy number to the normal copy number.

All TCGA tumour samples (*n* = 11,034) were considered when defining the thresholds for low and high mutation burden samples. Our definition of the low-stress phenotype includes those samples presenting a CNA burden equal or lower than the upper boundary of the first quartile. Similarly, the high-stress phenotype was defined as those presenting a CNA burden above the lower limit of the medium quartile. Only low and high mutation burden samples were used for further stratification. The exact thresholds for the low and high group are 7.0% and 19.2% CNA burden, respectively.

### Differential gene expression analysis using edgeR

Raw counts were used as input to edgeR^60^ to perform differential gene expression analysis. The data were normalised using the edgeR functions calcNormFactors and afterwards, dispersion estimates were done using the functions estimateGLMCommonDisp and estimateGLMTagwiseDisp. In the model, we corrected for covariates age and sex. Finally, we used glmFit to perform the differential analysis between stresses and obtained results with FDR < 0.05 to acquire significantly differentially expressed genes in the high-stress state. Genes were analysed for enriched GO Biological Processes using gProfileR^23^ in R.

### Differential coexpression modules using DiffCoEx

Differential coexpression analysis was performed for the high versus low CNA burden groups using the R implementation of DiffCoEx.^15^ DiffCoEx uses the WGCNA statistical framework^14^ to identify differential coexpression modules. We implemented the topological overlap measurement (from the WGCNA framework) when identifying coexpression modules, which takes into account the interconnectedness of the network when performing correlation analysis. The approach can be useful to exclude spurious or isolated connections during network construction and is more robust than pairwise correlation alone for clustering genes by similarity. The differential coexpression measurements are transformed into dissimilarity scores between genes, and then hierarchical clustering is used to detect gene modules. The hierarchical clustering was applied using the dynamic tree cut method. The soft threshold β is a parameter used to scale the correlation networks such that the weights of larger correlation differences are emphasised in comparison with lower ones. These were calculated for each of the 15 cancer types using the function pickSoftThreshold from the WGCNA package. A minimum module size of 30 was used to exclude small modules in the results. Besides the mentioned settings, default parameters from WGCNA were used for the clustering.

To access the significance of the differential coexpression modules, we performed a permutation procedure (included in the DiffCoEx package) using dispersion statistics. For each DiffCoEx run, samples were permuted *n* = 1000 times between the two stress conditions to generate a null distribution from which we could evaluate the significance of the modules. A dispersion value was calculated for each module, which is a measure of the correlation change for a group of genes. The *p*-value was calculated based on the number of times a permutation yielded a higher dispersion than the dispersion value of our module. The permutation was done using a function from DiffCoEx using the R framework.^15^

### Physical differential coexpression modules and rewiring hubs

The differential coexpression modules were then mapped to the scored physical human interactome, InWeb_IM.^19^ These modules were selected if they have a minimum of five genes. Genes were assigned as a NOA rewiring hub if they are significantly upregulated (FDR < 0.05, Log_2_FC ≥ 0.5) and have at least three physically interacting genes that are significantly differentially regulated (FDR < 0.05).

### Functional analysis

The differential coexpression modules mapped to physical protein–protein interactions from InWeb_IM were annotated in Cytoscape. Due to the large sizes of these modules, we furthermore used the MCL clustering algorithm to obtain smaller submodules for functional analysis. To gain functional knowledge for these submodules, we used gProfileR (version r1732_e89_eg36)^23^ to perform gene enrichment analysis on Gene Ontology (GO) Biological Processes (BP) terms. GO BP terms with highest significant FDR-corrected *p*-values (FDR < 0.05) were used.

### Protein interaction data

InWeb_IM is a scored and benchmarked protein–protein interaction (PPI) database, including interactions from humans and model organisms (human orthologs) collected from various resources.^19^ The InWeb_IM score is based on metrics such as reproducibility of the interaction data. The collection (version 2016_02_05) consists of 504,608 unique interactions covering 17,104 proteins. Only high-confident InWeb_IM interactions with scores higher than the author-recommended cutoff of 0.156 were used in the analyses covering 56,750 interactions and 10,669 proteins.

### Cross validation and significance testing

We performed a fivefold cross validation for each cancer type to assess the robustness of our results. We randomly subsampled each of the sample groups (of high and low stress) and repeated this five times for each cancer type. We performed both a 90 and 70% subsampling. We then calculated the significance of the gene overlap compared with the original results using the Fisher’s exact test for the hypergeometric distribution implemented in the R function phyper.

We tested the significance of NOA shared neighbour overlaps by performing a within-degree node label permutation. This approach generated permuted networks that kept their degree distribution but randomly re-assigned the nodes. We built 1000 random networks and for each of these, we calculated the neighbour overlaps of the modules and compared with the average value of our neighbour overlaps (for the NOA hubs across all cancer types).

### Validation using the METABRIC cohort

Normalised gene expression data and segmented copy number profiles for two METABRIC breast cancer cohorts (*N*_discovery_ = 997 and *N*_validation_ = 995) (EGAS00000000083) were downloaded from the European Genome-phenome Archive (https://www.ebi.ac.uk/ega/). After removing cases with NAs, the validation set was reduced to 987 samples.

We adjusted the gene expression data for age and breast cancer subtypes (PAM50) using svaseq. We repeated our analysis pipeline for 336 high-stress phenotype samples and 319 low-stress phenotype samples for the discovery set and 300 and 375 for the validation set, respectively.

### Validation using Project Achilles

The Achilles CRISPR data set (Avana-17Q2-Broad_v2) was downloaded from the Broad Institute data set portal (https://portals.broadinstitute.org/achilles/).

This data set includes 341 cancer cell lines, for which the individual genes have been systematically perturbed using CRISPR-Cas9 to identify their effects on survival. Hundred and twenty-eight NOA rewiring hubs were present in the Achilles data set. We performed a permutation test, where the Achilles data set were subsampled 10,000 times with a sample size of 128 genes to assess the significance of the Achilles score for our NOA rewiring hubs. We also performed a Wilcoxon rank sum test to test if the NOA Achilles score distribution differed from the average Achilles score distribution.

### Additional resources

The protein interaction data set, InWeb_IM, is available at http://www.lagelab.org/resources/. TCGA CNA and RNA-Seq profiles are available at https://cancergenome.nih.gov/. TCGA raw read count RNA-Seq data are available at https://www.ncbi.nlm.nih.gov/geo/. TCGA RNAseq-CNA correlation tables are available at http://firebrowse.org/. The Project Achilles data set is available at https://portals.broadinstitute.org/achilles. The METABRIC datasets can be applied for through https://www.ebi.ac.uk/ega/.

### Reporting summary

Further information on research design is available in the Nature Research Reporting Summary linked to this article.

## Supplementary information

Reporting Summary

Supplementary material

## Data Availability

Copy number variation data were downloaded from The Cancer Genome Atlas (TCGA) (https://cancergenome.nih.gov/) and TCGA RNA-Seq data (raw read counts) were obtained from the GEO (Gene Expression Omnibus) (https://www.ncbi.nlm.nih.gov/geo/) data set GSE6294455.

## Supplementary information

**Supplementary Information** accompanies the paper on the *npj Systems Biology and Applications* website (10.1038/s41540-019-0104-5).

## References

[1] Solimini NL, Luo J, Elledge SJ (2007). Non-oncogene addiction and the stress phenotype of cancer cells. Cell.

[2] Nagel R (2016). Drugging the addict: non-oncogene addiction as a target for cancer therapy. EMBO Rep..

[3] Califano A, Alvarez MJ (2016). The recurrent architecture of tumour initiation, progression and drug sensitivity. Nat. Rev. Cancer.

[4] Luo J, Solimini NL, Elledge SJ (2009). Principles of cancer therapy: oncogene and non-oncogene addiction. Cell.

[5] Costanzo M (2016). A global genetic interaction network maps a wiring diagram of cellular function. Science.

[6] O’Neil NJ, Bailey ML, Hieter P (2017). Synthetic lethality and cancer. Nat. Rev. Genet..

[7] Grade Marian, Difilippantonio Michael J., Camps Jordi (2015). Patterns of Chromosomal Aberrations in Solid Tumors. Recent Results in Cancer Research.

[8] Samstein RM (2019). Tumor mutational load predicts survival after immunotherapy across multiple cancer types. Nat. Genet..

[9] Singal G (2019). Association of patient characteristics and tumor genomics with clinical outcomes among patients with non–small cell lung cancer using a clinicogenomic database. Jama.

[10] Li J, Li YX, Li YY (2016). Differential regulatory analysis based on coexpression network in cancer research. BioMed Research International.

[11] Hsu C, Juan H-F, Huang H-C (2015). Functional analysis and characterization of differential coexpression networks. Sci. Rep..

[12] Hanahan D, Weinberg RA (2011). Hallmarks of cancer: the next generation. Cell.

[13] Hieronymus H (2014). Copy number alteration burden predicts prostate cancer relapse. Proc. Natl Acad. Sci. USA.

[14] Langfelder P, Horvath S (2008). WGCNA: an R package for weighted correlation network analysis. BMC Bioinforma..

[15] Tesson BM, Breitling R, Jansen RC (2010). DiffCoEx: a simple and sensitive method to find differentially coexpressed gene modules. BMC Bioinforma..

[16] Ideker T, Krogan NJ (2012). Differential network biology. Mol. Syst. Biol..

[17] Chalmers ZR (2017). Analysis of 100,000 human cancer genomes reveals the landscape of tumor mutational burden. Genome Med..

[18] Hein MY (2015). A human interactome in three quantitative dimensions organized by stoichiometries and abundances. Cell.

[19] Li Taibo, Wernersson Rasmus, Hansen Rasmus B, Horn Heiko, Mercer Johnathan, Slodkowicz Greg, Workman Christopher T, Rigina Olga, Rapacki Kristoffer, Stærfeldt Hans H, Brunak Søren, Jensen Thomas S, Lage Kasper (2016). A scored human protein–protein interaction network to catalyze genomic interpretation. Nature Methods.

[20] Dietlein F, Thelen L, Reinhardt HC (2014). Cancer-specific defects in DNA repair pathways as targets for personalized therapeutic approaches. Trends Genet..

[21] Barabási A-L, Oltvai ZN (2004). Network biology: understanding the cell’s functional organization. Nat. Rev. Genet..

[22] Hu JX, Thomas CE, Brunak S (2016). Network biology concepts in complex disease comorbidities. Nat. Rev. Genet..

[23] Reimand Jüri, Arak Tambet, Adler Priit, Kolberg Liis, Reisberg Sulev, Peterson Hedi, Vilo Jaak (2016). g:Profiler—a web server for functional interpretation of gene lists (2016 update). Nucleic Acids Research.

[24] Pasquale EB (2010). Eph receptors and ephrins in cancer: bidirectional signalling and beyond. Nat. Rev. Cancer.

[25] Wang J (2016). Suppression of KRas-mutant cancer through the combined inhibition of KRAS with PLK1 and ROCK. Nat. Commun..

[26] van Vugt MATM, Medema RH (2005). Getting in and out of mitosis with Polo-like kinase-1. Oncogene.

[27] Lee Eunjee, Pain Margaret, Wang Huaien, Herman Jacob A., Toledo Chad M., DeLuca Jennifer G., Yong Raymund L., Paddison Patrick, Zhu Jun (2017). Sensitivity toBUB1BInhibition Defines an Alternative Classification of Glioblastoma. Cancer Research.

[28] Ding Y (2013). Cancer-specific requirement for BUB1B/BUBR1 in human brain tumor isolates and genetically transformed cells. Cancer Discov..

[29] Candelario KM, Steindler DA (2014). The role of extracellular vesicles in the progression of neurodegenerative disease and cancer. Trends Mol. Med.

[30] Jia L, Li B, Yu H (2016). The Bub1–Plk1 kinase complex promotes spindle checkpoint signalling through Cdc20 phosphorylation. Nat. Commun..

[31] Baker DJ (2012). Increased expression of BubR1 protects against aneuploidy and cancer and extends healthy lifespan. Nat. Cell Biol..

[32] Uhlen M (2017). A pathology atlas of the human cancer transcriptome. Science.

[33] Curtis C (2012). The genomic and transcriptomic architecture of 2,000 breast tumours reveals novel subgroups. Nature.

[34] Tsherniak, A. et al. Defining a cancer dependency map. *Cell* **170**, 564–570 (2017). 10.1016/j.cell.2017.06.010 PMC5667678 28753430

[35] Califano A (2011). Rewiring makes the difference. Mol. Syst. Biol..

[36] Bryant HE (2005). Specific killing of BRCA2-deficient tumours with inhibitors of poly(ADP-ribose) polymerase. Nature.

[37] Olivero M (2014). The stress phenotype makes cancer cells addicted to CDT2, a substrate receptor of the CRL4 ubiquitin ligase. Oncotarget.

[38] Dai C, Whitesell L, Rogers AB, Lindquist S (2007). Heat shock factor 1 is a powerful multifaceted modifier of carcinogenesis. Cell.

[39] Gordon DJ, Resio B, Pellman D (2012). Causes and consequences of aneuploidy in cancer. Nat. Rev. Genet..

[40] Calderwood SK, Khaleque MA, Sawyer DB, Ciocca DR (2006). Heat shock proteins in cancer: chaperones of tumorigenesis. Trends Biochem. Sci..

[41] Collins FS, Lander ES, Rogers J, Waterson RH (2004). Finishing the euchromatic sequence of the human genome. Nature.

[42] Chen PH (2017). Crosstalk between CLCb/Dyn1-mediated adaptive clathrin-mediated endocytosis and epidermal growth factor receptor signaling increases metastasis. Dev. Cell.

[43] McDonald ER (2017). Project DRIVE: a compendium of cancer dependencies and synthetic lethal relationships uncovered by large-scale, deep RNAi screening. Cell.

[44] Krogan NJ, Lippman S, Agard DA, Ashworth A, Ideker T (2015). The cancer cell map initiative: defining the hallmark networks of cancer. Mol. Cell.

[45] Ryan CJ, Lord CJ, Ashworth A (2014). DAISY: picking synthetic lethals from cancer genomes. Cancer Cell.

[46] Adamson B (2016). A multiplexed single-cell CRISPR screening platform enables systematic dissection of the unfolded protein response. Cell.

[47] Shalek AK, Benson M (2017). Single-cell analyses to tailor treatments. Sci. Transl. Med..

[48] Goldenson B, Crispino JD (2015). The aurora kinases in cell cycle and leukemia. Oncogene.

[49] Carpinelli P, Moll J (2008). Aurora kinases and their inhibitors: more than one target and one drug. Adv. Exp. Med. Biol..

[50] Gjertsen B T, Schöffski P (2014). Discovery and development of the Polo-like kinase inhibitor volasertib in cancer therapy. Leukemia.

[51] Strebhardt K, Ullrich A (2006). Targeting polo-like kinase 1 for cancer therapy. Nat. Rev. Cancer.

[52] Ma CX, Janetka JW, Piwnica-Worms H (2011). Death by releasing the breaks: CHK1 inhibitors as cancer therapeutics. Trends Mol. Med..

[53] Sherman MY, Gabai VL (2014). Hsp70 in cancer: back to the future. Oncogene.

[54] Damodaran Arun Prasath, Vaufrey Lucie, Gavard Olivia, Prigent Claude (2017). Aurora A Kinase Is a Priority Pharmaceutical Target for the Treatment of Cancers. Trends in Pharmacological Sciences.

[55] Rahman M (2015). Alternative preprocessing of RNA-Sequencing data in the Cancer Genome Atlas leads to improved analysis results. Bioinformatics.

[56] Love MI, Huber W, Anders S (2014). Moderated estimation of fold change and dispersion for RNA-seq data with DESeq2. Genome Biol..

[57] Lin SM, Du P, Huber W, Kibbe WA (2008). Model-based variance-stabilizing transformation for Illumina microarray data. Nucleic Acids Res..

[58] Leek JT, Johnson WE, Parker HS, Jaffe AE, Storey JD (2012). The SVA package for removing batch effects and other unwanted variation in high-throughput experiments. Bioinformatics.

[59] Laddha SV, Ganesan S, Chan CS, White E (2014). Mutational landscape of the essential autophagy gene BECN1 in human cancers. Mol. Cancer Res..

[60] Robinson MD, McCarthy DJ, Smyth GK (2010). edgeR: a Bioconductor package for differential expression analysis of digital gene expression data. Bioinformatics.

