# Supplementary material for: Identification of hyper-rewired genomic stress non-oncogene addiction genes across 15 cancer types
Source: NPJ Syst Biol Appl. 2019 Aug 7;5:27. doi: 10.1038/s41540-019-0104-5 (PMC6685999; doi:10.1038/s41540-019-0104-5)
Supplement: Supplementary file 2 — Supplementary material [file 41540_2019_104_MOESM2_ESM.pdf]

Supplementary material

Supplementary figures

Figure S1

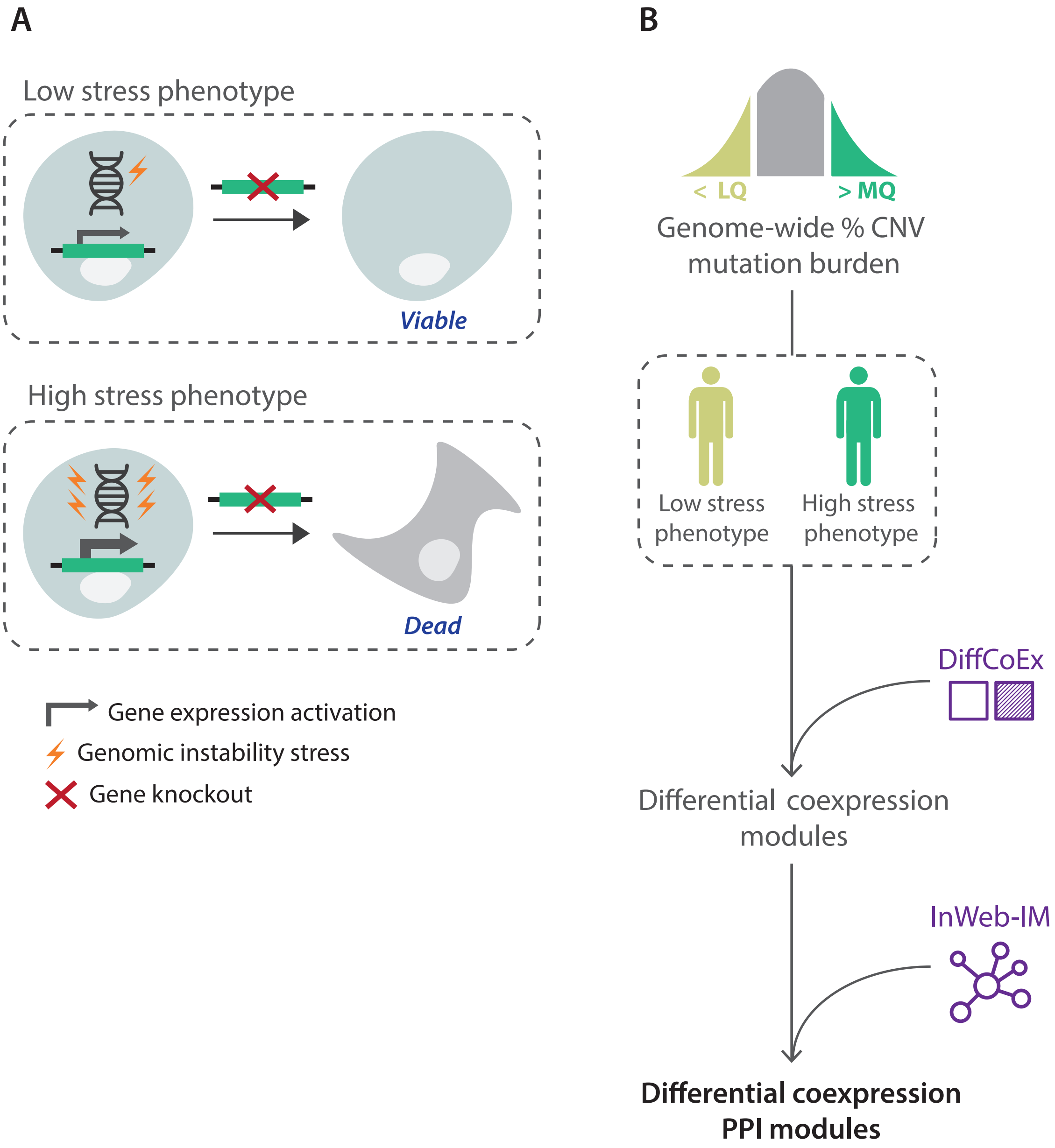

A. Non-oncogene addiction mechanism. Non-oncogene addiction (NOA) genes are activated and becomes essential for the tumour when experiencing high stress level. **B. Schematic figure of computational method.** TCGA samples are divided into high and low mutation burden (per cancer type) and DiffCoEx [15] has been applied to obtain differential coexpression networks. Afterwards InWeb was applied to these networks obtaining only physically interacting protein interactions.

Figure S2

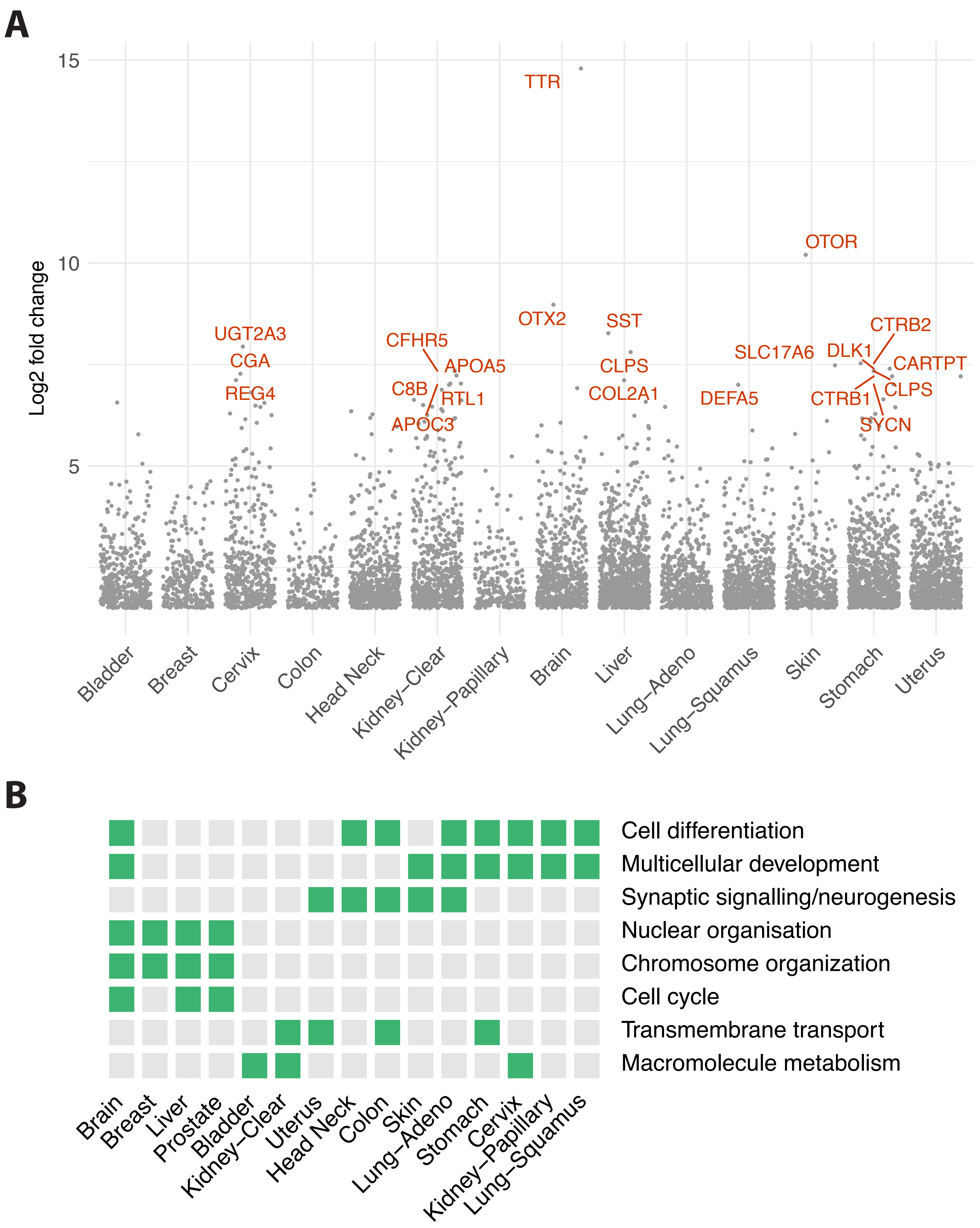

Differential gene expression analysis. (a) Upregulated genes. The scatter plot shows the top upregulated genes (Log2FC ≥ 1.5, FDR < 0.05). The red gene labels indicate genes with a $Log_2FC \geq 7$. **(b)** Upregulated GO biological processes during stress. Genes with $Log_2FC \geq 1.5$ were analysed for significant GO biological processes (FDR < 0.05). Green boxes represent cancer types with differentially upregulated genes that are enriched for a specific biological process; grey boxes represent non-enriched processes.

Figure S3

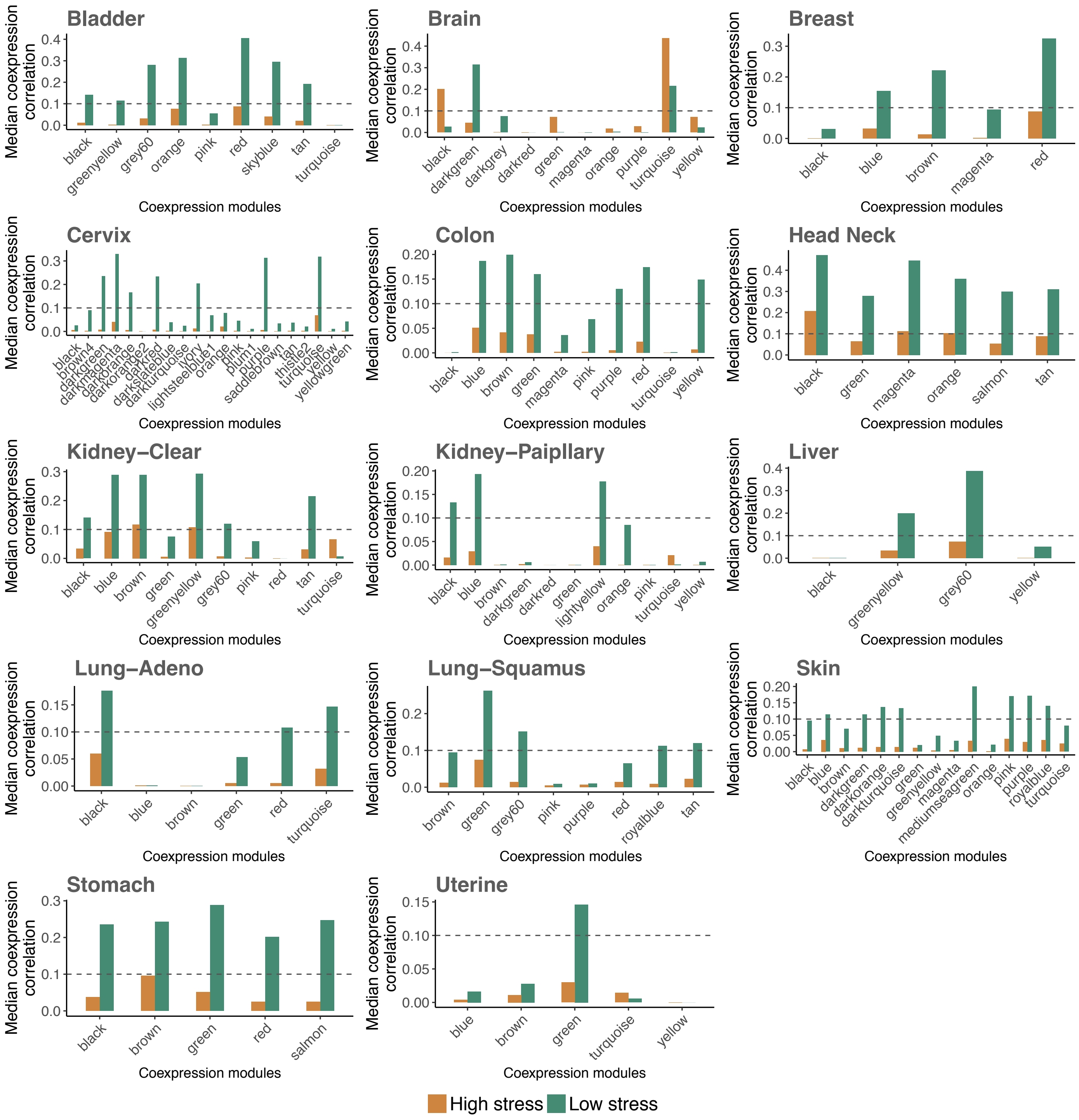

Median coexpression values of DiffCoEx coexpression modules. The dotted line indicates a median coexpression value of 0.1. Orange bars are the high stress samples, wheras the turquoise bars are low stress samples.

Figure S4

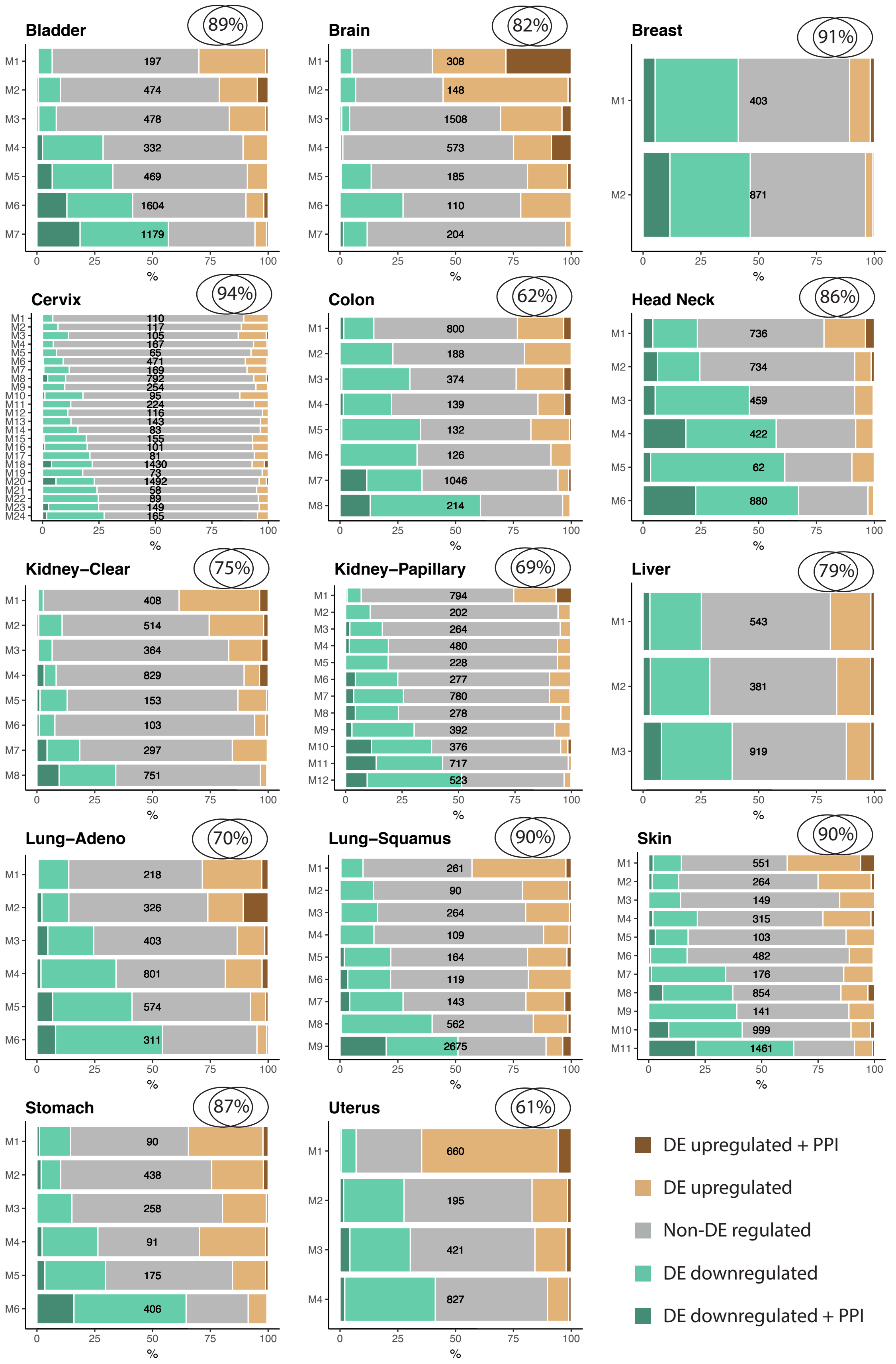

A. Cross validation using 90 % subsampling for 14 cancer types. Differential coexpression modules were obtained using for high vs. low stress sample groups that were subsampled randomly using only 90 % of the data. The Venn diagrams represent the percentage overlap of the (combined) module genes between the original results and the subsampled results. All overlaps were found to be significant using the hypergeometric test phyper in R.

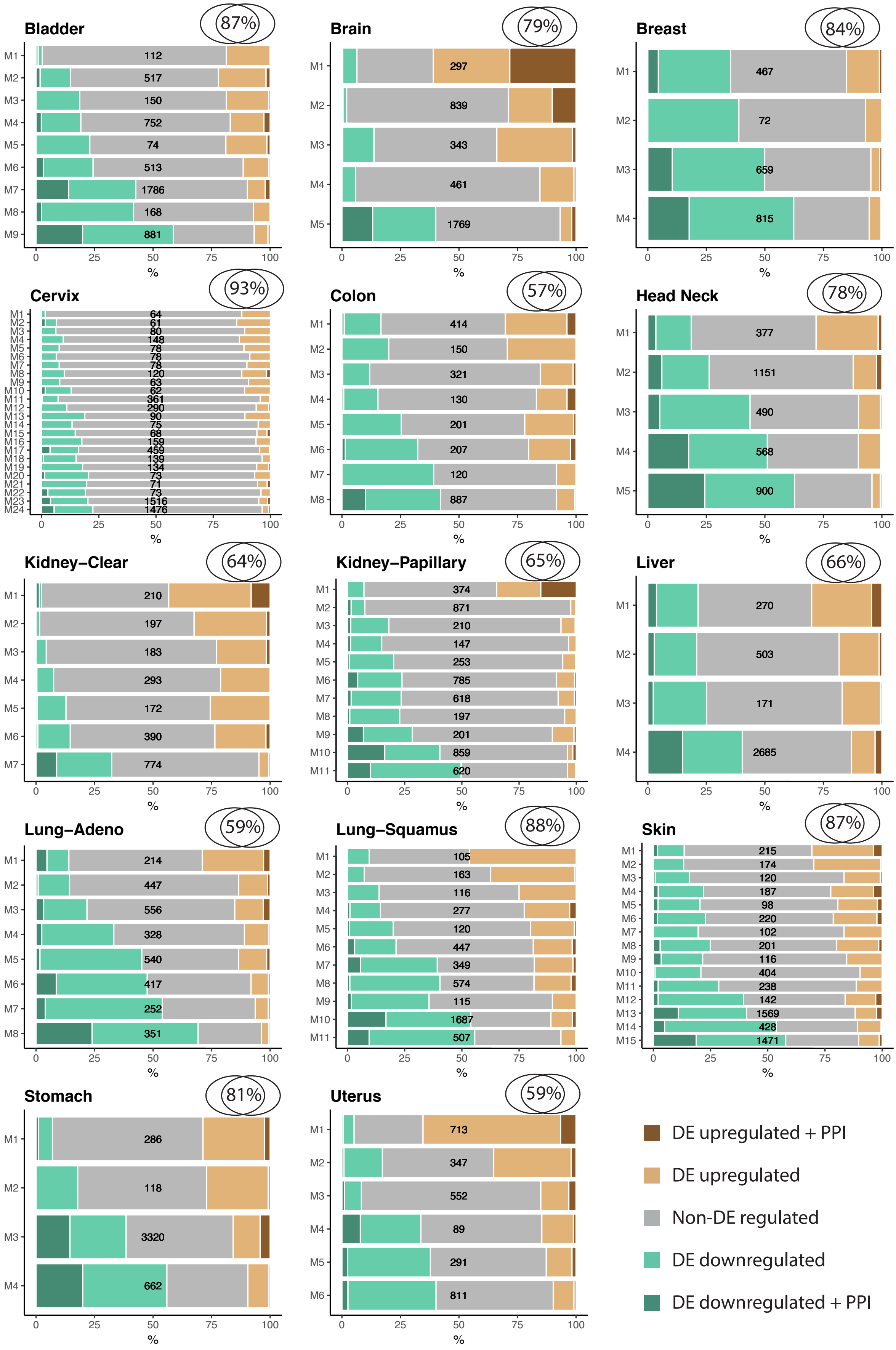

B. Cross validation using 70 % subsampling for 14 cancer types. Differential coexpression modules were obtained using for high vs. low stress sample groups that were subsampled randomly using only 70 % of the data. The Venn diagrams represent the percentage overlap of the (combined) module genes between the original results and the subsampled results. All overlaps were found to be significant using the hypergeometric test phyper in R.

Figure S5

BUB1B

Liver

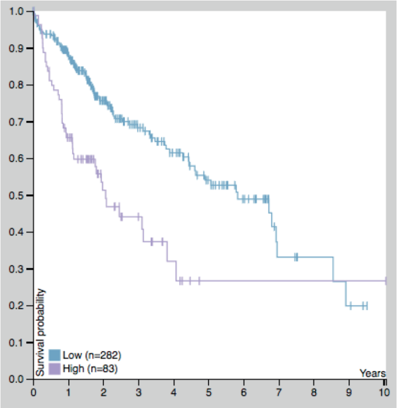

Pancreas

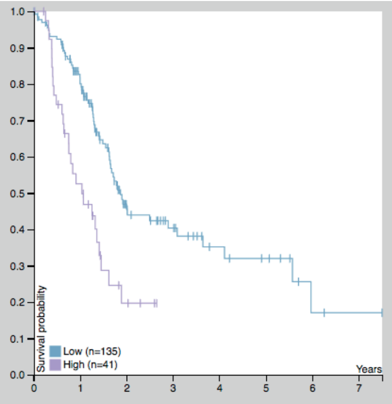

Lung

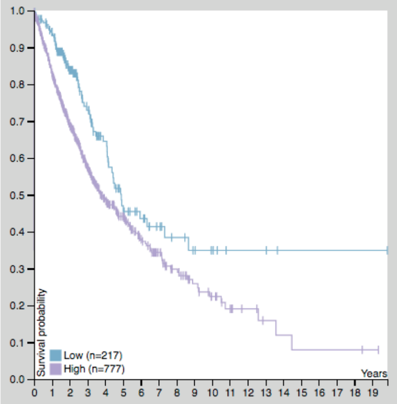

CCNB1

Liver

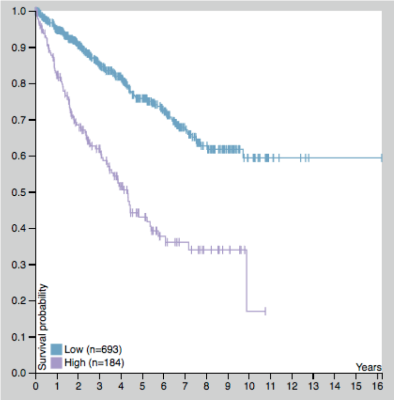

Renal

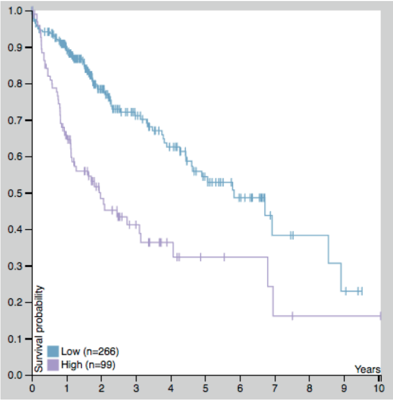

Lung

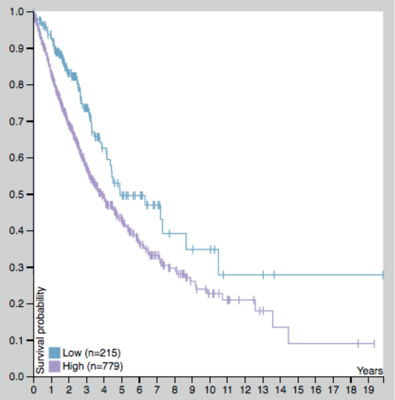

TCGA survival plots for BUB1B and CCNB1: The y-axis are percentage and x-axis years. The purple line are patients that have a high expression of the gene, whereas the blue line are patients that have low expression of the gene.

Figure S6

A

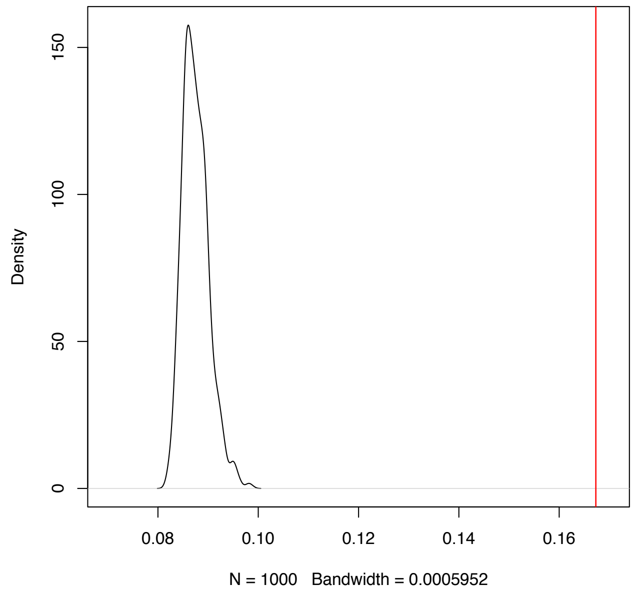

B

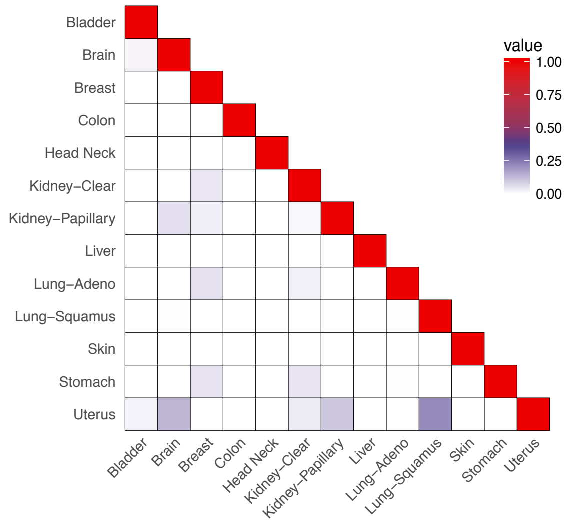

A. Distribution of neighbour overlaps across cancer types when permuting the InWeb_IM network 1,000 times. The mean of the neighbour overlap values for the NOA rewiring hubs (red) is 0.167, whereas the average of the permutated values is 0.088 (black).
B. Average of neighbour overlaps between cancer types when permuting the InWeb_IM network 1,000 times. The overlaps between neighbour genes of InWeb genes were calculated using the Jaccard index. Value: Jaccard index

Figure S7

METABRIC DISCOVERY SET

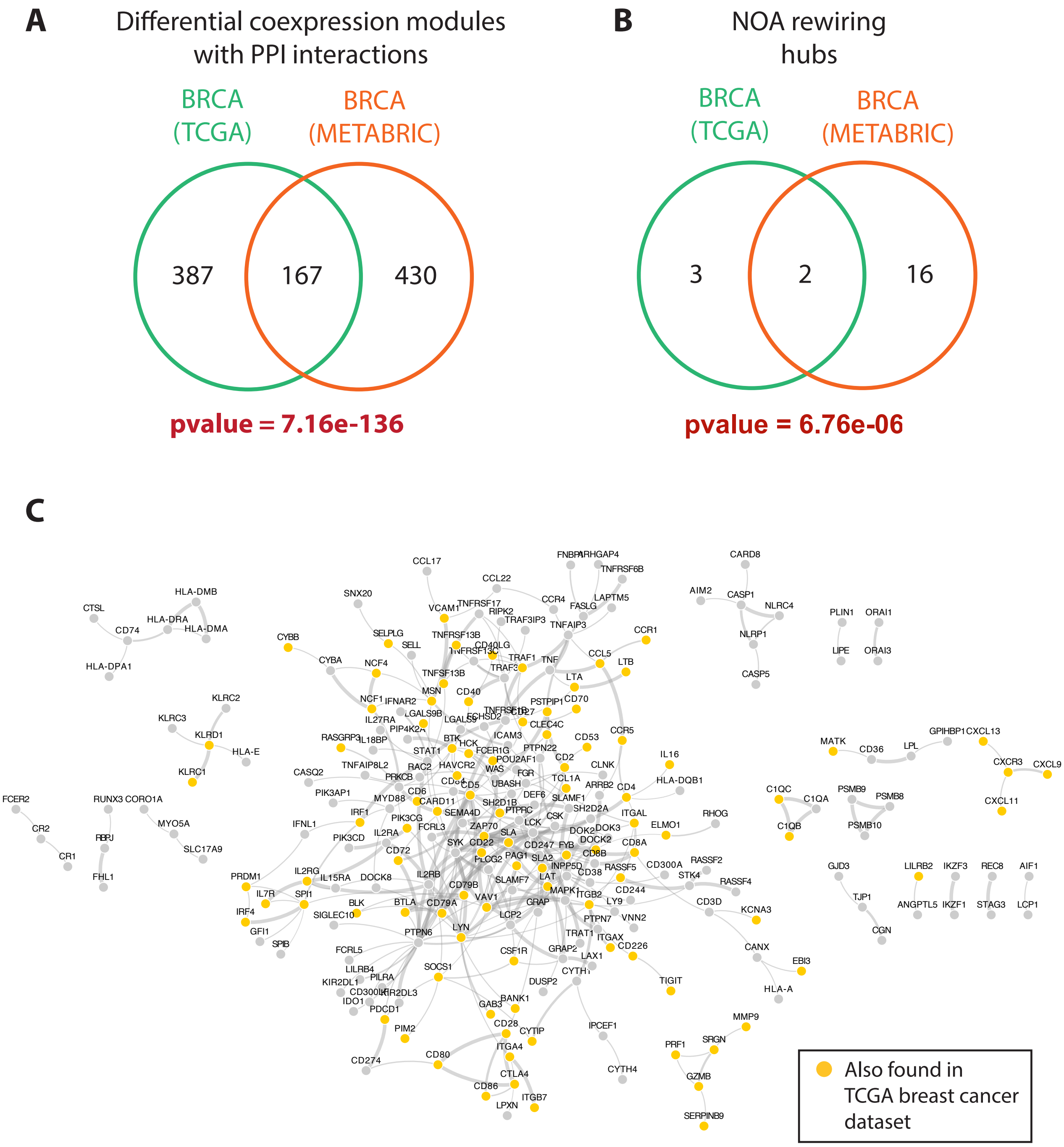

A. Validation of DiffCoEx breast cancer modules using the METABRIC discovery cohort. A. Output genes from the differential coexpression modules (overlapped with InWeb_IM interactions) were compared between the METABRIC cohort and the TCGA breast cancer showing an overlap of 167 genes with a significant overlap (pvalue=7.16e-136) calculated using phyper in R. B. Two NOA rewiring hubs (AURKB and CCNB1) were also found in the METABRIC cohort. C. The module with highest similarity to one of the TCGA breast cancer modules (module M4) are shown with yellow nodes as genes that have been found in both cohorts.

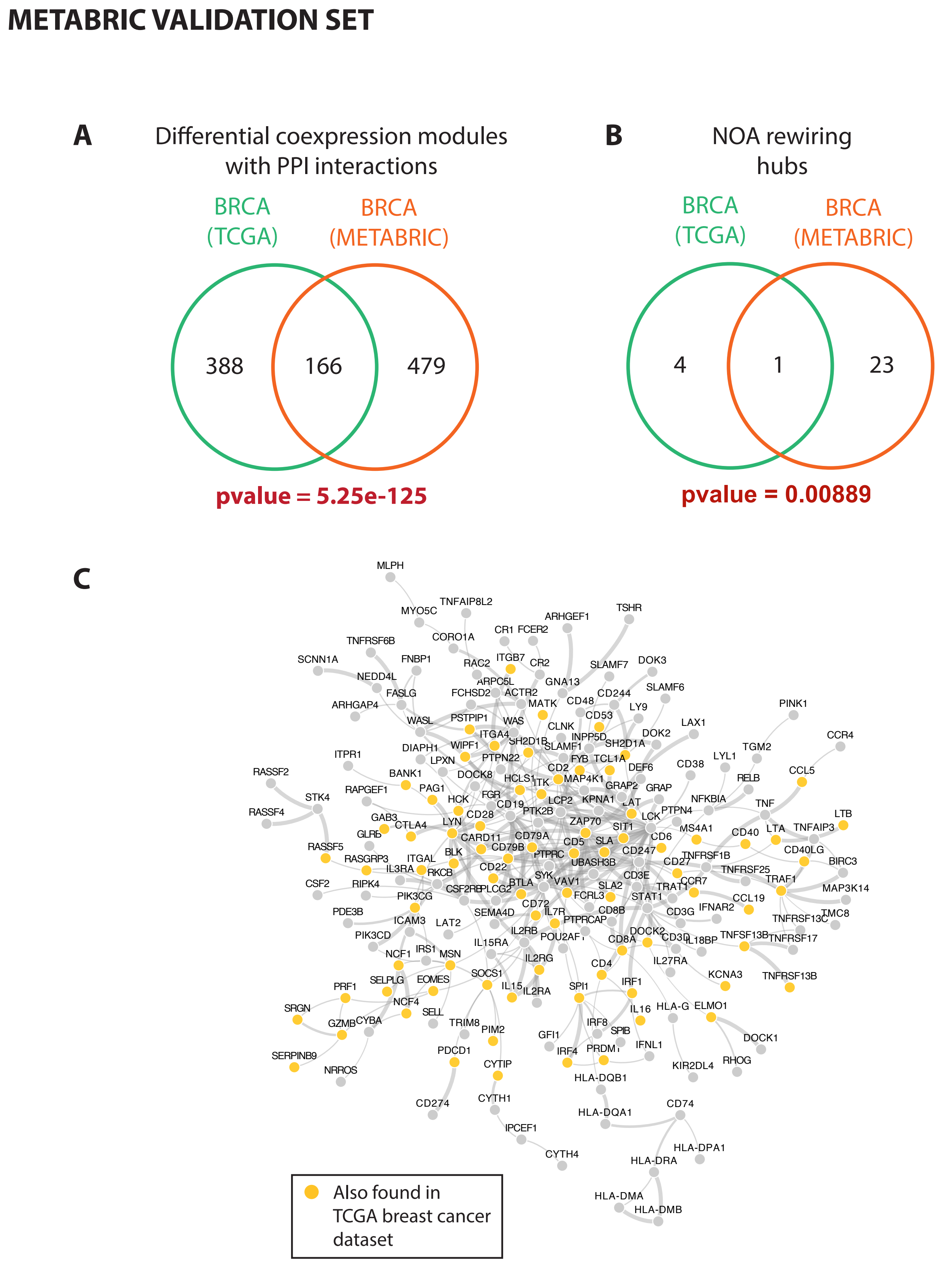

B. Validation of DiffCoEx breast cancer modules using the METABRIC validation cohort. A. Output genes from the differential coexpression modules (overlapped with InWeb_IM interactions) were compared between the METABRIC cohort and the TCGA breast cancer showing an overlap of 166 genes with a significant overlap (pvalue=5.25e-125) calculated using phyper in R. B. One NOA rewiring hub (CCNB1) were also found in the METABRIC cohort. C. The module with highest similarity to one of the TCGA breast cancer modules (module M4) are shown with yellow nodes as genes that have been found in both cohorts.

Figure S8

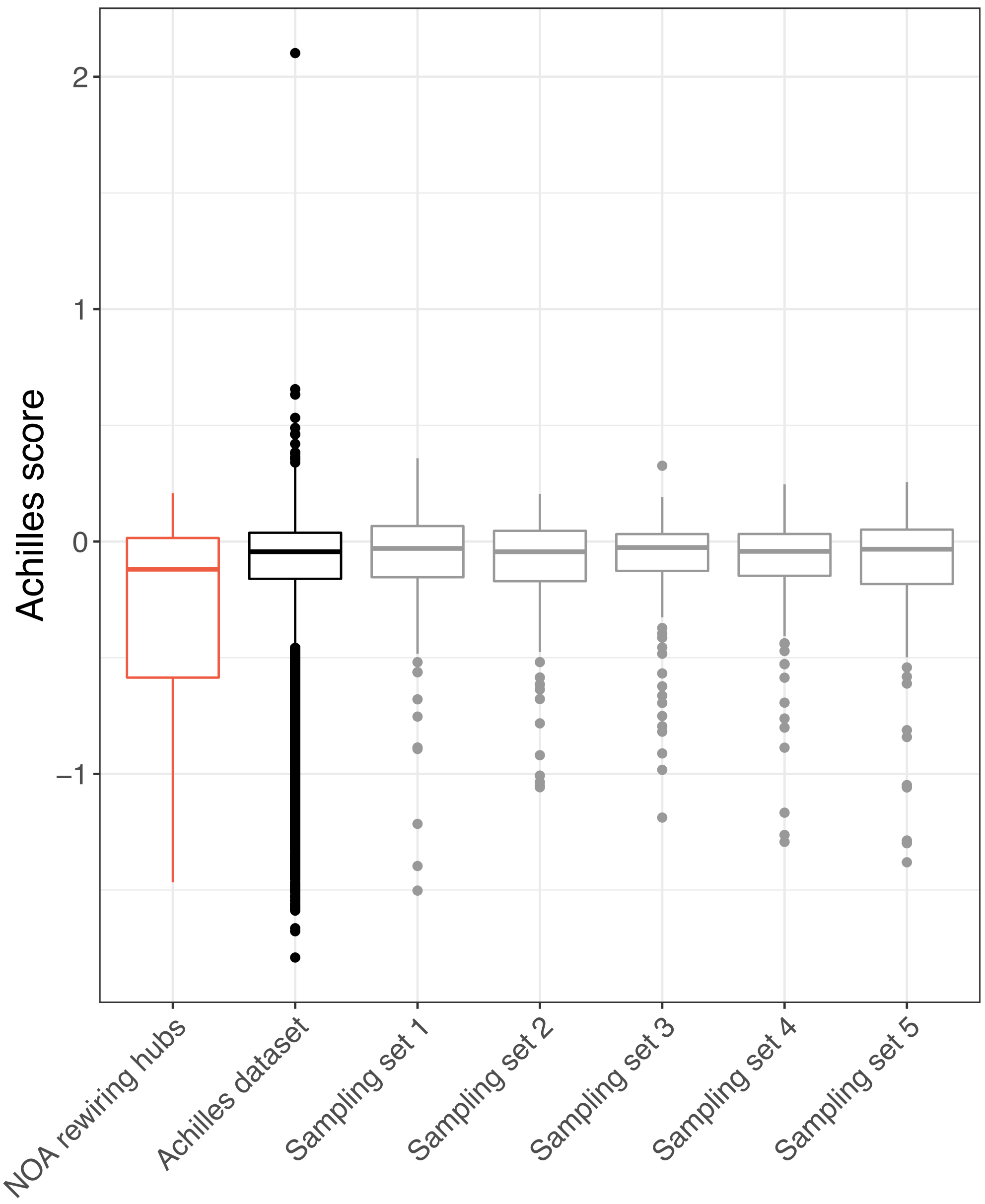

Achilles score distributions between gene sets: Achilles dataset (N=17,670, black), NOA hubs gene set (N=128, red) and sampling gene sets from the Achilles set (N=128, grey). The NOA hubs are significantly diverge from the average by pvalue=4.75e-05.

Supplementary tables

Table S1. Cancer name abbreviations

TCGA abbreviation	TCGA cancer type description	Abbreviations in this paper
LAML	Acute Myeloid Leukemia	AM leukemia
ACC	Adrenocortical carcinoma	Adenoid
BLCA	Bladder Urothelial Carcinoma	Bladder
LGG	Brain Lower Grade Glioma	Brain
BRCA	Breast invasive carcinoma	Breast
CESC	Cervical squamous cell carcinoma and endocervical adenocarcinoma	Cervix
CHOL	Cholangiocarcinoma	Cholangiocarcinoma
COAD	Colon adenocarcinoma	Colon
ESCA	Esophageal carcinoma	Esophageal
GBM	Glioblastoma multiforme	Glioblastoma
HNSC	Head and Neck squamous cell carcinoma	Head Neck
KICH	Kidney Chromophobe	Kidney-Chromo
KIRC	Kidney renal clear cell carcinoma	Kidney-Clear
KIRP	Kidney renal papillary cell carcinoma	Kidney-Papillary
LIHC	Liver hepatocellular carcinoma	Liver
LUAD	Lung adenocarcinoma	Lung-Adeno
LUSC	Lung squamous cell carcinoma	Lung-Squamous
DLBC	Lymphoid Neoplasm Diffuse Large B-cell Lymphoma	B-cell lymphoma
MESO	Mesothelioma	Mesotheliom
OV	Ovarian serous cystadenocarcinoma	Ovary
PAAD	Pancreatic adenocarcinoma	Pancreas
PCPG	Pheochromocytoma and Paraganglioma	Neuroendocrine
PRAD	Prostate adenocarcinoma	Prostate
READ	Rectum adenocarcinoma	Rectum
SARC	Sarcoma	Sarcoma
SKCM	Skin Cutaneous Melanoma	Skin
STAD	Stomach adenocarcinoma	Stomach
TGCT	Testicular Germ Cell Tumors	Testicle
THYM	Thymoma	Thymus
THCA	Thyroid carcinoma	Thyroid
UCS	Uterine Carcinosarcoma	Uterus-Sarcoma
UCEC	Uterine Corpus Endometrial Carcinoma	Uterus
UVM	Uveal Melanoma	Uveal

Table S2. **Significant differential coexpression modules (DiffCoEx) mapped to the protein-protein human interactome InWeb_IM**

Cancer type	Module name	Genes in module
BLCA	pink	HSPA1L, HSPA8, ALAS2, AR, ASS1, CDK6, DMBT1, GPC3, KCND3, MYH15, NLRP1, NOD2, NXF3, PRNP, THNSL2, TLX3, ZFYVE28
	turquoise	TAF1, CDC14A, MAT2A, NME2, HIST1H3A, PGAM4, ALDH1A3, CALML6, CHTF18, DST, H2AFJ, MTNR1B, MYO5A, OGDHL, TLE6, ACPP, ALDOC, ATL1, BNC1, CAP2, CLCN4, FRAT2, GAS2L3, GLUD2, LIMA1, LRIF1, MYO15A, NCOA2, PARD6A, PTGS2, RAB6C, RPL27, SDSL, SHBG, SLC12A2, STK17B, TRIM6, VDR, WASF3, WLS, ZNF860, ZNF98
	greenyellow	BSL1, ACTBL2, ACVR1C, GTSF1, HSPB1, IGF2BP3, MSTN, PPP1R10
	grey60	HSPA1A, DYX1C1, FBXO32, JUN, NFKBIA
	black	NTN3, KRT15, GJA1, TUBB4A, GNB3, COCH, ESR2, FLNA, ITGB4, LAMB1, MYO6, TCP11, TGM2, ACSL4, AMBP, ANTXR1, DNAH5, EFNB1, EPHA2, FHL3, FLNC, FOXS1, GFPT2, HOXD13, ITGA2B, ITGB3, KRT8, LAMB3, LYPD3, MSN, NAT8L, NEBL, NTRK1, PAK3, PLEKHG5, SALL4, SH3KBP1, SOX15, STX2, SYT1, TSPAN5, TUBB2B, ACAA2, ACOT9, ADAM19, AGR2, AMPH, AMY2A, ATG2A, BIN1, BLNK, BSPRY, CAMSAP3, CASZ1, CCNT2, CHN1, COL1A1, COL2A1, COL4A1, CPS1, CREB5, CXXC5, DAB2, DFNA5, DLG3, DRD2, ECM1, EGFLAM, ELOVL4, F2R, FBXL2, FERMT2, FES, FN1, GCGR, GJC1, GLI3, GNG2, GRIN2C, GRM8, HOXB13, IGF1, IGSF1, IKBIP, ISLR, ITGA4, ITGA6, ITGB5, KCNMA1, KRT19, KRT7, KRT81, LAD1, LAMA1, LAMC1, LAMP2, LOXL2, LRP11, MALL, MAP1LC3C, MAP3K3, MSR1, NCOR2, NLRP3, OGT, PDGFB, PLS1, PYCR1, RAB19, RARG, RASSF7, RRAS, SCN1B, SIRPA, SLC34A1, SPSB1, SULF1, SULF2, SYNJ2, TBC1D16, TEAD2, TFCP2L1, TINAGL1, TNFRSF21, TNFRSF25, TUBB2A, VPS13A, ZIC1, ZYX
	orange	HSPA1B, LGALS3BP, MDFI, SNCA, ACTG2, ARRB1, EPAS1, PIK3R3, ACTA2, APOB, CCDC8, LEP, LIN7A, MAPT, PGRMC1, PYGL, SLC9A3R2, SOCS2, TRIP6, APOA1, ARHGDIG, ARID5A, ARNT2, ARX, CD248, CHST8, CNR1, CPE, CRP, CTNNA3, CYP11A1, CYP2U1, DGKG, EDNRA, EPB41L2, EPB41L3, ERG, ESRRB, FBLN1, GAS6, GCK, GNA15, GPD1, GYG2, HOXA10, HTR2A, ISL1, KCNAB1, LEPR, LIMS2, LINGO1, MERTK, MGP, MOXD1, MYBPC2, MYH3, NDRG1, NEU4, NFATC1, NGFR, NOV, NTRK2, PDGFRB, PKP3, PLA2G2A, PLCB4, PLCG2, RBP7, RGS2, RGS7BP, RGS9, SLC2A3, SOX10, STXBP5L, TCF7L1, TFAP2A, TIE1, TRIM63, TSPAN2, WWTR1
	tan	CYP39A1, CLTCL1, HIST1H2BM, ATP1B1, BEX2, CCDC155, CYP4F22, EEF1A2, GYS2, C20orf195, CYP2C8, CYP2J2, CYP4B1, CYP4F12, CYP4X1, DNAJB13, DTX4, FOXD2, HOXC9, HOXD1, IGF2, IGFBP2, ISYNA1, MAGEA12, MAGEA4, MKRN3, NDRG2, RIPPLY2, SLC4A1, UPK2
	red	HIST1H2AH, TUBA3E, LGR4, ACTC1, ATP1A3, LRRK2, PGM5, ACTA1, ALDH2, ARHGEF6, ARPC1B, ATP4A, FBXO6, FGF12, GRIA2, MYH7, RAB27A, ADORA3, AICDA, ALDH1A1, ARHGAP4, ASGR1, BTK, BTNL8, CDC14B, CPVL, CXCR4, FAM46B, GALK1, HCLS1, HIST1H1B, HK3, IKZF1, KIAA0922, L3MBTL3, LAT, LCP1, LRRTM4, MAPK8IP2, MLPH, MYH6, MYO1F, PARVB, PDE4B, PGAP1, PIK3CD, PILRA, PLTP, PRKCQ, ROBO1, RPH3A, RSPO1,

		RSPO2, RYR1, SELL, SH2D2A, SNAP91, VAMP1, VCAM1, ZHX2
BRCA	blue	HS3ST4, SAA2, SAA1, SULT1E1, WIF1, KRT7, MEIS1, GAL3ST1, LAMB3, TNNT2, CASK, WNT8B, TNNI3, DCX, TUBB2B, LAMA1, ITGB4, CYP2A13, SMR3B, NLGN4X, MUC7, CHST9, NFASC, KCNN2, LAMC2, CHST3, ROR2, COL17A1, SFRP1, APBA1, COL7A1, CALML3, CYP8B1, GRIK2, DRD2, GRIK5, NRXN1, WNT4, KRT17, KRT15, KIF5A, HOXA9
	brown	MAGEA11, TNFRSF13B, BUB1B, PLK1, PLK4, SELPLG, LY96, TIGIT, EFNB1, CD226, IL27, ZXDA, PRAM1, CD40LG, CYP4F3, AXL, MS4A1, ASB2, CACNA1I, DLG3, SLA2, CYP2F1, TYROBP, FMNL3, FMNL1, ATP6AP1, CACNA1E, ATP6AP2, CDX1, STAP1, ESPL1, MAGI2, ARHGAP24, PIP, BANK1, MYB, PRTN3, FCAR, FMO3, CD28, ERBB4, CYP2C8, CAMK2D, CD22, CENPE, CD27, CAMK2B, PPP2R2B, SERPINB9, LTA, SIGLEC14, PRF1, LGALS9B, CXCL11, CXCL10, CXCL13, CYP2C18, SEMA7A, RASGRF1, MKI67, SOCS1, CD5, CXCL9, IL2RG, C1QC, CCR2, ACSL4, SH3KBP1, TNFSF13B, KCNV1, SERPING1, PLTP, TLR4, LAT, CD300C, CYTIP, GIMAP7, ELMO1, H2AFJ, CCL2, FOXP3, NFATC2, PDE6G, CCL7, CCL5, GRIA2, ACSL6, VSIG4, PPM1J, ITGA4, GAB3, C1QB, NOX1, TCL1A, EOMES, UTS2, HCST, ID1, Sep-01, CIITA, MFNG, SOX18, CYP7B1, NCF2, APOE, BMF, LILRB2, IL7, PRLR, OSCAR, KCNMA1, PPARGC1B, MND1, PIK3R5, TLR1, SYT16, JAK3, SYT14, HAVCR2, CCL21, CCL20, MSR1, CCL25, KCNIP1, EPHB2, CTLA4, IL5RA, ACVRL1, S100A8, ENG, CDC25C, PAX5, CHST8, STAT4, BLK, NPHS1, HCK, EFHC1, PTGIR, KIF15, TYRO3, ITGAX, IRF1, GZMB, BCL2A1, VAV1, MEP1A, MEF2C, IL12RB1, CD14, PDE6A, ITGAL, PRDM1, S100A4, CD80, FLNA, SIT1, TFDP3, MATK, BTK, IL7R, NCF1, CHRNB2, CD70, CD72, CCL3, SH2D1B, MYO1F, SH2D1A, CYP4B1, RET, NTRK1, CD4, CCR1, CD6, CCR3, CCR5, CD2, CCR7, SRGN, CCR9, PSTPIP1, PIK3CG, CD79B, KCNA3, CD79A, UTS2R, FCER1G, SPI1, GAS2L3, UGT2B15, ALCAM, BTLA, CSF1R, TMCC2, CD86, DOCK2, TNFSF9, S100A9, GIMAP2, C3, C2, CITED1, PTTG1, ICAM2, CHST11, KCNB2, CHST10, RASSF5, HMMR, CHST13, IL6R, CHRNA4, LTB, AGAP2, FOXL2, VCAM1, CHRNA2, CD200R1, CD8A, ABI3BP, WAS, LYN, LRP2, CCL11, SULT1C2, ITK, MSN, IL10, CCL19, ESR1, IL15, IL16, SECTM1, GDA, RAD51, PDCD1, STK39, EBI3, KIF20A, CD53, SPHK1, KLRD1, IL23A, PLAUR, PLXNC1, IL12B, BCL11B, NCF4, CYP17A1, HSPA1A, ALOX5AP, ITGAD, GHRL, TNFSF14, GAL3ST4, HIST1H2AC, XBP1, FYB, CEBPA, CCNA2, CEBPE, RASGRP3, FMO5, HS3ST3A1, CATSPER1, CXCR3, IFNG, PGR, ZAP70, AURKB, DOK5, HCLS1, ALOX5, PDZK1, KLRC1, TRAF1, KCND3, ITGB2, PIM2, ITGB7, CUBN, CARD11, SIRPB1, CD40, PRKCG, FCGR2A, CYBB, HES1, PRKCQ, PDCD1LG2, PAG1, SLA, CCNB3, CCNB2, CCNB1, KLRK1, ABI3, FCGR1A, CLEC4C, NOTCH1, IRF4, CD7, ABCC2, KCNA2, MMP9, C1S, WIPF1, ITGAM, CFP
	magenta	TNFSF15, SIGLEC12, TNFRSF6B, SIRPA
	black	SNAP25, CYP4F2, UGT2B11, CYP4Z1, CYP21A2, CDC25A, SCNN1G, SCNN1B, CPLX2, RPH3A, AR, HIST3H2A, DDC, UGT2B28, MAK, CYP4X1, PAK6, HIST1H3A, RREB1
	red	HIF3A, PTGS2, TIMP4, IL1RL1, PDGFRB, TIMP2, CD36, HBB, AKT3, JPH4, FBLN2, FBLN1, FBLN5, CAV1, DCN, LBP, ADAMTS1, PZP, RELN, IRAK2, IRAK3, CDH5, ADRA1A, TWIST1, LIPE, COL15A1, KCNAB1, AKAP6, KCNA1, KLK1, LPL, SNAI2, CXCL12, ADRA1D, CXCL1, BMP6, CXCL3, CXCL2, BMP2, AVPR2, VIM, AHR, ANGPTL4, ANGPTL1, SLC4A1, FIGF, NGF,

		HP, EPB42, ITGA5, HIF1A, DAB2, LAMB1, Sep-04, SERPINE1, IL1R1, RUNX2, CYP11A1, LRRK2, EGR2, EGR1, HOXA5, MEOX1, NID2, NID1, MEOX2, MAP1LC3C, ANGPT1, ANGPT4, IGF2, TCF4, MAP1B, IGF1, SULT1C4, HIST1H2BD, IGFBP3, GYS2, IGFBP6, IGFBP7, PLIN1, F10, LAMC1, FGF2, CHST7, PRDM16, PPARG, CYP19A1, ANPEP, TRPC6, SNCA, NOV, TIE1, MYH8, EFNB3, PDGFRA, SMAD9, DTX1, MYO1E, TRPC3, TRPC1, AKAP12, ADCYAP1, GRASP, KCNA5, A2M, AKR1C2, AKR1C3, TLL1, TEK, SNCAIP, S1PR1, FHL1, TFPI, C7, C6, KDR, FGFBP2, TGFBR2, LAMA2, CFHR1, ELN, NDN, CYP1A1, LRP1, COL18A1, SGK2, PAPPA2, ID3, KL, LEP, CTGF, PDE4D, PRKD1, NGFR, HS3ST2, PLA2G4A, ADAM12, GPD1, BMPER, HIST2H2BE, SEMA3D, TAL1, LOX, ADCYAP1R1, GYG2, TRDN, CASQ2, CLEC3B, NAP1L2, TWIST2, CLIP3, KLF2, MYH1, JAG1, DPP4, GREM2, PGF, ITGB3, NOS3, PRKCA, GPC3, IL33, TNC, EPHA10, HGF, CYTH3, NRP1, FLNC, MAGEH1, NOTCH4, C1QTNF1, CA4, DYNC1I1, ANK2, PTPRB, PLVAP, PTRF, MMP2, MMP3
CESC	lightsteelblue1	NXF1, TAF1, ZNF480, ZNF689, POLA1, THOC2
	turquoise	AR, BICD1, PKLR, SLC25A41, TUBB2B, ALDH2, ATP6V0D2, CDC42EP3, ENO2, GHR, LYL1, MUCL1, PRKACB, PRKCQ, PTPN12, ABI3, ARHGEF6, BCL11B, C10orf35, CACNA1I, CATSPER1, CCL23, CD3E, CDX1, CYP3A5, DAB2, DLG3, ECM1, FCN1, GRIN2A, HEY1, HSD11B1, HVCN1, IL16, KCNA5, KCND2, KIF21B, KLC3, KLK8, LMO1, LRRK1, LRRK2, MATK, MMRN1, MYBPC3, PAG1, PGLYRP2, PRKAR2B, PTPN22, RAB39A, RFTN1, RGS1, RGS18, RGS5, RUNX2, SFRP4, SH2D1B, STK39, STX1B, TBX3, TLR4, TNFSF9, VAV2, ZBTB32, ZNF606
	darkgreen	HIST2H2AC, CEBPA, ADRB2, OFD1, ZNF267, AKAP5, CEACAM1, CPA4, ENDOG, FOXP3, IL32, MYL6B, NTRK1, PARP10, SDS, SEPT12, TXLNG, UTP20, ZNF583, ZNF649
	saddlebrown	HSPA5, CLTCL1, PRKCE, SNCA, GFPT2, HIST1H3A, PIK3R1, CDC14A, GFPT1, PTCH1, TUBA4A, AP2B1, EIF5A2, EPHA4, GYS2, PIK3R3, PRDX4, PRNP, TMOD1, TUBB6, ACTA1, ADAMTSL4, ALPP, CACNA2D1, CAV1, CBS, CYP11A1, CYP2S1, FOXB1, FOXL1, GRK5, HECW2, HIST1H1B, HMOX1, IGFBP1, MAP7D1, MICALL1, MSN, NEK7, OAT, PDLIM7, RGN, SATB1, STX1A, TF, TSPAN3, WIPF1, ABCG5, ACVR1B, AIM2, AKR1B1, ALKBH3, ALS2CR11, AP3B2, ASB9, ATF7IP, ATP6V0A4, ATP8B4, BATF3, CAND2, CCL2, CCNJL, CD40, CDC14B, CLIC5, CLTB, CNNM1, COBL, CRYAB, CXXC5, CYP27C1, DUSP9, EFNB1, FBXO44, FGF1, FGFR3, FOXA2, FOXE1, FOXP2, FST, FZD8, GAD1, GATSL3, GCNT1, GLI3, GPHA2, H1F0, HCK, HLF, HOXA1, HOXA6, HTR7, IGFBP3, INHBE, JUP, KRT13, MAFB, MAPK10, MPP3, MSX1, NCAM2, NCK2, NEXN, NGB, NOMO2, NRG1, OLFM4, OSBPL1A, OSCP1, OTUD7A, PLK2, PLS3, PLXNB3, PNCK, PNPLA3, PPP2R2B, PPP4R4, PRKAB1, PTRH1, RGS2, RIN1, RRAGC, S100A10, S100A13, SCML2, SCPEP1, SLC27A2, SNCG, SOCS3, SP4, SPINK2, SPOCK2, ST8SIA4, STRBP, TDO2, TG, TGM1, TMED4, TNNC1, TOX3, TRIM15, TRIM29, TRIM69, TSC22D3, TSPAN2, TWF1, USP54, YPEL1, ZC2HC1A, ZDHHC15, ZIC2, ZNF711
	darkturquoise	H2AFJ, ZNF674, AFP, PPP2R5B, ZNF233, ZNF563
	yellow	CCDC8, CEACAM5, GK, MYH14, ATP2A3, C3, CD209, CHGB, CPM, CXCL5, CYP4F2, CYP4X1, DFNB31, ETV5, FCGR3A, GPD1, HAVCR2, IKBKE, IL27, IL6R, ISYNA1, ITGA7, LIMCH1, LRP4, MGLL, MMP9, MYH10, MYO18A, NPHP1, PGAM2, SGK3, SLC22A2, SLC25A5, SLC3A2, SLN, SUSD4, TNFRSF11A, TNFRSF11B, TRPM6, VSIG4, WIF1, WNT3A
	yellowgreen	AK5, CADPS2, CPNE6, DMD

	black	HNF4A, EEF1A2, GAPDHS, ESR1, ESR2, VPS13C, HSPA6, TUBA8, ATP6V0A1, GTF2H3, PLCB1, CCNA1, DNAJB13, GNAI1, IVNS1ABP, LMO4, MTNR1A, NME3, NPEPPS, PDE4D, PDZK1, PYGM, ACTR1B, ALDH1A1, ALDH1A3, ATP2B2, CALML3, CALML5, CCNT1, CTSE, CYB5A, GRIN1, KRT15, LRIF1, MAPK11, MCC, NEBL, NPC1, PGAM4, PRKCA, RBPMS, REN, SOST, SPTBN2, TREH, ACTN3, AIFM1, AKR1B10, ALPPL2, ANPEP, ARL3, ATP2A2, AUTS2, BPGM, CDK20, CDKN1A, CITED1, CITED4, CNGB1, CYP21A2, CYP24A1, DAPK1, DHX9, DLGAP1, DUSP19, ESRRG, EVI5, GATA4, GNAZ, GPX2, GRB10, GRB14, H3F3C, HAL, HDAC9, HIPK3, HNF1A, HNRNPA2B1, IER5, INSR, ITGA6, ITSN1, JSRP1, KRT16, KRT34, KRT81, KRT85, NAPSA, NETO1, NFE2L1, NR1D1, NTHL1, PCSK4, PFKFB2, PHLDB2, PIWIL1, PLEKHG2, PRICKLE4, PRKX, PRSS50, RNFT2, SERPINC1, SERPINE1, SHANK2, SLC18A2, SLC34A3, SLC9A3R2, SMARCD3, SPERT, SSR4, SUOX, SYCE1, TDGF1, TDRD1, TFAP2A, TH, TRIM55, TUBA1C, XDH, XIRP1, ZNF488, ZNF608, ZNF860
	darkred	HIST1H2BL, DNAJB5, GTF2H2
	orange	ACTA2, NME2, PRKAA2, CASK, GCK, HIST1H2AH, PARK2, FOS, LIMA1, PABPC5, PGM1, PLS1, TRIM9, TUBAL3, DDX3X, HIST1H2AB, KCNMA1, MAP1B, ZNF311, CACNA1A, CBLB, CTH, DLG4, FEZ1, FHL2, HIST1H2BO, KRT8, MYH11, MYH15, PPP1R9B, UBQLN2, VIM, ZNF605, ALDH1A2, DTX1, EPAS1, ERRFI1, GJA1, KIF5A, LIPH, MMP3, PPARGC1A, RBBP5, RRM2, TRIM63, ACOT9, ACTN2, ALK, APOA1, BASP1, CADM1, CALD1, CAMK1G, CHST10, CLU, ERG, F2, FBP2, GNAT2, HEYL, HPX, HS3ST6, HSD17B10, KIF4A, L1CAM, LAMA1, LGALS3, MAP1LC3C, MAPRE2, MEF2C, MSH4, MSTN, MYH6, MYH7B, MYOCD, NAP1L2, NOVA1, NRIP1, OGT, PBX3, PCSK5, PTCH2, PTH1R, RGPD1, RGS4, RPS6KA3, SGPP1, SMAD6, THNSL2, TNFSF11, TNNT1, ZFP37, ABCB4, ABCB7, ABCC4, ACAA2, ACSL4, ACTBL2, ACVRL1, ADAMTS1, ADAMTS2, ADCY3, AGTR1, ALAS2, AMOT, ANK2, AP1S2, APBB1, APPL2, ASS1, CA14, CACNB2, CASQ2, CD93, CDH13, CDH2, CHFR, CHST13, CHST9, CKMT2, CLDN14, CLDN16, CNTFR, CNTN2, CPS1, CRTAC1, CYB5R1, CYFIP2, CYP17A1, CYP39A1, DTX3, DYSF, EGR2, EVL, F12, F2RL2, FADS1, FAM13C, FAM46A, FES, FHL1, FLNC, FLT4, FSTL3, GAB2, GDF11, GDF9, GJA5, GNGT1, GRASP, HDAC7, HGF, HIST1H1D, HTR2A, IGF1, IGFBP6, IGSF1, IL6ST, IRAK3, JPH1, KALRN, KCNK1, KCTD17, KIF14, KIF4B, KIF5C, KRT17, L1TD1, L3MBTL3, LAMB3, LCA5, LDB2, LDOC1, LHX1, LHX6, LIN7A, LMO2, LRRC8C, LYN, MAGEA8, MAP9, MED14, MET, MMP7, MYH13, MYL7, MYLK, MYO16, MYOM2, NACAD, NCOA7, NDN, NEFM, NELL1, NES, NQO1, NR4A2, NRXN2, NTRK3, NYAP1, OLFML3, PAK3, PAM16, PDGFRA, PDGFRB, PIWIL2, PJA1, PLVAP, POPDC2, PPFIA2, PTPRT, RAB3IL1, RAI2, RALGPS1, RAPGEF4, RBM15, RECK, RGS16, RHOC, RPGRIP1, RTN2, SAT1, SCAMP5, SCN8A, SDCBP2, SH2D3C, SIRPA, SIRT4, SLC25A34, SLC43A1, SNAP25, SORBS1, SOX10, SULT1C4, SYT1, TAGLN, TANK, TCEA2, TCF21, TIMP4, TOB1, TRIM46, TRO, TRPV4, TWIST1, UBE2A, ULBP2, VAT1, WASF3, WDR45, ZMYND8, ZNF154, ZNF431, ZNF454, ZNF462, ZNF626
	tan	HDAC5, ALPL, NTN3, PPARG, CBX5, CREB5, HNRNPH2, JAZF1, MDFI, NTN1, PRDM16, RIMBP3, RPS8, SLC9A1, TAF7, TTLL6, USP51
	plum1	UBC, CORO1C, FBXO6, FMNL2, NOS2, PLOD1, STK32C, TLX3, TTN, ANKRD1, FOXN1, GFAP, GLUD2, HYAL1, ITGB4, KRT19, KRT2, LCK, MAPK12, MYO1D, NFKBIA, PLCXD2, PLEKHA7,

		POF1B, PRAF2, PTPRZ1, RAB3B, RARB, RREB1, SATB2, SCARB1, SP1, SPRY2, STEAP4, TFAP2C, TLE1, TPM2, UBTD1, ZNF423
	darkorange2	AGPAT2, SLC27A6, TUBB2A
COAD	blue	XPO1, PPP2R2B, HSPA6, STAT2, AKR1B10, APPL2, ATP1A3, ATP6AP2, BICD2, DBN1, GZMB, HS3ST2, IL12RB1, IL23R, MAPK11, MAPK12, MAPK8IP2, MITF, NCS1, OCRL, PPM1J, PPP1R9A, PRKCDBP, PRPS1, PSMB10, PSMB8, SDS, SULT1C3, TIAM1, UPRT
	brown	DKK1, IGF2BP1, KCNMA1, BEX2, BMP6, CKM, COCH, DPP4, GABRA3, GABRB2, GNAO1, GOLM1, HFE, KCNA6, LTF, MKRN3, NGF, NTS, PKHD1L1, PRSS3, RGS20, SOSTDC1, TFRC, TP63, WNT3A
	turquoise	JUN, ACSL4, ENO2, HNF1A, DEC1, OAT, RORC, STAT1, ACSL5, ACSL6, AKR1C3, BCL6, CAMK1D, CRY1, DUOX2, GAL3ST2, HMGCS2, HS6ST2, L1TD1, PRKAB1, RPL10L, SGTB, UGT1A8, UGT2B15, USP13, ZNF267
	yellow	ZNF468, ACVRL1, ATP2B2, CADPS, CALCA, CALML3, DMD, ELF5, EMX2, HIST1H1D, ID3, MLH1, MSH4, NAV2, PRPF40B, SCTR, TGFBR2, ZNF470, ZNF606
	black	ECT2, HOXB9, NR0B2, RARG
	magenta	EMX1, CBX2, ELK1, SCML1, SPDEF
	red	EIF5A2, GCK, HIST1H2AH, HIST1H2BL, RAB30
HNSC	salmon	UBC, SCN8A, TCEAL3, TCEAL8, TWIST1
	black	EVL, LYN, PLCG2, ASGR2, CCL5, CHN2, CST9, EFNA5, FYN, ITGA4, NOX5, RAC2, SOAT1, STAT1, WT1
	orange	CDX1, ESR1, COL9A1, SH3GL2, SREBF1, TGFBR3, C4BPA, CORO2B, DLG4, FN1, KIF1A, NGFR, NOS3, PGAM4, PRKCA, PTCH2, SCIN, SGK3, ACTA2, APOD, AR, AVPR2, AXIN2, CAPN9, CCDC8, CNR1, COL4A2, COL5A1, COL6A1, COL6A2, CTNND2, CYP1B1, CYP4F2, DACT1, DDAH2, DDX43, DYSF, EFNA2, EPB41L3, EPHB2, ERG, F13A1, FGF1, FOLR1, FUS, FXYD6, FZD8, GRM7, HDAC9, HDC, HEY2, HMGCS2, HNRNPA2B1, HTRA1, IRAK3, KCNMA1, LEF1, LINGO1, LIX1L, LRRC7, MAGEH1, MAOB, NKD2, NOV, NOVA1, NR2F1, NRP1, NTN1, NTN3, PAPLN, PDZD2, PTPRN, PTPRT, RAB3B, RIMS1, RPL22L1, RUNX2, SPOCK1, TGFB2, ZNF175, ZNF221, ZNF347, ZNF429, ZNF549, ZNF611, ZNF614, ZNF616, ZNF689, ZNF91
	green	HSPA1A, PRKX, ABHD1, NHLRC1, SMC1B
	magenta	SLC25A4, CAP2, SLC2A4, ACSF2, ACTA1, ACTC1, CMTM5, DPF3, EEF1A2, GADD45G, HP, JPH2, MYOC, SMARCD3, TPM1, TUBA8, YPEL1, ZIC2, ZIC5
	tan	CBS, GYS2, ADAMTSL4, ALOX12, CCP110, DNAJB13, ELOVL4, FGF22, FGFBP1, LCE1B, LMNA, LYPD6, PTPN18, STX19, SULT1A2, TCEA2, TEC, TUBB4A
KIRC	blue	NTRK1, ADRB2, CBS, HIST1H2BH, ITGA4, ANKRD44, ARRB2, ATP8B4, GCH1, ITGB7, LCP1, PRDX2, RNF11, STIP1, APBA2, ARHGAP11A, BUB1B, CD68, CDC20, CDK1, CDK5R1, CDT1, CIITA, CPVL, EPB41L1, EPB41L3, FAM46C, GTSE1, HDAC11, HIST1H1D, HMMR, IGF1, IGF1R, IRF6, IRF8, LAP3, LMNB1, LYN, LYZ, MAD1L1, MAP3K8, MUL1, OCRL, PLK1, PLK4, PTH1R, PTPRC, RGS10, SFN, SLC16A2, SRM, STAT1, STAT4, TOP2A, TRAF1, TRIM69, UBASH3A, UBE2L6, WARS, WDR76, ZFP1, ZNF267
	brown	DLX5, PKN3, TGFBR2, AKT3, COL6A1, CSNK1E, FOXI2, FOXO1, FRZB, GAB1, HDAC7, HES1, MEF2A, PDGFB, PLEKHH3, PTPRJ, RARB, RARG, RASAL2, SMAD1, TLE1
	turquoise	PGD, PYGL, TUBB1, LIG4, OGDHL, PLS1, AKR1B10, CAMK1,

		DNAJB13, PNPLA2, PRKAB1, RAB3D, SF1, TUBA3E, ALDOC, AP2A1, C4BPA, CASZ1, CYP2C9, FKBP5, GJB2, HPD, ITPR3, MAT1A, NUAK2, OXCT1, PAPLN, PIEZO1, PROS1, REPS2, SCD5, SERPINF1, SOD2, SOWAHC, STK32A, SUZ12, TGM3, TRPC7, WT1, ZNF433, ZNF460
	greenyellow	AXL, FLNA, GAS6, LEF1, MYH11, NRARP, PDLIM7, PLCG2, RGS6, SDHB, SLC16A5, SPECC1, STMN2, SYT11, VCL
	grey60	ALDH2, ETV1, ETV4, HIST1H2BL, HIST1H2BN
	pink	AP1B1, CEBPE, CHEK2, JUP, MAPK3, PSAT1, RPS6KA4, WWTR1
	black	CYP19A1, CYP26B1, DYNC1I1, NTN3
	tan	DAB1, AKR1B1, APLP2, ISYNA1, MBNL3
	red	AKR1D1, ALDH1A3, GCK, CHN2, CHRM4, ERBB3, HIST1H1C, MYO1E, OSBPL6, PITPNM1, PPP2R2B, PRKAA2, PSME4, SH3GL3, TRIM29
KIRP	pink	LDHA, ATP6V1A, CLTC, MCM2, OGDHL, PSMD11, ACSS1, ANXA2, ATG4D, ATP8B2, B3GALNT1, CST6, EGLN2, EGLN3, KCNJ11, LDHB, MCOLN3, MUC16, NOP14, RAB5C, SLC25A5, SUPT5H, SYNGR3, TLE1
	blue	IFI16, RCC1, ACTB, HSPH1, KYNU, LGR4, PAK3, RAB8B, RCN2, SEC23A, SEC23B, TRIM54, AIM2, ANTXR2, ARHGAP25, CASC5, CD27, CDK18, CDT1, CEBPZ, EFNB1, FGFR2, FGR, GNB5, IFNG, INPP5D, ITGA5, KCNMA1, KHSRP, KRR1, NUDT21, PAPOLA, PLK2, PML, POLD1, PPP1R18, PRF1, PRKAR1A, RAD50, SMC3, TAP1, TOP1, TOPORS, TSPAN5, TTYH3, TUBGCP4, TUBGCP5
	turquoise	DNAJA1, HSPA1L, IKBKG, FYN, UBD, FN1, ACTG2, HSPA5, RBBP7, E2F1, COPB1, HSP90B1, CALML3, HIST1H2BK, MAT1A, NOP58, ADRB2, ASF1B, CDC27, DNAJB1, IARS, PCK1, PLK1, PPARGC1A, PRKDC, RRM2, SKP2, BRCA1, CENPA, DNAJA4, FLNA, HNRNPK, KDM5C, KHDRBS1, KIF23, LAMA1, NUP62, POLR3A, PRDX4, PRPS2, RPL10, RPL3, TAF5, WDHD1, ANLN, BOP1, CCNB2, CCT4, CKAP5, CKS2, DDB1, FASN, GSPT2, HDAC6, HNRNPC, LMNB1, MBNL1, MYH4, MYH8, NAP1L1, NR4A1, PRKCSH, RAD23B, SHMT2, SPTAN1, SSRP1, TREH, UCHL1, AATF, ASAP1, BCAR3, BMPR1B, CCNE1, CDC42BPA, CHAF1A, CHEK1, DLG2, FAM46C, FOXM1, G6PD, HJURP, HNRNPL, IGBP1, INCENP, KRT20, MARCH6, MCM4, MED12, NUP210, PER1, PPP2R2C, PTN, PTTG1, PYCR1, RAD51, RFC3, RYR2, SALL4, SCD5, SKA3, TERT, TFAP4, TNPO1, TPX2, TRIM55, UBE2NL, ABCC1, APLP1, ATAD2, ATG16L2, ATIC, ATP1B3, AZGP1, BEND3, BIRC5, BZRAP1, BZW2, C15orf41, C19orf57, CCNA2, CCNJL, CDC45, CDK1, CEACAM5, CHD4, CHN1, CMA1, COPS4, CXorf57, DKC1, DLGAP2, E2F2, EHD1, EMX1, ESPL1, F2RL2, FAM64A, FZD7, GLDC, GTSE1, H2AFZ, HS6ST2, ID3, IFIT3, IPO7, ITIH4, KIF11, KIF20A, LHB, LHX6, LIG3, LRRC59, LSM12, LURAP1, MAP2, MCM10, MCM6, METAP1, METAP2, MKI67, MPHOSPH9, MYLK2, NAT10, NCAM1, NCAPG, NID1, NLRP2, NMD3, NOLC1, NONO, NR1I2, NUP155, PAICS, PGBD1, PLEKHB1, POC1A, POLR3G, PPAT, PPP1R3D, PRC1, PRIM1, PTPRD, PTPRS, RACGAP1, RBBP5, RIPK2, RPL7, SGOL2, SH3GL1, SLC25A34, SLC39A4, SLITRK5, SQLE, STC2, STIP1, STON2, TAF1D, TCP11L1, TK1, TOP2A, TP53INP1, TRAF5, UGDH, UGGT1, UPF3B, UTP18, WDR76, YY2, ZNF207
	darkgreen	ATP6V1B1, MYO5A, NTN1, VCAM1, EEF1B2, PPP2R2B, AKT3, GFPT1, HNRNPH1, HNRNPM, PRKCE, SGK1, U2AF2, BAG3, FAS, KRT1, MAPK11, MYH1, MYH7, NXF1, PDE4D, PRKACB, SFPQ, SPTBN1, TRIM28, ZNF214, ZNF250, ZNF676, AIFM3, ALDH1A3, ARG2, C4orf22, CASK, CBR3, CCDC39, CEP152, COL5A1, CTSD, DMWD, EFNA2, EPHA7, ESRRB, ESYT1, GARS,

		MAG, MAPK15, MED25, NAPSA, NOTCH2, PALMD, PDE3A, PRSS3, RHOBTB3, RPLP0, SCGN, SDC1, SPTLC3, SYP, TFCP2L1, TFPI, USP12, ZNF98
	brown	MAP1LC3A, HNRNPA1, ITGA4, DSP, RPS2, AP2S1, AZIN1, BCLAF1, CCNT1, CDKN2AIP, EIF4EBP1, OAZ1, PCOLCE2, PDPN, PGAP1, RAB33B, RBBP6, RPS14, SCD, SEC11C, SLC6A7, SREK1, SSR4, STRN, UBA52, ZNF175
	lightyellow	UBC, SSX2IP, ATP6V0D2, GYS2, LIMA1, TP63, XRCC1, ARPC5L, ARRB1, ATP2A3, BEX2, C14orf105, CEP89, CYLD, ELAVL3, EPAS1, FCN1, FSD1, KIT, KRT14, LMO2, NAV1, NEXN, PHLDB1, PLEKHG1, POPDC2, RGS9, SAFB, TEX11, ACTA2, ACTC1, ADCY3, BATF3, BBS1, BTG2, BTN3A1, BTN3A3, CCDC36, CDC34, CEBPB, CELF2, COL12A1, COL5A3, COX20, CSPG4, CYP4X1, CYTH1, DUSP1, EHD4, ELN, FAM124B, FATE1, FAXC, FILIP1, FOXI2, GAS1, GFI1B, GFPT2, GJB1, GJC2, GLI1, GPR3, GRIP1, HEYL, HMCN1, HOMER2, HPS5, IGF1, IGFBP3, IL33, INPP4B, ISLR, JPH4, JUNB, KMO, LRFN3, LY75, MATN3, MICAL2, MYEF2, MYH11, MYOCD, NPR1, PDE4A, PDE4C, PKN3, PLA2G16, PLCG2, PLN, PLXNA1, PPIP5K1, PPP1R3B, PRND, PRPS1L1, PTK2B, PTPN5, RAB31, RAI1, RASSF1, RPS6KB2, RUNX1T1, SCN10A, SEC61A1, SH3KBP1, SH3PXD2A, SIPA1L2, SLC14A1, SLC1A2, SLC27A1, SLC2A14, SLC8A1, SLC9A4, SLN, SMYD2, SNRPA, ST6GALNAC1, STMN2, TACR1, TAL1, TFDP2, TGFB1I1, TGFB2, TLR4, TMEM110, TPM2, TRERF1, TSC22D2, TSHR, TSPYL2, VSNL1, ZNF782, ZNF823
	darkred	HSPA1B, ACLY, ASL, HDAC5, IRAK1, NME2, STIL, AEN, ATP10D, ATP1A3, BCAT2, CACNB4, CEP85, COMTD1, DYRK1B, ETFB, FARSA, GLCCI1, HEATR3, IDH2, LONP1, MAK, MAPK12, MRPS12, MRPS16, NDUFB7, P4HA1, PAPOLG, PDHA1, PRDX5, REEP6, SAMM50, SASH1, SLC27A4, SPHK2, TUBB2B, UBL4A
	green	HNF4A, HOXD8, TLE6, HOXB8, HSPA2, DLG4, MYC, CFTR, TUBB4A, ERBB4, LRIF1, NRG1, ZFP37, ZNF331, DSTN, FKBP5, ZNF154, ZNF470, ZNF549, ZNF613, ZNF649, ZNF665, ZNF677, ZNF829, AHSA1, AR, CDC20, CHD3, DNM3, EPHX1, FOXD4, NIPSNAP1, NSDHL, PALM, PDZD3, PIK3R2, PSMC4, RABGGTB, RRM2B, TBC1D17, TFAP2A, ZNF257, ZNF439, ZNF471, ZNF534, ABCC2, AKR1B15, AKR1C3, ANKRD1, AP1M1, ARHGAP18, ARMC6, ARNT2, ASNA1, ATP1B2, C8G, CACNG4, CAMK2N1, CCDC8, CCNG2, CDKN2C, CYB5R2, CYP27A1, CYP4F2, DAB2, DDX58, DMKN, EPN3, EPS15L1, ERCC2, FEZ1, FMNL2, FTH1, GALNTL6, GNB1L, GRIK5, HK1, HNRNPF, HOXB5, HOXD4, HS6ST1, HTR2A, KDELC1, KIF5A, LETM2, LRP1B, LYPD3, MGST1, MPP6, MSX2, MUC1, MYO5C, MYO6, OCRL, OSR1, PC, PDZK1, PIAS3, PIK3AP1, PIPOX, PIR, PNMA2, POP1, PORCN, PTBP3, PTCH1, PTK6, PTP4A2, RAB10, RAB3B, RIMBP3, RNH1, SDCBP2, SERPINA10, SH3BP4, SH3YL1, SIX4, SLC22A18, SORL1, SPATC1, SPRY2, SREK1IP1, STAP2, STX3, TDGF1, THNSL2, TINAG, TKT, TLE2, TLE3, TM7SF2, TMEM161A, TMEM216, TMEM45B, TRIM6, TXNRD1, USP46, VCL, WNT4, ZBTB32, ZFP28, ZNF219, ZNF577, ZNF667
	yellow	EIF3C, ESR1, HELLS, TEAD2, BARX2, CHD1, CTPS2, DEK, DNMT3L, EIF5A2, GPD1, HOXB13, MAP3K1, MAP4K2, POU2F1, PRDM5, PRKX, ZNF641
	orange	ASAH1, ATP1B1, CAMK1D, CRIP2, HIST3H2A, HMGA2, ITGA6, LAMC2, RARRES3, TGM1
	black	PRKAA2, C4orf26, CYP3A43, FHL2, ITGB5, LYN, MAPK13, MDFI, NDN, PGRMC1, RBM45, RNF180, RNLS, SP2, STAT3
LGG	turquoise	MET, INPP5D, STAT3, CASK, CD4, CD44, CD8A, FAS, HCK, IFI30, IL18, ITGB4, MUC1, PLCG2, PTPRC, RNLS, SEPT6, SLA2,

		SYK
	darkgreen	LRRK2, PRKACA, NEDD4, DLG4, FLNB, GFPT2, HNF4A, UBB, FLNA, MYO5B, NTN1, HSPD1, NOTCH2NL, PRKACB, RPA1, EEF1A2, GABARAPL1, MYO5A, PIN1, TMEM17, TMEM216, MAP1LC3A, PNCK, ACTC1, DHRS11, HUWE1, LIN7A, SYNPO, ULK2, ATP6V1B2, CHD3, DNAJA4, EIF5A2, PGK1, RPS9, YWHAG, AKR1B15, ATP6V1A, BCL6, HMGCR, MYH13, PRKAR2B, PRKX, PRPF40A, TJP2, TUBA4A, UBASH3B, YWHAB, ZDHHC13, ALDH1A3, ALDH3A2, ARRB1, CAP2, FOXD4L6, HK1, HNRNPF, IFI16, NAP1L2, NEK7, PPFIBP1, PTK2B, RAB13, ROBO2, STX1B, TLE3, UNC119, ABCA1, ACTR1B, CAD, CAMK1G, CBLB, CORO2A, CYP2C8, DCTN1, ECI1, FOXD4L3, GNA13, GNB5, IMMT, IRS4, IVNS1ABP, MAP1B, MAP2K4, MAPK13, MAPRE1, MORF4L2, NEDD1, NOS2, OPRM1, PKIB, PKLR, PNKD, PRKAR1B, RAB3D, RPS2, SLC39A11, SPINT2, TCF7L1, TUBA8, UGP2, VAMP2, YWHAH, ACTR3B, AGAP2, AKAP5, ARL15, ASS1, ATP9A, BASP1, C5orf30, CA10, CACNA1A, CDK16, CHD1L, CHRNB2, CKMT1A, CLP1, CPO, CYP21A2, CYP24A1, DAB2, DCAKD, ELOVL6, ENAH, EPHA5, FABP4, FBN2, FBXW7, FHL2, FLII, GRIK1, GRIN1, GRIP1, HOMER2, INPPL1, JPH1, LAMB3, LMO7, LMX1A, MAGI1, MAPK8IP3, MAPRE3, NCALD, NDST4, NELL2, NMNAT2, NR5A2, NSF, OFD1, PARD3B, PDPK1, PGP, PGRMC1, PJA1, PKIA, POLA1, PPFIA3, PRKCB, PTGER3, RAB11FIP5, RAB37, RAB3A, RAB3C, RABGAP1L, REEP6, RGS9, RIMS1, RNPS1, RTN4, SGTB, SH2D3C, SHROOM2, SLC2A13, SLC36A1, SLC39A10, SLC7A2, SNCA, SOX9, TAB3, TAF7L, TLN1, TOMM40L, TRPC6, UCHL1, VANGL2, WARS, WIPF1, ZC2HC1A, ZNF233, ABCB7, ABCG5, ACVR1C, ADAM23, ADCYAP1, ADCYAP1R1, AHNAK2, AMPH, ANKRD44, ANO6, ANTXR1, ANXA9, AP1S3, APBA1, APPL2, AQP9, ARHGDIG, ARX, ASB2, ATG4A, ATP2B3, ATP6AP1, ATPAF2, BAAT, BLCAP, BSCL2, BZRAP1, C10orf35, C11orf87, C1orf198, C1orf216, C3orf67, CAMKK2, CBFB, CCDC64B, CCNA1, CD99L2, CDR2, CDS1, CELF5, CHGB, CHM, CHRFAM7A, CKMT2, CNKSR2, CNN1, COMMD2, CPLX1, CRYGD, CSMD1, CTDSP1, CYP11A1, CYP26A1, DIMT1, DLG2, DLG3, DLX2, DMRT3, DNM1, DOC2A, DUSP19, DYSF, EFNA3, EFNA5, EHBP1, EID3, ENSA, EPB41L3, EPB41L4B, EPHA7, EPHB1, EPHB6, FAM129B, FAM50B, FAM65B, FATE1, FBXL16, FBXL2, FGL1, FKBP1B, FLCN, FLT3, FNDC4, FOXG1, FSCN2, G6PD, GABRB1, GABRB3, GABRG2, GAD1, GAD2, GAP43, GLCE, GLS, GNAL, GOLIM4, GORAB, GPR3, GPR63, GRAMD1B, GRAMD3, GRIN2A, GRIPAP1, GRM8, HPCAL1, HPGDS, HPRT1, HS3ST3A1, HS6ST2, HTR7, IGSF5, ITPR1, KANK2, KCNA3, KCNJ12, KCNJ3, KIF17, KIF3A, KIF5A, KIT, KLC2, KLF8, KLHL38, KRT17, KRT81, LAMC2, LDB2, LDOC1, LRRC1, LRRTM1, LY6D, MAP4K2, MAPK10, MAPKAPK2, MARCH11, MARCH4, MATK, MATN2, MESP2, MFNG, MICAL2, MPPED1, MTMR1, NCDN, NDST3, NKAP, NPHS1, NR1I3, NRXN1, OPRD1, PABPC1L2A, PACSIN1, PAK1, PALM, PAX2, PAX7, PDP1, PHKA1, PI4KA, PIP5K1C, PLIN5, PPARGC1B, PRKCG, PRKCZ, PRLR, PRNP, PRR5, PRSS12, PTPN4, QPCT, R3HDM1, RAB3GAP1, RAB6B, RALYL, RAPGEF4, RDH12, RDX, RGN, RGS20, RHOBTB2, RHOBTB3, RHOV, RNF183, ROBO3, RP2, RTN2, RTN4RL1, RXFP1, RYR2, SCAMP1, SCGB3A1, SCN2A, SCN2B, SCN5A, SCN9A, SERPIND1, SLC20A1, SLC2A3, SLC44A2, SLC9A6, SLIT2, SMC5, SNAP25, SNAP91, SNCB, SNX18, SORBS2, SOX2, SPDEF, SPICE1, SPRYD3, SSBP3, SSTR3, STAMBP, STAU2, STK17B, STMN3, STX1A, STXBP1, SULT4A1, SYN1, SYTL1, TBC1D9, TMEM123, TMEM2, TMSB10, TNIP3, TNNT2, TRAM2, TRIM55,

		TRPC5, TRPV6, TUFT1, TXNIP, ULBP1, USP12, VAT1, VGF, VSIG4, ZNF823
	purple	TUBB1, TNPO1, AGPAT2, MAPK3, S100A2
	yellow	TUBB4A, ERBB2, PGAM4, DNAJB13, EEF2, ALDOB, CCT7, HSPA2, ALDH1A1, COL4A5, DOK4, EEF1B2, MCOLN3, PARD6A, PLS3, PYGM, SGK2, SIX3, SUCLG1, UBQLN4, ABCA3, APOH, ARG1, B4GALT4, BAIAP3, BCAR1, BIRC3, CCDC33, CCDC80, CDC42EP4, CFH, CLDND1, CPSF4L, CRMP1, CYB5R2, DES, DOHH, EDIL3, ELF5, ERBB2IP, FA2H, FRYL, GJA1, HADHA, HEPACAM2, ID1, IL13RA2, INSM1, ITGB5, ITIH4, KCTD19, KIAA0930, KIF21B, LEF1, LLGL2, LOX, LRP2, MGST1, NEB, NMT1, NRIP2, PAX6, PHF23, PIK3R2, POLR3C, RAB41, RBP7, SEPT4, SLC12A2, SUN2, TFAP2A, TP63, TRAF1, VAX1, WDR33, WDR44, WDR92, YAP1
	green	TAF1, CCND1, BAG4, HSPA6, SMARCD2, ARHGAP4, ATF2, CACNA2D1, EYA1, GNG2, GRIA2, HEATR1, HECW2, KIF3C, LRPPRC, MED12, MED31, ORC4, PABPC5, PHLDA3, PPP3R1, PRKCE, PTCH1, RICTOR, TCIRG1, TET3, TTBK1, UBL4A
	darkgrey	FDPS, FGFR1, POU5F1, SMAD3
	orange	EIF4A2, POLR2A, MEIS1, SUMO3, BCAT1, ENO2, HOXB8, IGSF8, PICK1, SRC, ACTN1, ADAM19, AMOTL2, ARHGAP17, ASB5, CA13, CD9, CHRNA1, CHRNE, DLGAP2, EPHA2, EPHA4, EYA4, FZD5, GCH1, GNAO1, HOXD10, HOXD13, IGFBP2, IWS1, KRT75, LDHA, NDRG3, NFIL3, NPHP1, PBX3, PCBP3, PEX13, PITX1, PLCB1, PPP1R18, RAPSN, RND3, RRAGB, SCD5, SHROOM1, SIX2, SKP2, SLC47A1, SMAD5, TAX1BP3, UACA, UBE2Z, USP11, USP51, ZBTB32
	black	CDK2, MCM4, CCNF, GMNN, HIST2H2AC, TUBG1, HIST1H2BH, MCM3, MCM5, PSTPIP1, BRCA1, CCNA2, CCNB2, CDT1, CEP152, FEN1, HADHB, KIF14, MCM2, MYH6, NDE1, NUSAP1, ORC6, POLE2, RFC3, RRM2, SPAG5, SPSB4, SRSF7, TOP2A, TUBB
	magenta	MAT2A, AP1M2, COPB2, ATP6V1B1, MYO5C, CUL7, PAFAH1B2, RPLP0, NMNAT3, RPL4, TMOD3, BEX1, BTF3, CCDC39, COBLL1, CRYAB, DYNC2H1, EIF4EBP1, EIF5AL1, FBN1, GDI1, HDAC5, HIST3H2A, IDH3G, IGFBP5, IQGAP1, LRRC16A, MCC, MDFI, NACA2, NFE2L2, NME4, OBSL1, PPARGC1A, RPL10, RPL10A, RPL10L, RPS27A, SCNN1G, TBL3, TOM1L1, TRAPPC2, UBA52, UBXN4
LIHC	grey60	SUMO4, ACTB, PIK3R1, ACTA1, FOXP3, HIST2H2BE, PTGER3, SRPRB, CDX1, DNAJA4, HIST3H2A, XRCC3, AKR1C2, CD40, GZMB, HIST1H2BD, HIST1H2BH, HNRNPH1, LCP1, NOTCH1, PAK1, PC, PYGM, ALCAM, BCL2, BTNL8, CBLB, CHST12, CLIC5, DBI, DMC1, EHD1, FGD1, FUS, HNF1A, HS6ST1, IGF1R, IGSF21, ITGA4, LMNB1, MAP3K1, MAP7D3, PIK3CG, PLCB1, PNMA5, PRKCD, RACGAP1, RAD51, TAP1, TTN, VIM, WDR76, ADAM19, ADAMTSL5, AKR1B1, AMPH, ANKRD13D, ANKRD44, ARHGAP4, ARHGEF6, ARPC1B, ART3, BATF, BATF3, BLVRB, BMF, C9orf116, CACNB3, CASP2, CCDC36, CCDC85B, CCNB2, CCND3, CD6, CDC25C, CDC7, CDCA4, CDS1, CEP55, CHST3, CHTF18, CIT, CNPY4, CYP11A1, CYP2S1, DAB1, DOK5, ELOVL5, ESR2, FAM46C, FANCD2, FBN2, FGFR2, FHL3, FLI1, FLT3, FOXS1, FSD1, GAL3ST1, GFPT2, GLIPR2, GPR18, GPSM1, GRIP1, GRM2, HCLS1, HDAC7, HMGCS1, HMOX1, HSPG2, IDI1, IFI16, IFIT2, IFIT3, IKBKE, IL7R, IRF5, ISLR, KIAA1524, KIF15, LEPR, LGALS1, LIG1, LILRB2, LMO2, MDFI, MEF2C, MGAT5, MYBPC2, MYBPC3, MYEF2, MYH15, MYO1F, MYOF, NCKAP1L, NFATC1, NFATC2, NFYB, NIN, NLRP1, NOTCH2, NREP, NYAP1, PCDHA4, PHF19, PHLDA3, PHOSPHO1, PIF1, PLCB4, PLK4,

		POF1B, PPP1R16B, PPP2R2B, PSMB9, PTCH2, RASGRP3, RETSAT, RGN, RHBDD1, RIBC2, SEPT1, SEPT8, SIRPB1, SLC36A1, SP140L, SPINT2, STMN1, STX3, SYT1, TBC1D8B, TFAP2A, THAP10, TMBIM6, TMEM216, TMOD2, TNFRSF21, TNFRSF25, TPM2, TRAF5, TRPM4, TSHR, VSIG4, WDHD1, ZNF257, ZNF492, ZNF676, ZNF98
	black	CYFIP2, FOXD4, PLG, SLC2A13, AMOT, ARRB1, CDKN2D, COBLL1, DLX3, HSD17B14, HSPB9, KIF1A, KIF3A, MAPK11, MAPK12, MYO5A, PACSIN3, RGS20, TLE1, TUBA8
	yellow	HSPA1B, AR, BCAT2, FYN, KHDRBS1, ZWINT, GFPT1, LCOR, LPL, LRP1, THNSL2, ACY3, APOE, CALR, CASK, CCNB1, CEP128, CSMD2, FIGNL1, GRB7, KDM3A, KLF6, LYZ, MAP3K8, MCM6, NUP62CL, PGM5, PLBD2, PLK2, PRPS2, RPL10L, SDSL, SMAD6, TNFSF11, TNS3, TREH, ZNF460, ZNF607, ZSWIM5
	greenyellow	JUN, HNF4A, MAT2A, HNRNPC, ZNF563, MYH10, ACTG1, ERBB3, HIST1H2BO, HSPA2, PHGDH, ATP6V1B1, ELK1, MYH11, TUBA1A, ZNF331, ACTA2, APOD, ARFGEF2, CBLC, HS3ST2, MYO1B, NCK2, OGDHL, PFKP, PGLYRP2, SHBG, TMEM25, ZFP37, AJUBA, APOA1, CACNA1A, CACNB2, CCDC146, CCDC8, CHST10, CHST9, CLIP4, CMYA5, CNGB1, CTBP2, CYP26A1, DAB2, DBN1, DTNBP1, FHL2, GSTP1, HIST1H3A, MAPK10, NMI, NPHP1, NR5A1, PNMA1, PRNP, SLC22A5, SOX10, SOX8, SPTLC3, SPTSSA, TFAP2C, ZNF470, ZNF549, ZNF610, ZNF649, ZNF667, ZNF793, ABCA4, ACSS1, AKT3, ANXA4, APCS, ARNT2, ASNS, ATF3, B3GALT1, BARX2, BHLHE40, BLMH, C14orf105, CACNA2D1, CAV1, CBR1, CCL2, CD1D, CD9, CETP, CNGA1, COX4I2, CRHBP, CRISP3, CYP17A1, CYP4F11, DAPK1, DCN, DES, DLX1, DTX1, EFEMP2, EGLN3, EPHB4, F2RL1, FAM134B, FAM189A2, FBLN1, FBLN5, FCGR3B, FGF9, FHL1, FREM2, FZD8, GALNT12, GATA2, GRK5, GSTT2, HCN2, HCN4, HIST1H1E, HK1, HOXD10, HSD17B2, HSD17B3, ID2, ID3, IGFBP6, IGFBP7, IL1RAP, IL22RA1, INMT, ITGA7, KCNN2, KDELR3, KLF5, KRT6B, LDHB, LIMCH1, LPIN1, MAP1LC3C, MATN2, MB21D2, MITF, MMP7, MSX2, MT1H, MYH14, NEXN, NOTCH3, NPHP4, NRG1, NRG2, PADI4, PALM, PBX1, PDZD3, PHLDB3, PIK3AP1, PIP4K2A, PLAT, PLK3, PPP1R12B, PRKX, PRTFDC1, PSPH, PTGIR, PTK6, PYCR1, RAB38, RBM4B, RET, RGS16, RIPK3, ROR2, SEMA3F, SH3YL1, SLC25A41, SLC2A3, SLC35A2, SLCO4A1, SNAI1, SNX12, SPSB4, SULT1C4, SYCE1, TALDO1, TBXA2R, TCF4, TEKT3, TFEB, TFF1, TINAGL1, TLR4, TNNT3, TNS1, TRIM15, TSLP, TTYH3, USP51, WEE1, WNT5B, YPEL4, ZC3HAV1L, ZDHHC13, ZFP28, ZFP36, ZNF160, ZNF354A, ZNF426, ZNF439, ZNF454, ZNF471, ZNF662, ZNF677, ZNF93
LUAD	blue	H3F3C, TAF1, COBL, MDFI, ZNF267, ADAMTS6, ANKS1B, CA9, DDX39A, FHL2, FHL3, HIST2H2AC, ITGB7, MAPT, PCSK2, PDE4DIP, PDLIM7, PIM1, PPP1R18, PRKCE, SCG5, SLC1A2, SLC34A2, SOCS1, SPRY1, TAP1, TOB1, ZNF132, ZNF658
	green	UBC, HSPA1B, VCAM1, ALK, ALPK2, ANKRD1, AQP1, CEBPA, CLSPN, COL12A1, DIO2, EPHB2, FOS, FOXP3, GPR176, HBG2, HIST1H1E, ISL2, KIF26B, LGALSL, NRGN, OTUD1, PAPSS2, SDR16C5, SLC5A7, SPON2, STRA6, SUMO4, SYNGR3, TERT, TMEM132A, VCAN, WT1
	turquoise	EYA1, FOXI2, ACSL6, ASB5, CASC5, CCDC151, CCT6B, CHGB, CHST8, CUBN, CYP1A1, CYP26A1, CYP2B6, CYP2C8, DFNB31, FGFR3, HIST3H2BB, KRT1, LRRC4C, MYEF2, NECAB2, NEUROD1, NONO, NPHP1, NTN1, PYGL, SIX2, SLC3A2, SOX2, SULT2B1, TALDO1, TF, TNFRSF19, TRAF5, ZNF154, ZNF214
	black	ACTA2, ATP6V1B1, SLC2A4, ATP6V0D2, EIF5AL1, FBN1, MYOC, PCOLCE, PLEC, PYGM, SERPINB1, ACTN2, ADRA1D, ALDH1A2,

		APLN, BGN, C2orf48, CAPN3, CAV3, CCDC8, CRYAB, CTSG, CYP24A1, CYP2A7, DES, DNM1, DPEP2, ELANE, F10, FAT3, ITGAV, LTBP4, PDGFRA, PGK2, PLSCR4, SH3GL3, TAGLN, TMOD1, TNXB
	red	HSPA6, TUBB2B, ALDH1A1, CADPS, CYP4F12, HMGCS2, KIAA1549, PIWIL1, PIWIL4, PSAT1, TBX2, TEAD2
LUSC	pink	CDX1, HMGA2, LDHAL6A, TPM1, ALDH1A3, CENPV, COL5A3, HMGCS2, SOX9, TAF1, TNF, TNNI3, ZNF433, ZNF439, ZNF492, ZNF662, ZNF98
	brown	TUBB4A, AR, CAV1, EFNA2, NGFR, BEX2, CYP2S1, CYP2W1, EPHA6, EPHA7, EYA1, FOXC2, HAND2, HIST1H2BH, HOXA7, HOXD13, KRT31, NACA2, NR5A1, NTS, PRKCA, SCD5, ZIC1, ZIC2
	purple	CYP26C1, ARHGEF40, CYP4F2, CYP7B1, DLG4, FAXC, FOLH1, STC2, TKT, ZNF267, ZNF347
	royalblue	NOTCH2NL, ZNF619, APLP1, CHST3, CRCT1, GFOD1, SLC22A23, SULT2B1, ZNF667, ZNF676
	grey60	C5orf60, CHST11, CHST4, CST4, KRT40, TSGA10IP
	tan	MAT1A, ISYNA1, PKLR, TUBAL3, CYP39A1, CYP4F22, TUBA8
	red	UBC, HNF4A, HSPA1B, TUBA1A, HSPA1A, CALML3, LYN, FLNA, HIST3H2BB, NTRK1, ALDH1A1, SLC2A4, ATP6V1B1, FOXP3, LRRK2, ACTA2, FN1, MYH11, NCK2, PRKAA2, ALDOB, LIMA1, NAP1L2, KCNMA1, PGAM4, DNAJB13, GSPT2, H3F3C, MAP1B, MYH7, MYL6, TTN, ATP6V0A4, GRIN1, NPHP1, PXN, UBD, ACTC1, ALDH2, CFTR, DLG3, DYNLL1, FKBP1B, HDAC4, HIST1H2BM, SH3GL3, SORBS2, CDC14A, PABPC5, ACTN2, ARRB1, COL5A1, H2BFWT, MAPK8IP2, PAPOLB, RPL9, RPS27, SDS, SOCS2, SOCS3, SULT1E1, VIM, ZNF468, CAMK1G, CCNB1, ELF5, ESRRB, FGFR4, GRIN2A, KIT, LIN7A, MCC, MCOLN3, MYO1F, NONO, NOVA1, NR4A1, OGDHL, OLIG1, PCBP3, PRLR, RUNX2, SCIN, SH3YL1, SSR4, SULT1A1, TRIM63, ALK, ATF3, CAPN2, CDH2, COL9A1, DYSF, EPAS1, GPC3, GPD1, HCFC1, HSPB1, IL1B, IL4R, KIF11, LDHAL6B, LDHC, LMNB1, MAP2, MMP9, NCF1, NFATC2, NRP1, PGK2, PLXNA3, PPARGC1A, PYGM, RHOB, SH3GL2, SIRT4, SLC2A3, SLC47A1, SMAD6, SMARCC1, SMARCD3, SULT4A1, ACADSB, ADCYAP1, ALDH1A2, ARHGDIG, ATP10B, ATP2B2, AXL, BATF3, BEGAIN, BIN1, BTNL8, C3, C9, COL11A1, CPM, CREB5, CRP, CYBRD1, CYP46A1, DAB2, DLG2, DNAJC12, EGF, EGR1, ELMO1, ERBB4, ERG, FHL1, FKBP5, FOXD2, FUS, GFAP, GLI3, GLP1R, GNAO1, GNAT2, GPR3, GRIA2, HBA1, HBB, HCK, HCLS1, HES1, HLF, HMGCR, HOXD8, IL6, ITGA4, KIF17, KIF4A, KRT10, LIPH, LMO2, LTF, MAG, MAP3K3, MAST1, MID1IP1, MUSK, MYBPC1, MYOC, NCAPG, NFE2, NOS1, NT5DC2, NUP210, PAX8, PRDM16, PRKAR2B, PRMT8, RAD54L2, RBM24, REEP6, RGS2, ROBO1, S100A4, SCN2A, SHANK1, SOCS1, SOX5, SPERT, SPTA1, SULT1A2, SULT1C4, TAF1L, TEX11, TFAP2A, TGFB1I1, TKTL2, TMOD1, TNS1, TRIM9, USHBP1, ZBTB16, ZNF429, ZNF454, ZSCAN1, ABCC2, ADAM15, ADAM22, ADRA2A, ANK1, ANP32D, APOB, ARHGDIB, ARMCX3, ATP2A3, BAP1, BATF, BIRC3, BMP4, C6, CACNA2D1, CACNB4, CAPN3, CARD10, CCDC148, CCDC33, CDON, CENPH, CHGA, CHRM4, CHTF18, COL1A2, COL6A1, COL6A2, CPLX2, CYP26B1, DDC, DHRS2, DLGAP2, DTX4, EGR2, ENAM, EPHA4, EPOR, EVL, FBLN2, FCGR2A, FGA, FGF13, FGFR2, FGFR3, FLNC, GABRA2, GABRG2, GAL3ST1, GFI1B, GK, GNAL, GPR183, GRIA1, GSTM5, HECW2, HELLS, HIST1H1E, HOPX, HOXB5, HS3ST2, HSD11B1, HSH2D, IGFBP7, IL16, IL1R1, IL2RG, ITGA2, ITGB7, JAK3, KCND2, KCNJ11, KCNJ8, KIF4B, L1TD1, LIX1L, LRIF1, MAP1LC3C, MATK, MCEE, MED12, MEOX2,

	MMP2, MPZ, MST1R, MTTP, MUC1, MUC2, MYEF2, MYH15, MYH2, MYL9, MYOCD, NCAM1, NCKIPSD, NEDD9, NFASC, NGB, NOX4, NPAS2, NPPA, NR5A2, ORC1, PBXIP1, PCK1, PCSK1N, PELI2, PHLDA3, PKIA, PLA2G4A, PLAC8, PLEKHH2, PNMA2, POU2AF1, PPP1R9A, PRSS21, PTGS2, PTK6, PTPRD, PYHIN1, RAB6C, RALYL, RGS3, RGS4, RGS7BP, RGS9, RHOU, RORC, SCML2, SDCBP2, SDSL, SERPINA1, SERPINA3, SGOL1, SGPP1, SHANK3, SKA3, SLAIN1, SLC17A8, SLC18A2, SLC27A2, SLC27A3, SLC4A7, SLC6A3, SLIT2, SLN, SORBS1, SOX10, SOX6, SPARCL1, STAT5A, SULF2, TAGLN, TCIRG1, TFF1, TGFBR3, TGM2, THEMIS, TLE6, TMOD2, TRAIP, TRIM69, TRPM3, ZNF423, AATK, ABCA3, ABCB7, ABCC9, ACP5, ADAMTS12, ADAMTS2, ADCY5, AEBP1, AGER, AGR2, AHCYL2, AIF1, AKAP2, AKAP5, ALAS2, ALDH1L2, ALDH3B1, ALOX5, ALPP, ALPPL2, ANKRD55, ANTXR1, APOH, AQP3, ARHGAP9, ARHGEF15, ARMC12, ASRGL1, ASS1, ATP11C, ATP13A4, ATP1B2, ATP6V0D2, ATP8B2, ATP8B4, B3GNT3, BAAT, BACH2, BAMBI, BCL2A1, BCL2L10, BCO2, BEX5, BGN, BMP6, BMX, BSPRY, BTG2, C4BPA, C4BPB, C5AR1, C8B, CA13, CA14, CA7, CA9, CACNB2, CADM2, CALCOCO1, CALD1, CAPN9, CASQ2, CBFA2T3, CBR1, CBX7, CCDC158, CCDC36, CCDC51, CCDC80, CCDC88A, CD109, CD19, CD207, CD209, CD36, CD4, CD55, CD63, CD69, CD86, CD97, CDC25A, CDC25C, CDCP1, CDH15, CDK20, CDKL4, CEACAM21, CEBPE, CFHR3, CFI, CGN, CHAD, CHIA, CHRD, CHRM1, CHST13, CIITA, CKMT2, CLEC11A, CLEC12A, CLIC2, CLIP1, CLMP, CMTM5, CNDP1, CNN1, CNTFR, COL14A1, COL3A1, COL7A1, COMP, COX4I2, COX7A1, CP, CPLX1, CPNE6, CREB3L1, CREB3L2, CRIP1, CST1, CTNNA3, CTXN1, CUBN, CXCL11, CXCL14, CXCL2, CXCL9, CYP17A1, CYP1B1, CYP21A2, CYP2A7, CYP2F1, CYTH4, DCN, DDIT4, DEPDC1B, DGAT2, DHRS9, DIRAS3, DNM3, DOK5, DPF3, DPP4, DTX1, DYNLRB2, EBF3, EEA1, EFNB1, ELAVL4, ELF3, ELMOD1, ELN, EPDR1, EPHA5, ERN1, ETS1, FAM174B, FAM184A, FAM49A, FAM92A1, FAP, FASLG, FBP1, FCER1G, FCGR3B, FCHO1, FCRL4, FES, FGD3, FILIP1, FLT3, FMNL1, FMO3, FMO4, FMO5, FOLR1, FOXA3, FOXJ1, FOXQ1, FSCN2, FST, FXYD7, FYB, GAB2, GABRB3, GADD45B, GATA2, GCGR, GCM1, GCNT2, GDF15, GFRA1, GLRB, GLT8D2, GNG13, GP1BA, GPC1, GPC5, GPR143, GPR89B, GPRASP1, GRM7, GZMB, HAAO, HAL, HAPLN2, HDC, HEG1, HES4, HES5, HKDC1, HMGCLL1, HMOX1, HPD, HSPB6, HSPB8, HTR2A, HVCN1, HYAL1, ICA1, ID3, IFITM2, IGF1, IGFBP1, IGSF21, IKZF1, IL12RB1, IL1RL1, IL1RN, IL32, IL33, ITGB4, ITIH2, ITLN1, ITPR2, IYD, KANK2, KCNAB2, KCNJ2, KDR, KEL, KIF15, KIF20A, KIF26B, KIF5C, KIR2DL4, KL, KLF5, KLHL6, KLRF1, KPNA7, KREMEN2, KRT2, LAD1, LAMA2, LAT2, LCK, LGALS12, LGALS2, LIF, LMCD1, LMO3, LOR, LRIG1, LRP2, LRRN1, LRRTM2, LTA, LYPD3, LYZ, MADCAM1, MAGEA10, MAGEA8, MAP6, MAPK15, MASP1, MEOX1, MEP1A, MGAM, MKI67, MOBP, MORN3, MPP1, MUC15, MXRA5, MYBPH, MYO7B, MZB1, NAGK, NCCRP1, NCF2, NCF4, NDNF, NDRG2, NEXN, NID1, NLGN3, NLRP4, NOS3, NPHS1, NR0B2, NR1H3, NRGN, NTRK3, NUDT11, NXF3, OAS1, OR51E2, OSM, PACSIN1, PADI3, PAK3, PALM, PARP14, PCDHB3, PCDHGB1, PCOLCE, PDE2A, PEX5L, PFKFB4, PGM5, PID1, PIK3AP1, PIK3CG, PIP, PIP5K1B, PITX1, PKIG, PLA2G16, PLCB2, PLD5, PLEKHO2, PLP1, PLVAP, PLXNA2, PLXNB3, PMEPA1, POLR3GL, POPDC2, POU2F3, PPFIA4, PPP1R12B, PPP1R14A, PPP1R1B, PRDM1, PRKCB, PRKCQ, PRKD1, PRODH, PROS1, PROX1, PRUNE2, PSG5, PSTPIP1, PSTPIP2, PTGER2, PTGER3, PTGS1, PTPRB, PTPRO, PURG, PXDN, PZP, QPCT, RAB27B, RAB8B, RAP1GAP, RARG,

		RARRES3, RBMXL2, RBP7, RFPL2, RGL1, RGN, RGS11, RHBDF2, RHOF, RIBC1, RIN3, RNF175, ROBO4, RP2, RPE65, RPS6KA2, RRP9, RYR2, S100A11, S100A2, S100A3, S100A6, SATB1, SCD, SCG2, SCG3, SCGB1A1, SCGB3A1, SCN4B, SCN8A, SCNN1A, SCTR, SCUBE3, SDC2, SDPR, SDR16C5, SEC14L4, SECTM1, SELE, SELENBP1, SEMA3G, SEMA6B, SERPINA6, SERPINB2, SERPINB5, SGCG, SH2D1A, SH2D1B, SH2D3C, SHC4, SIGLEC9, SIRPB1, SLC13A3, SLC16A6, SLC22A16, SLC34A1, SLC40A1, SLC41A2, SLC43A3, SLC4A1, SLC6A14, SLPI, SMAD9, SNAP25, SNCAIP, SNX32, SOAT2, SOX8, SPATA12, SPDEF, SPI1, SPIN2B, SPIN4, SPINK7, SPOCK1, SPOCK2, SPOCK3, SRMS, SST, SSTR3, STAP1, STARD4, STEAP4, STK17B, STMN2, STOML3, STX1B, SULT1C2, SUSD3, SYTL2, TCEA3, TET1, TH, THBS2, THBS3, THBS4, THNSL2, TIE1, TLE2, TMEM190, TMEM55A, TMEM79, TMSB10, TNFAIP8L2, TNN, TOX2, TOX3, TP53INP2, TRAF5, TRIM29, TRIM55, TSC22D3, TSHR, TSPAN13, TTLL10, TTYH2, UACA, UBASH3A, UGT8, UPP2, UTRN, VAV1, VPS37B, WAS, WEE2, WIPF1, WNK4, XAF1, XDH, XYLB, ZAP70, ZEB2, ZNF229, ZNF326, ZNF442, ZNF816
SKCM	blue	SUMO4, ACTG2, HIST1H2BD, HIST1H2BO, AKR1B1, ALDH1A2, CORO2B, ACTA2, ACTC1, BCOR, EIF4A2, GJA1, GLUL, GSPT2, KRR1, MDFI, NACA2, NTN1, PTGER3, STAT1, USP9X, AKR1C3, ALDH2, APBB1, AR, BBOX1, CD36, CD9, CDH23, CFTR, CHM, CTPS2, CXCL16, CYP19A1, CYP2S1, DNM3, ECM1, EFEMP1, EGLN3, EIF1AX, ERG, FOLR1, FTL, GLI1, GLI2, HTR2A, MARCH2, MAT2A, MYLPF, MYO3A, MYOC, NFE2, NTRK1, NUAK1, NXF3, OSBPL8, PCDH15, PHLDB3, POC1B, POU3F1, RAB31, REST, RPS6KA3, SFRP4, SGCG, SSX5, STMN2, SULF1, THOC2, THRB, TSHZ2, WNT3A, ZNF426, ZNF662, ZNF714
	brown	MCM2, LIMA1, ACTB, TAF1, ACTA1, CCT6B, CD109, CDCP1, CDK18, CLK1, CSTF2, DSG1, DUSP26, DUSP5, FST, FTSJ1, HNRNPM, HSF4, KCNIP1, KCNIP2, KIAA1107, L3MBTL1, LENG8, MEF2A, MSTN, MVP, S100A10, S100A11, SRPK3, TSGA10, ZCWPW1, ZNF823, ZNF860
	turquoise	EEF1A2, IKBKG, SOS1, CFL2, CLTCL1, SH2D4A, BCR, PDHA1, REL, ATP2A3, BCL2L1, CDKN1A, FOXP3, ITSN2, NOTCH2NL, PRDX2, TUBB2A, ARG2, BCAP31, CDX1, CYB5R2, FASN, FKBP5, GDI1, GRIA1, HCLS1, HNRNPH1, IKBKE, LRRK1, LYN, POU2F1, PSMB9, SSBP3, STAM2, TNFSF12, TNIK, TRAF1, UBD, VCAM1, XRN1, ADAM22, ADD3, APOL2, ATP1A3, BIRC3, CEBPE, CHGB, CHST4, EPCAM, FGR, HCK, HS6ST2, KCNJ10, LCP1, LDOC1, MAGI1, MUC13, NR5A2, NTN4, OBSL1, PIK3C2B, PLXNA2, PRAM1, PRKCSH, PSMB8, PTK2B, PTPN6, RAC2, SKAP2, SLAIN1, STXBP2, TNF, ABCB11, ABCC3, ACTBL2, ACY3, ADRBK2, AGAP2, AHNAK2, AICDA, AKAP5, ALOX5, ANGPTL6, APBA2, AURKC, BCL2L14, BDKRB1, BLK, BSG, C1QB, CCL17, CCND2, CD163, CD22, CD4, CD5L, CORO1A, CSDC2, CXCR4, DGKE, DHCR7, DNAJC5B, DOK2, DPEP2, DPPA4, DUSP1, FAM78A, FERMT3, FLT3, GAL3ST2, GALNT14, GCH1, GIMAP5, GK, HBE1, ICAM2, IGLL1, IKZF1, IL16, INPP5D, ITGB2, ITGB7, ITK, JAK3, JSRP1, KCNA2, KCNE4, KIAA1549, KIF1A, KRT15, KRT36, KRT75, LARP6, LAT, LAT2, LMO2, LRFN3, LYL1, MAST3, MED14, MEP1A, MME, MORF4L2, MPP3, MYO1F, NCOA3, NLRP1, PARD6A, PARVG, PHF8, PIM1, PIM2, PLS1, POU2AF1, PRKCB, PRKCQ, PSMA8, PSTPIP1, PSTPIP2, PTPN18, PXMP2, RAB34, RAB8B, RAPGEF4, RASGRF1, RTN2, SCN2A, SEC14L4, SKAP1, SLC18A1, SLC25A14, SLC39A6, SNX7, SSTR3, SULT4A1, TACC2, TANK, TAZ, TBC1D10C, TCL1B, TECR, TIMM8A, TNFRSF1B, TNFSF13B, TRIM6, TRIP6, UBASH3A,

		UBL4A, UNC5A, VPS13A, VSIG4, WAS, ZC3HAV1, ZFP36, ZNF182, ZNF484, ZNF675
	greenyellow	CCDC8, ATP1B1, BAZ1A, FOXG1, HOXC13, MYO1D, MYO5B, PRPS2, RIF1, RPL39L, SOCS4, SPRY1, XPO1
	purple	ESR2, FLNA, ACTN3, CTNND2, EMD, EVC2, F3, FBLIM1, FKBP1B, H3F3C, KRT86, MAPK11, PDE4B, PORCN, WNT5A, ZBTB33
	royalblue	DKC1, ENO2, F8, GTF2H2, LOX, PGK1, TLL2, VWF
	darkturquoise	HNF4A, PABPC5, ASF1B, IFT27, ITIH2, KIF20A, TOP2A, YPEL3
	darkorange	CNKSR2, DLGAP2, PRKX, PYGL
	pink	CAV2, FBXL16, KRT2, MLC1
	green	NEDD4L, TUBA1A, LRRK2, PTCH1, DNAJA4, JUN, KAT2B, TF, FN1, GFPT2, TMOD1, CHST11, EGFR, IRAK1, ISG15, KIF5A, KYNU, LIN7A, MYH13, MYH7, OGDHL, RAB3B, TFAP2C, ABCB7, ACOT9, ADAM12, ALK, ARSJ, B3GNT2, CHST7, CNNM1, CNTNAP4, CTHRC1, CYP24A1, CYP2J2, DAPK1, DEPDC7, DLG3, DNAJB9, DPYSL3, EDIL3, ERRFI1, FGF2, FMNL2, FSTL3, GAL3ST1, GALNTL6, GCK, GNAO1, GRIA2, GRIN2D, HNRNPH2, HOXD12, HOXD8, HPCAL1, HTR7, ICA1, IGF2, IRAK3, IRF4, ITGA7, KLF10, KRT18, LAMC2, MAPT, MATN3, MESP2, MYH8, MYO5A, NID2, PELI1, PIEZO1, PLAUR, PPP2R2C, RANBP10, SDC4, SEMA6A, SERPINB2, SLC2A13, STAC, STK32A, SV2A, SYNM, SYTL4, TCF4, TLX3, TMEM132A, TPM1, TSPAN5, USP51, WNK4
	orange	CDK2, HSPA2, ATP10B, FOS, PPP2R2B, RUNX2, ATP6V0A4, CACNA1A, FHL1, HEY2, MAT1A, SEC24A, ANKLE1, ART3, AZGP1, CASKIN1, CDH1, CLMN, CPB2, CRYAB, CYP1A1, CYP39A1, DTNB, EYA1, GAPDHS, GATA4, GDA, GEM, GPD1L, GREB1, HEY1, HHATL, IDS, ITGA4, MEIS2, NELL1, NR2E1, NUP210, PAM, PAX6, PDK3, PRKAG3, RAB6B, RASSF2, RYR1, SEPT4, SERPINB9, SHROOM3, SLC7A5, SNTB1, STXBP5L, SUV39H1, TRIB2, USP15
	black	RORB, RORC, TUBA8, ALX4, BATF3, DPYSL4, HLF, LIMS1, LIPH, PEG3, SCD5, SLC47A2, SOX2
	magenta	HSPA6, ALDH1A1, ATP2B2, ATP6AP2, ABCG1, APOE, BEX1, CCDC68, CLCN7, DSP, ETFB, FBP1, FCGR2A, LGALS3, LGR4, LOXL4, PLS3, PRKAA2, TEX11
STAD	green	AGPAT2, EFNB1, EPHB2, FGFR3, GFPT1, KRT8, LNX1, STX19, SULT1A2, TYRO3
	black	ACTB, HIST3H2A, HSPA1L, JUN, HSPA1B, FOXP3, HSPA5, NEDD4, TUBB2B, ZNF57, HDAC6, ARRB1, UBQLN2, NTRK1, ATP6V1C2, SOX2, SPTAN1, PGAM2, HSPB1, TUBA1A, TUBB4A, ACTN1, SDS, ACTA1, ESR1, OGDHL, HDAC4, HIST1H2AB, HIST1H2AH, MAP1LC3A, GCK, SATB2, ALDH1A2, GABARAPL1, GJA1, HIST1H2BM, LRRK2, PABPC5, ACTA2, ATP6V0D2, CAMK1, CTH, CYB5R2, HSPA2, MAGEA1, SLC25A5, SNCA, STAT1, TGFBR2, TPM1, ACTC1, ACTN2, CAMK1D, CASK, DBN1, DPYSL2, FBXO32, FN1, GRID2, PPFIA3, PRKAR2B, SFN, VIM, AHNAK, CAV1, EIF4EBP3, GFPT2, GJB2, GNAI2, GPC5, GSPT2, HNRNPH1, INADL, KDELR3, MAGEA6, MYH10, MYO5A, NME4, NTN3, PKN3, PYGM, RIPK3, SNTA1, TAGLN2, TANC1, TFAP2A, TUBB6, ACTG2, ACVR1C, AKT3, ALDH1A3, AURKB, CACNA1A, CCNB1, CCNB3, CENPE, COL11A1, ERG, EYA1, HELLS, HOXB13, HOXB9, KHDRBS3, KRT17, MAGED2, MAPK11, NPHP1, OBSL1, PARK2, PBX1, PFN2, POLR2J2, PPP2R2C, PRPF40B, SH3GL3, SLC6A12, TCEA2, TF, TRIM29, TRIM63, USP2, ZNF93, AADAT, APLF, ARHGAP8, ASAP2, BMPR1B, C1QTNF9, CCDC8, CDC20, CDC7, CFL2, CHRNA1, CST4, CYP39A1, DDI1, DHRS2, DLG4, DNAJB5, DST, DYSF, EPB41L2, ERLEC1, EXOC6B,

	FAM168A, FOXA1, FOXD3, FOXD4L1, FOXF1, GRIN2A, GSN, GTSE1, HES1, HIST1H2BL, HOOK2, HTR2C, IFI30, IKBKE, INA, ISYNA1, KCNH2, KCNMA1, KIF11, KRT16, LIN7A, LRP1, LYPD3, MACF1, MAP7D1, MICALL1, MSN, MYOCD, NOTCH3, OPRD1, OPRM1, PAK7, PBX3, PMM1, PNMA1, PPP2R2B, RAD54L, RBPMS, RGS2, SAFB, SEPT5, SLC2A4, SOX17, STXBP5L, SULT2B1, TCL1A, TEAD2, THNSL2, THRB, USP11, WASF1, ZNF25, ZNF560, ACOX2, ANXA6, APBB1, ARNT2, ATP2B3, ATXN1, BACH2, C1orf94, CA10, CAGE1, CCDC88A, CD109, CHST13, CLU, CNN3, COL6A2, CREB3L2, CSPG4, CYP2A7, CYTH3, DAZAP1, DEF6, DIAPH2, ELANE, ENAM, EPAS1, EPB41L3, ERBB4, FAIM2, FAM83D, FBXL16, FEZ1, FKBP5, FLNA, FOXI2, GATA1, GATA3, GFAP, GIPC1, GRIA2, GRK5, HDAC7, HIPK2, HMGCR, HMMR, HOXA10, HOXD10, HPGDS, HS6ST2, HTR7, ICAM5, IFT81, IGFBP5, IL1B, IL1R1, KCNK1, KIF1A, KIF20A, KIF3A, KLF10, KRT1, KRT27, LDHD, LGALS1, MAGEA10, MAP2, MAPT, MBNL1, MEIS1, MMP1, MUC1, MYO7A, MYRIP, NAAA, NAP1L2, NCALD, NCAPG, NDN, NEK7, NMNAT2, NOVA1, NOX5, NTAN1, NXF3, OAS3, PAK3, PGM5, PIWIL1, PPP2R5B, PRAME, PRKAA2, PRSS23, PTGR2, PTPRS, RAB27B, RAB3IL1, RAB8B, RHOB, RHOBTB3, RRAS, RRM2, RYR1, S100A10, SATB1, SCN3A, SDCBP2, SERPINB5, SERPINB6, SFPQ, SH2D2A, SLAMF7, SLC25A12, SMAD9, SMARCA1, SMARCD3, SNCAIP, SORBS3, SPSB1, ST5, TAF9B, TBC1D9, TBL1X, TLE2, TLE3, TMEFF1, TMOD1, TNC, TRAIP, USP51, UTRN, VSNL1, VWF, WLS, XBP1, XIRP2, ZBTB16, ZNF311, ZNF492, ZNF530, ABCB1, ABCB4, ABCC4, ACSL6, ADAMTSL4, ADAMTSL5, ADRA1D, ADRB1, ADRB2, AGR2, AGTR1, AHCYL2, AKAP2, ALK, AMIGO1, AMIGO2, AMPH, ANK2, ANKRD7, ANKS1B, ANTXR1, ANTXR2, ANXA4, AP1S3, APBA1, APOBEC3B, AR, ARHGEF6, ARL2, ASF1B, ASTL, ATG4A, ATP2B4, ATP6V1G2, AXL, BAMBI, BBS10, BEX2, BIK, BLK, BLVRA, BTG2, BUB1B, BVES, C16orf59, C1orf216, C6, CALD1, CAMK2A, CAMKV, CAMSAP2, CAMSAP3, CAP2, CASP10, CCDC102A, CCDC146, CCDC64, CCNA1, CCNI2, CCNO, CD164L2, CDC25C, CELSR3, CHAC1, CHFR, CHN1, CHSY3, CKB, CKMT2, CLDN3, CLDN5, CLGN, CLIC4, CLSPN, CNIH2, CNKSR1, CNTN2, CNTNAP3, COL18A1, COL4A1, COL4A5, COL6A1, COL9A2, COPZ2, CORO2B, CORO6, COX4I2, CPNE6, CPNE8, CPVL, CRB1, CRHBP, CRIM1, CRIP2, CSGALNACT1, CTF1, CTSG, CYBRD1, CYGB, CYP1B1, CYP21A2, CYP26C1, CYR61, DAB2, DEGS1, DGKG, DHH, DLGAP1, DLX5, DMD, DNASE1L2, DPYSL5, DTNA, DTX1, DUSP9, DYNC1I1, DYNC2H1, DYNLRB2, EAF2, EDNRA, EFEMP1, EFEMP2, EFHD1, EFNA3, EFNB3, EHD2, EID3, EIF2AK3, ELOVL4, ELOVL5, ENPP2, ENPP6, EPM2AIP1, ERRFI1, ETV1, EVL, EYA2, F13A1, FABP4, FABP5, FAM129A, FAM212A, FAM212B, FAM46B, FAM83A, FBLN1, FBLN2, FCER2, FCHO1, FERMT2, FES, FGF1, FGFBP1, FGFR1, FGFRL1, FHL1, FHL3, FLG2, FLNC, FLT3LG, FLT4, FMN2, FMO3, FMOD, FOXP2, FOXS1, FRMPD1, FZD8, GABRA3, GADD45G, GAP43, GDF15, GFOD1, GJA4, GJB3, GLB1L2, GLI1, GLI2, GLI3, GLIS3, GNAI1, GNG4, GPC3, GPR183, GREB1, GRIA1, GRIN2D, GSTM5, HAL, HES6, HGF, HOMER3, HOOK1, HOXA9, HOXB7, HOXC6, HOXD1, HOXD8, HP, HPCAL4, HSPG2, HTR2B, IGFBP3, IGSF3, IL1R2, IL33, INHBB, IRF2BPL, IRS4, ITGA5, ITGA7, JDP2, JMY, KCND3, KCNE1, KCNJ11, KCTD15, KCTD17, KDR, KIAA0922, KIF2C, KIF4A, KIF5A, KLF2, KLF9, KLHL13, KLHL38, L1TD1, LAMC2, LATS2, LCA5, LDOC1, LHX6, LIX1L, LMCD1, LOXL1, LPAR1, LRFN1, LRP1B, LRRTM1, LTF, MAG, MAGEA2, MAGEB2, MAGED1, MAGI2, MAN1A1, MAP1A, MAP3K12, MAP3K3,

		MAPK8IP1, MARK1, MCF2, MFAP5, MGAT5B, MGP, MIOX, MLF1, MMP3, MPDZ, MPP7, MPPED2, MSH4, MSMO1, MST1, MT1A, MYBL1, MYEF2, MYOZ1, MYOZ2, NACAD, NAP1L3, NAV2, NCS1, NDNF, NEFM, NELL1, NES, NEXN, NOD1, NOS1, NOS3, NPAS4, NPM2, NPR1, NR2F6, NRCAM, NTRK2, NUSAP1, NXNL2, OAS1, OASL, OLR1, ORC1, OSR1, P2RX7, PARP14, PAX9, PBXIP1, PCDH15, PCDHGA7, PCSK1N, PCSK4, PCSK9, PCYT1B, PDE4B, PDGFRB, PEG10, PHLDB3, PIF1, PIP4K2A, PKN1, PLCD4, PLCG2, PLEKHB1, PLK4, PLOD2, PLVAP, PLXDC2, PM20D1, PNPLA3, POTEF, PPP1R12B, PRICKLE1, PRNP, PTBP1, PTCH2, PTGER3, PTH1R, PTHLH, RAB32, RAB3C, RAB9B, RASL11B, RBFOX1, RBM45, RBMS1, RCAN2, RECK, RFPL4B, RGN, RGS9, RHBDL2, RHBDL3, RIN3, RLBP1, RND1, ROBO4, RPH3AL, RRAGB, RTBDN, RYR2, S100A16, S100A2, SACS, SAFB2, SCN1A, SCN5A, SCUBE3, SEMG2, SEPT6, SESTD1, SFRP1, SH3D21, SH3GL2, SHANK1, SHANK3, SIM2, SIRT4, SLC12A4, SLC27A2, SLC6A2, SLPI, SMO, SNAP91, SOCS2, SORBS1, SOX10, SOX15, SPATA7, SPRY1, SRMS, SRSF2, STARD13, SYCE1, SYT1, TAGLN, TBKBP1, TBX20, TCEAL2, TCTN1, TEAD1, TEC, TEF, TET1, TGFA, TGM2, THSD7A, TIMP2, TIMP3, TIMP4, TM7SF2, TMCC2, TMEM57, TNNT3, TPST1, TRIM25, TRPC4, TSHZ3, TTC9B, TUSC3, TWIST2, TXLNB, UCHL1, UHMK1, UTF1, VAMP1, WNT2, WWTR1, XRCC4, XYLT1, YPEL5, ZDHHC1, ZEB1, ZEB2, ZFP37, ZHX2, ZIC2, ZNF14, ZNF221, ZNF256, ZNF331, ZNF442, ZNF462, ZNF470, ZNF471, ZNF549, ZNF568, ZNF583, ZNF606, ZNF649, ZNF655, ZNF662, ZNF665, ZNF667, ZNF676, ZNF677, ZNF793, ZSCAN1
	red	HSPA8, UBQLNL, HIST1H2BN, HIST1H2BO, MGA, ALDOC, ARAF, ASB16, ASS1, CASP4, CBX2, CBX5, HIST2H2AC, HK2, HLTF, IL32, PPP1R9A, PTCH1, RAC3, RS1, TCEB3B, TMEM132A, TNNI3, TRIM55, WBP2NL, ZC3HAV1, ZNF257, ZNF658
	brown	FYN, HIST1H1D, HOXB5, SH3KBP1, AKR1B1, BCL11B, CARD11, COBL, DISC1, KCNAB2, LYL1, MGAT3, NFATC1, NPHS1, PACSIN3, PPP3CA, SYVN1, TNFRSF4, VCAM1
UCEC	blue	HSPA1L, TUBA1A, ACTA1, HIST1H3A, MET, NTN1, TMEM17, KRT40, USHBP1, AIFM1, ESR2, HIPK2, HIST1H2BH, KRT9, PARK2, TFCP2, ALDH1A2, ESR1, FASLG, HSPB1, NR4A1, PTCH2, ARRB1, ATP2B2, EPB41L5, FN1, GNAI1, HS3ST6, ISL1, KCTD17, MAP1LC3A, NPHP1, OPTN, PCYT1B, PLCG2, PLG, PNCK, POU5F1, SH3GL3, SPERT, TEC, TREH, ADRB2, ALDH3B2, ASCL2, ATP1A3, ATP2B3, B3GNT7, BIRC3, CCDC87, CD1B, CEACAM21, CHP2, CHST9, CNTN1, CUL4B, DMKN, DNAJB13, DTX1, FATE1, FBXO15, FCGR3B, FGF1, FOXD4, FOXP3, GAL, GK, GPT, GRIA2, GSTA4, IDH3G, ITK, JAK1, KCND2, KPNA5, MMRN1, NRP1, OXCT1, PEG10, PLS3, PRMT8, PRPS1, RGMA, RIBC2, SLC36A1, SULT1C2, TGFBR3, TPPP, TUBB2A, UBASH3B, ABCC6, ABLIM2, ACSL1, ADAM8, ADH6, AGR3, AKAP5, AKR1C4, ALDH3A1, ALDH3B1, ALPP, ANXA13, ANXA4, ATP4A, ATP4B, B9D2, BCAR3, BCL2L11, BMP2, BMPR1B, C2orf50, C6orf165, CCDC146, CCDC39, CCL25, CCR9, CD109, CD19, CDKN2A, CHST4, CLDN10, CLDN18, COL4A5, COL8A1, CREB5, CRYAB, CRYZ, CST6, CTSD, CWH43, CXCL1, CYP27B1, CYP2R1, CYP2S1, CYP4B1, DHRS9, DPP10, DSC2, DST, DYDC1, DZIP3, EBF2, EGLN3, ERBB4, F13A1, F2RL2, FCN1, FGFR4, FNBP1, FOXB1, FOXC2, FST, FXYD3, GABBR2, GALR2, GLRA2, GNB3, GPR18, GPR50, GRM1, GSTA3, GZMB, HOOK1, HOXB5, HOXB8, HOXC10, HRG, IFNG, IL5RA, INADL, INPP5D, IQGAP2, IRF9, KATNAL2, KCNJ1, KIF17, KRT6C, KRT75, LAMA3, LAMP2, LGR5, LMO1, LMX1A, LRIG1, LRRC18, LYZ, MAP3K15, MAPK10, MAPRE3, MEIS1, MEP1A, MIA, MID1IP1,

		MOGAT1, MTMR8, MYB, NAPSA, NBEAL1, NDUFA4L2, NEK10, P4HA3, PGR, PIK3CG, PLCB2, PRAM1, PRKX, RAB3B, RAB41, RAPGEF3, RASSF9, RBM24, RBM43, RBMX2, RDH12, RHEBL1, ROBO2, RRAGD, SCD5, SCIN, SCN1A, SETD7, SHD, SLA2, SLC27A6, SLC43A3, SLC6A17, SLC9A2, SLC9A3R1, SLC9A4, SLIRP, SMAP2, SP100, STK32C, STOM, STRN, SULF1, SYNGR1, SYP, TAF9B, TFAP2C, TFCP2L1, THRSP, TLX2, TMEM27, TMOD2, TNFSF13B, TP73, TTLL6, TXK, TXLNB, UBE2L6, UGT2B15, USH1C, USH1G, USP2, VEGFA
	brown	HNF4A, GABARAPL1, CYP8B1, JUN, SOX2, CALML5, CYP4F22, MYH14, ALX4, CYP17A1, CYP4X1, GJA1, LEFTY2, PRKCA, SMAD3, STXBP5L, CYP27C1, CYP2C18, CYP3A5, EIF5A2, FOXA1, FYN, GADD45A, GFPT2, PPARD, ACSL4, ACSS3, CAMK1G, CPA2, CYP24A1, CYP2C9, DLG2, FOXQ1, GFPT1, GSTM3, KRT5, LEFTY1, LIMCH1, MARCKS, MGAT5B, MMP7, NT5E, NTRK2, PDHA1, PPP2R2C, PRDX1, S100A2, SULT4A1, TMOD1, TUBA3E, ADAMTSL2, ADAP1, ALPI, APOH, AREG, ARSJ, BDNF, BMP4, CALML3, CAP2, CCL2, CD36, CDC14A, CDH2, CDSN, CHST11, CLDN2, COL18A1, COL4A2, COL4A3, CORO6, CPE, CSRP2, DACH1, DLX1, DPP4, ELN, EN2, FAIM2, FAM83A, FEZF1, FHL2, FRAS1, FSTL4, FZD7, GABRR1, GAL3ST2, GLIS3, GNAO1, GNMT, GPC6, GSTT2, HAND1, HOXD8, HSPA12A, HTR7, IFITM3, IGSF21, ITLN2, ITPR2, KLK3, KRT27, KRT31, LGR4, LMO2, MGST1, MOXD1, MPO, MPP1, MTTP, MYH7B, NECAB2, NMNAT3, NRCAM, OBP2A, OTX1, OTX2, PKP4, PLS1, POU3F2, PPARGC1B, PRKCQ, PRKD1, PRSS3, PTPRE, QPCT, RBP1, RPL5, SATB2, SERPINB13, SERPINI1, SH3KBP1, SHANK1, SLC35A3, SLC7A2, SOX6, STXBP1, TAC1, TDGF1, TFF1, TFPI, TLE3, TNFRSF11B, TNFRSF19, TNFSF11, TRIM29, TUBB2B, ZNF860
	turquoise	KCNMA1, PGAM2, H3F3C, AKR1C2, ALB, ALDH2, GCK, H2BFWT, MYO1F, PPIA, ATP6V0A4, BCAT1, CEP250, CHRNA4, EPAS1, GAPDHS, HMGA2, LRRK2, LYN, NAP1L2, NOS3, PAX2, PFN2, PGM5, PPP2R2B, PYGM, SLC2A4, APBB1, APOA1, BATF3, CALML6, CCDC8, CORO1A, CYFIP2, DNM1, GRIN2C, GYS2, KRT13, KRT14, LYPD6, MAG, MAGEA11, MAGED1, MATR3, MYH7, NOVA1, PLK2, RAB39B, SGPP1, SLC6A8, SNAI1, ADAMTSL4, ANKRD44, ARNT2, ATP6V1C2, BEX2, COL1A1, CYP26C1, CYP4F12, CYTH4, DNMT3L, EEA1, ENO3, EPHB1, ESRRB, GDF9, GLRX, LGALS3, MAGED2, MAGI2, MAPK13, MAPT, MIPOL1, MYH15, MYOCD, MYOD1, NEDD9, NONO, NTN3, PABPC5, PGA5, PITX1, PKP2, PPP2R5B, PRKAR2B, RAP1GAP, RBM4B, SH3GL2, SOD2, SPOCK2, USP51, UTP20, VDR, WASF1, WASF3, AJUBA, AKAP3, ALS2CR11, AMY2B, AP1S2, ATF5, ATP2B1, BACH2, BAI1, BARX2, BATF, BATF2, BCL2A1, BCORL1, BMF, BRCC3, CACNA1B, CACNB4, CD2AP, CDK6, CHRNB2, CHRNB4, COL3A1, COL9A1, COMP, CPS1, CPVL, CRMP1, CXXC5, CYP26B1, DBI, DLK1, DLX4, DOCK3, DOK1, ECEL1, EFEMP2, EFNB3, EFS, ELAVL3, ELF5, ELOVL4, ELOVL5, ENOX1, EPHA2, ERLEC1, EVC2, FAM46C, FAM71E2, FAP, FERMT3, FGF9, FGFR3, FKBP1B, GABRA3, GABRE, GAL3ST4, GNAZ, GNGT2, GPR20, GRB14, GRIN2A, GRIN2D, GRK5, GYG2, HAL, HCK, HEATR5B, HEY1, HNRNPH3, HOGA1, HOXC4, HTATSF1, IL24, INA, IRF4, JAM2, KCNAB1, KCND1, KCNIP4, KCNJ4, KCTD12, KLF12, KLK6, KLK8, KRT19, LOXL1, LOXL4, LRRC26, LRRC7, LYNX1, MAGEA4, MAGEA6, MAL, MCOLN3, MEST, MSLN, MYH6, NCAM1, NCKAP1L, NDST3, NEFH, NFIL3, NLRP1, NPAS2, NPY, NR5A1, NXF5, NXT2, PAH, PAK3, PCK1, PFKFB1, PJA1, PLP1, PLXNB3, PPARGC1A, PPP1R18, PRKCG, PRSS1, PRSS21, PSMB9, PSMD10, PSTPIP1, PTPN5, RAB33A, RAB9B,

		RADIL, RANBP2, RASAL1, RASL10B, RD3, RETSAT, ROBO3, RRAGB, RTN4R, S1PR5, SALL2, SAT1, SERPINB5, SERPINC1, SFRP1, SLC25A41, SLC2A6, SLC38A9, SLC4A7, SLC6A12, SLC6A20, SLIT2, SNAI2, SNCG, SNX7, SPOCK3, STON2, SYN1, TBKBP1, TEX15, TNFRSF1B, TNFRSF25, TNFSF12, TNFSF15, TREML2, TRIM10, TRIM43, TRIM54, TSC22D3, UACA, UCHL1, UGT2B7, USP6, UTS2, UTS2R, VMA21, WAS, WNT8B, WT1, ZBTB33, ZC3HAV1, ZNF154, ZNF175, ZNF214, ZNF311, ZNF462, ZNF488, ZNF610
	green	HSD17B3, MLH1, SERPINF1, ALKBH3, CORO2B, EPM2AIP1, H2AFJ, INSR, IRAK3, MAP1B, NDN, NGFR, NTRK3, PTH1R, PTPRO, SEC14L4, SLC16A2, SNCA, TCF4
	yellow	AR, LIMA1, NOTCH2NL, RAP2C, AURKB, CCNB2, CFTR, DDX3X, HSPA8, LAMC3, NFE2, RAD51, USP9X, ABCE1, ADAM10, ASB12, BHLHE41, BRCA2, BZRAP1, C16orf59, CASP4, CCNB1, CDCA5, CREB3L1, CYP27A1, CYP2C8, DSCAML1, EZH2, FES, FOXN2, GAS2L3, GATA2, GTF2H3, GTSE1, HIST1H2BD, HJURP, HK2, HP, IGBP1, KHDRBS1, KLK2, LMNB2, PDE9A, PGAM4, PLCL2, PM20D2, PSAT1, RAB17, RPL22L1, SEC24A, SERPINA3, SERPINB6, SLC22A23, STMN1, TLX3, TSPAN10, UBQLNL, UPP1, ZSCAN1

No significant modules were found for PRAD.

Interactions were filtered using the InWeb_IM high confidence cutoff >= 0.156.

Table S3. NOA rewiring hubs*

Cancer type	Genes
BLCA	CYP11A1, ITGA2B, TUBB4A, TUBB2B, COL2A1, COCH
BRCA	PLK1, CCNB1, HSPA1A, AURKB, BUB1B
COAD	SYT1
HNSC	DAB1
KIRC	CCNB2, CYP11A1, CYP1B1, BUB1B, PLK1, CARD11, CDC20, TTK, CDK1, CYP2C18, AURKB, CYP3A7, CDCA8, BIRC5
KIRP	BRCA1, CCNB2, CCNB1, CCNA2, BUB1B, PLK1, CCNE1, CDC25C, CDK1, ESPL1, RAD51, CDC45, CDC6
LGG	SLC6A2, VDR, BUB1B, PLK1, WDHD1, FANCD2, PRC1, KCNA5, NDC80, CDC6, CYP11A1, CCNA2, LRRK2, CDT1, SMC4, AURKB, CHEK2, CDC25A, CHEK1, BRCA1, BRCA2, BUB1, SHC1, CCNB2, CDC25C, CENPE, SGOL1, MCM5, MCM4, MCM3, MCM2, CASC5, HIST1H2BO, HIST1H2BH, HIST2H2AC, CYP2C8, CCNB1, ECT2, GINS4, CDK2, RAD51, CDC45, FLNA, SCN5A
LIHC	ERBB3, TFF1
LUAD	CCNB1, BUB1B, CENPE, CDT1, MCM6, PRC1
LUSC	LIN7A, CRP, PRKAA2, RET, MAP2, HSPA1B, NAP1L2, AMBP, ATP6V1B1, GRIN1, CDH2, PGK2, NOS1, KCNJ11, H2BFWT, CYP2A7, SULT1E1, HIST1H2BM, KCNJ2, CCNB1, LMNB1, SULT4A1, ATP6V0A4
SKCM	RAC3, CYP19A1, HIST1H2BD, MYO5A, HIST1H2BO, TUBB2A
STAD	LIN7A, SFTPD, BUB1B, RET, HSPA1B, PCDHGB1, L1CAM, DRD1, SNCAIP, CACNA1A, CACNA1B, NTRK2, DLGAP1, AURKB, CDH2, ERBB4, CARD11, PPP2R2C, CHST9, OPRM1, TUBB2B, SNAP25, CHST13, KIF1A, SYT1, DRD2, CDC20, ADRB1, PFN2, SYP, OPRD1, HS6ST2, SCN5A, HIST3H2A
UCEC	EFNB3, EFNB1, EPHA4, CYP2W1, DAB1, CDC7, CYP11A1, MYOD1, CYP26B1, MAGI2, EPHB2, CDC25C, SGOL1, PRKCG, H2BFWT, SNAI1, CYP2C9, CYP1A2, CYP8B1, GRIN2C, GRIN2A, CYP2C18, CYP26C1

*NOA rewiring hubs meet the requirements:
1: Significant upregulation in EdgeR with FDR < 0.05 and Log-FC>=0.5
2: At least 3 rewiring partners with significant differential gene expression from EdgeR with FDR < 0.05

Table S4. NOA rewiring hubs associated with NOA in the literature

Gene ID	PubMed ID	Validated in the literature*	Stress type/mechanism
ADRB1	-	0	-
AMBP	-	0	-
ATP6V0A4	-	0	-
ATP6V1B1	-	0	-
**AURKB**	27702988, 20519624, 20643922, 25175806, 17589519, 24444383	2	DNA damage
BIRC5	-	0	-
BRCA1	-	1	DNA damage
BRCA2	-	1	DNA damage
BUB1	26912231	1	Chromosome instability
**BUB1B**	23242215, 21646403, 17189715	2	Aneuploidy
CACNA1A	-	0	-
CACNA1B	-	0	-
CARD11	-	0	-
**CASC5**	19490893	2	Mitotic stress
**CCNA2**	19490893	2	Mitotic stress
CCNB1	-	0	-
CCNB2	-	0	-
CCNE1	20336784	1	Chromosome instability
CDC20	26912231	1	Mitotic stress
**CDC25A**	19305144, 20228808	2	DNA damage
CDC25C	-	0	-
**CDC45**	23643534	2	DNA replication stress
**CDC6**	19490893, 22613949, 27702988	2	DNA damage
**CDC7**	23066029, 18469809	2	DNA damage
**CDCA8**	19490893	2	Mitotic stress
CDH2	-	0	-
**CDK1**	27702988, 26881434, 24444383	2	Mitotic stress
**CDK2**	27702988, 20010815, 16760657	2	Proteotoxic stress
CDT1	25115388	1	Proteotoxic stress
CENPE	23236152	1	Chromosome instability
**CHEK1**	27702988, 22446188, 22120667, 22373487, 17019715, 17255282, 19269363, 28484242, 21389083	2	DNA damage
CHEK2	19878869, 23761041	1	DNA damage
CHST13	-	0	-
CHST9	-	0	-
COCH	-	0	-
COL2A1	-	0	-
CRP	-	0	-
CYP11A1	-	0	-
CYP19A1	-	0	-

CYP1A2	-	0	-
CYP1B1	-	0	-
CYP26B1	-	0	-
CYP26C1	-	0	-
CYP2A7	-	0	-
CYP2C18	-	0	-
CYP2C8	-	0	-
CYP2C9	-	0	-
CYP2W1	-	0	-
CYP3A7	-	0	-
CYP8B1	-	0	-
DAB1	-	0	-
DLGAP1	-	0	-
DRD1	26477316	1	-
DRD2	-	0	-
**ECT2**	16862181, 28110998	2	DNA damage
EFNB1	-	0	-
EFNB3	-	0	-
EPHA4	-	0	-
EPHB2	-	0	-
ERBB3	-	0	-
ERBB4	-	0	-
ESPL1	-	0	-
**FANCD2**	24728800, 23633493, 15694335	2	DNA damage
FLNA	-	0	-
GINS4	-	0	-
GRIN1	-	0	-
GRIN2A	-	0	-
GRIN2C	-	0	-
H2BFWT	-	0	-
HIST1H2BD	-	0	-
HIST1H2BH	-	0	-
HIST1H2BM	-	0	-
HIST1H2BO	-	0	-
HIST2H2AC	-	0	-
HIST3H2A	-	0	-
HS6ST2	-	0	-
**HSPA1A**	21297664. 17332370, 25347739, 19001088	2	Proteotoxic stress
**HSPA1B**	21297664. 17332370, 25347739, 19001088	2	Proteotoxic stress
ITGA2B	-	0	-
KCNA5	-	0	-
KCNJ11	-	0	-
KCNJ2	-	0	-
KIF1A	-	0	-
L1CAM	-	0	-

LIN7A	-	0	-
**LMNB1**	19139261, 26654219, 28927262	2	Oxidative stress, Autophagy
**LRRK2**	19640926, 28768533	2	Autophagy, Proteotoxic stress
MAGI2	-	0	-
**MAP2**	15920168, 20505215, 18662975	2	Centrosome clustering, Chromosome instability
MCM2	15007098, 25628920	1	Replication stress
**MCM3**	15007098, 25628920, 25809478	2	Replication stress
MCM4	15007098, 25628920	1	Replication stress
MCM5	15007098, 25628920	1	Replication stress
MCM6	15007098, 25628920	1	Replication stress
MYO5A	-	0	-
MYOD1	-	0	-
NAP1L2	-	0	-
**NDC80**	23591767, 18922912	2	Chromosome instability, Mitotic stress
NOS1	-	0	-
NTRK2	-	0	-
OPRD1	16696856	1	Oxidative stress
OPRM1	-	0	-
PCDHGB1	-	0	-
PFN2	-	0	-
PGK2	-	0	-
**PLK1**	19490893, 26912231, 21772266, 26478211, 27702988, 19269363, 28069876, 16557283	2	Mitotic stress, DNA damage
PPP2R2C	-	0	-
**PRC1**	27429838, 28426098	2	Chromosome instability
**PRKAA2**	23644529, 21941369, 22660331, 17237771, 25373897, 25448702	2	Energy stress, Autophagy, Metabolic stress
**PRKCG**	-	0	-
RAC3	-	0	-
**RAD51**	24021650, 19942681	2	DNA damage
RET	-	0	-
SCN5A	-	0	-
SFTPD	22509983	1	Immune stress, Oxidative stress
**SGO1**	24055156, 26847209	2	Chromosome instability
**SHC1**	-	0	-
SLC6A2	-	0	-
**SMC4**	19490893	2	Mitotic stress
**SNAI1**	28176759, 28344883	2	Metabolic stress, Hypoxia
SNAP25	-	0	-
SNCAIP	-	0	-
SULT1E1	-	0	-
SULT4A1	-	0	-
SYP	-	0	-

SYT1	-	0	-
TFF1	-	0	-
**TTK**	23700430	2	Mitotic stress
**TUBB2A**	16023594, 15367667, 19269363	2	Mitotic stress
**TUBB2B**	16023594, 15367667, 19269363	2	Mitotic stress
**TUBB4A**	16023594, 15367667, 19269363	2	Mitotic stress
VDR	-	0	-
WDHD1	27940557, 27940552	1	DNA damage

* Ranking:
2: Has been experimentally studied as a NOA gene the literature
1: Has been associated to stress-related mechanism or weakly to NOA in the literature
0: Has not been linked to NOA in the literature

Table S5. Differential gene expression across cancer types

The table is available for download following the link

https://doi.org/10.6084/m9.figshare.5999441.v1

Table S6. 90% subsampling

Cancer type	Iteration	N genes old	N genes new	N genes overlap	%overlap	pvalue
BLCA	1	4952	4940	4647	88.5986654	0
BLCA	2	4952	4969	4709	90.34919417	0
BLCA	3	4952	4968	4684	89.45760122	0
BLCA	4	4952	4853	4608	88.66653839	0
BLCA	5	4952	4807	4609	89.49514563	0
BRCA	1	2077	2032	1963	91.47250699	0
BRCA	2	2077	2086	1989	91.49034039	0
BRCA	3	2077	2267	2021	86.99956952	0
BRCA	4	2077	2065	1993	92.74080968	0
BRCA	5	2077	2014	1963	92.2462406	0
CESC	1	6618	6757	6472	93.75633782	0
CESC	2	6618	6730	6478	94.29403202	0
CESC	3	6618	6521	6320	92.68221147	0
CESC	4	6618	6773	6478	93.70750759	0
CESC	5	6618	6809	6522	94.45329471	0
COAD	1	2637	3644	2395	61.63149768	0
COAD	2	2637	3814	2350	57.30309681	0
COAD	3	2637	3323	2401	67.46277044	0
COAD	4	2637	3540	2401	63.58580508	0
COAD	5	2637	3930	2455	59.70330739	0
HNSC	1	3396	3452	3233	89.4329184	0
HNSC	2	3396	3560	3268	88.61171367	0
HNSC	3	3396	3041	2883	81.11986494	0
HNSC	4	3396	3425	3216	89.20943135	0
HNSC	5	3396	3246	2994	82.07236842	0
KIRC	1	3482	3800	3140	75.80878803	0
KIRC	2	3482	3632	3074	76.08910891	0
KIRC	3	3482	3212	2870	75.05230126	0
KIRC	4	3482	3572	3069	77.01380176	0
KIRC	5	3482	3710	3101	75.80053777	0
KIRP	1	5262	5609	4527	71.35876419	0
KIRP	2	5262	5373	4419	71.09073359	0
KIRP	3	5262	5191	4254	68.62397161	0
KIRP	4	5262	4963	4214	70.10480785	0
KIRP	5	5262	4751	3982	66.02553474	0
LGG	1	5104	4766	4479	83.08291597	0
LGG	2	5104	5003	4579	82.83285094	0
LGG	3	5104	4826	4451	81.23745209	0
LGG	4	5104	4509	4342	82.37526086	0
LGG	5	5104	4657	4364	80.85973689	0
LIHC	1	3638	4048	3442	81.10273327	0
LIHC	2	3638	3467	3043	74.91383555	0
LIHC	3	3638	3525	3101	76.34170359	0

LIHC	4	3638	3654	3325	83.81648601	0
LIHC	5	3638	3438	3140	79.77642276	0
LUAD	1	2614	3079	2322	68.8816375	0
LUAD	2	2614	3535	2393	63.7113951	0
LUAD	3	2614	3315	2231	60.32990806	0
LUAD	4	2614	2867	2374	76.40811072	0
LUAD	5	2614	2697	2371	80.6462585	0
LUSC	1	4660	4606	4417	91.09094659	0
LUSC	2	4660	4651	4422	90.44794436	0
LUSC	3	4660	4596	4417	91.27918992	0
LUSC	4	4660	4575	4374	89.98148529	0
LUSC	5	4660	4539	4332	89.00760222	0
SKCM	1	5759	5913	5525	89.88124288	0
SKCM	2	5759	5890	5528	90.31204052	0
SKCM	3	5759	6159	5632	89.59592746	0
SKCM	4	5759	5958	5518	89.01435715	0
SKCM	5	5759	5972	5521	88.90499195	0
STAD	1	4432	4251	4000	85.41533205	0
STAD	2	4432	4661	4221	86.63793103	0
STAD	3	4432	4690	4232	86.54396728	0
STAD	4	4432	4518	4230	89.61864407	0
STAD	5	4432	4256	4070	88.13339108	0
UCEC	1	4312	2563	2501	57.17878372	0
UCEC	2	4312	3235	2979	65.2145359	0
UCEC	3	4312	3504	3165	68.04988175	0
UCEC	4	4312	2420	2332	53	0
UCEC	5	4312	2958	2705	59.25520263	0

N_genes_old: Combined gene set for all DiffCoEx modules in original results from the paper
N_genes_new: Combined gene set for all DiffCoEx modules in the 70% subsampling results
N_genes_overlap: Overlap between N_genes_old and N_genes_new
p-value: Hypergeometric test to find significance of the overlap

Table S6. 70% subsampling

Cancer type	Iteration	N genes old	N genes new	N genes overlap	%overlap	pvalue
BLCA	1	4952	5280	4766	87.19356019	0
BLCA	2	4952	5062	4732	89.58727755	0
BLCA	3	4952	5140	4667	86.02764977	0
BLCA	4	4952	5167	4706	86.93885091	0
BLCA	5	4952	4917	4522	84.57078736	0
BRCA	1	2077	2365	2029	84.08619975	0
BRCA	2	2077	2262	1943	81.09348915	0
BRCA	3	2077	2218	1925	81.22362869	0
BRCA	4	2077	2077	1907	84.86871384	0
BRCA	5	2077	2030	1936	89.17549516	0
CESC	1	6618	6942	6517	92.53159165	0
CESC	2	6618	7088	6601	92.90640394	0
CESC	3	6618	6998	6531	92.18066337	0
CESC	4	6618	7086	6605	93.04127342	0
CESC	5	6618	6928	6506	92.41477273	0
COAD	1	2637	4086	2342	53.45811459	0
COAD	2	2637	3481	2217	56.83158165	0
COAD	3	2637	4219	2342	51.88303057	0
COAD	4	2637	3918	2445	59.48905109	0
COAD	5	2637	2849	2123	63.12815938	0
HNSC	1	3396	3929	3183	76.84693385	0
HNSC	2	3396	3143	2856	77.54547923	0
HNSC	3	3396	3156	2885	78.67466594	0
HNSC	4	3396	3565	3145	82.41614256	0
HNSC	5	3396	3151	2831	76.18406889	0
KIRC	1	3482	3116	2509	61.35974566	0
KIRC	2	3482	2981	2496	62.91908243	0
KIRC	3	3482	3119	2464	59.56006768	0
KIRC	4	3482	3156	2691	68.17836331	0
KIRC	5	3482	2939	2560	66.3040663	0
KIRP	1	5262	5542	4311	66.39457878	0
KIRP	2	5262	5288	4188	65.82835586	0
KIRP	3	5262	5634	4211	62.99177263	0
KIRP	4	5262	5570	4421	68.95960069	0
KIRP	5	5262	5317	3996	60.70180769	0
LGG	1	5104	5038	4546	81.23659757	0
LGG	2	5104	4940	4323	75.56371264	0
LGG	3	5104	4924	4369	77.20445308	0
LGG	4	5104	4952	4509	81.28718226	0
LGG	5	5104	4849	4431	80.2426657	0
LIHC	1	3638	4108	3159	68.86854153	0
LIHC	2	3638	4762	3140	59.69581749	0
LIHC	3	3638	3663	2938	67.33898694	0

LIHC	4	3638	4694	3368	67.84850927	0
LIHC	5	3638	3796	2977	66.79380749	0
LUAD	1	2614	3315	2274	62.21614227	0
LUAD	2	2614	3057	2134	60.33361606	0
LUAD	3	2614	3692	2218	54.25636008	0
LUAD	4	2614	3464	2239	58.32247981	0
LUAD	5	2614	3466	2257	59.03740518	0
LUSC	1	4660	4669	4342	87.06637257	0
LUSC	2	4660	4643	4301	85.98560576	0
LUSC	3	4660	4886	4495	88.99227876	0
LUSC	4	4660	4803	4441	88.43090402	0
LUSC	5	4660	4916	4523	89.51118148	0
SKCM	1	5759	6001	5458	86.60742621	0
SKCM	2	5759	6147	5547	87.23069665	0
SKCM	3	5759	6184	5501	85.39273518	0
SKCM	4	5759	5975	5440	86.43152208	0
SKCM	5	5759	6280	5613	87.34827264	0
STAD	1	4432	4541	4070	83.01040179	0
STAD	2	4432	4282	3677	72.99980147	0
STAD	3	4432	4309	4028	85.46573308	0
STAD	4	4432	4553	4179	86.95380774	0
STAD	5	4432	4131	3765	78.47019592	0
UCEC	1	4312	2940	2690	58.96536607	0
UCEC	2	4312	2807	2635	58.76449599	0
UCEC	3	4312	3698	3118	63.736713	0
UCEC	4	4312	3431	2888	59.48506694	0
UCEC	5	4312	2531	2365	52.81375614	0

N_genes_old: Combined gene set for all DiffCoEx modules in original results from the paper
N_genes_new: Combined gene set for all DiffCoEx modules in the 70% subsampling results
N_genes_overlap: Overlap between N_genes_old and N_genes_new
p-value: Hypergeometric test to find significance of the overlap

Table S8. **Significant METABRIC differential coexpression modules (DiffCoEx) mapped to the protein-protein human interactome InWeb_IM**

Validation cohort	Module name	Genes in module
METABRIC discovery	pink	ACTA2, PDGFRB, TIMP2, MFGE8, FBLN2, LOX, DCN, HS3ST3A1, SPARC, PCOLCE, LAMA1, PLS3, CTSK, EFEMP2, PLAU, LAMC2, CHST3, ROR2, FGF1, TAGLN, BMP1, WNT2, MMP7, MMP2
	blue	PTGS2, TGFB3, GHR, SPRY2, SPRY1, FBLN1, CAV2, MEOX1, MEOX2, CAV1, SNCAIP, ITSN1, MSX1, TGFBR2, LAMA2, ACVRL1, NDN, SNCA, OSR1, PTGIS, LEPR, OSR2, SOCS2, HBG2, HBG1, EID1, NOV
	brown	SYTL4, AP1M1, RAPGEF4, PKP4, NOSTRIN, DFNB31, AHCYL1, BHLHE40, MAP3K3, MPP5, CSNK2A2, FADD, MYB, IGF1R, FMO1, FMO5, MUC1, PEX11B, CYP4X1, GATA3, CASP10, RAB27B, RBBP8, DBNL, CCND1, FBXL2, FBXL5, CDON, INADL, MYO5C, CD3E, KIF5C, PCM1, TRAK1, UPF2, TSHR, PLXNB1, PRLR, SCNN1D, TXNRD1, SULT1C2, CDC25B, TTC8, CCM2, CHST8, PEBP4, AKR7A2, AKR7A3, UBE2E2, GSTO2, TNK1, BTF3, VAV3, NEIL1, PDZD2, FLNB, CD19, MAP2K1, SLC4A4, PEX19, DYRK2, POLB, FBXO6, CCR7, MAP3K5, NCOA3, IFT140, CYP1B1, SKP1, GP1BA, FBXW4, SIAH2, FSCN2, TJP3, ITK, SOS1, ESR1, LMO4, IRS1, CDK6, RAD50, GSTZ1, WDR19, MAP4K1, TFF1, NOTCH1, MAP2K3, PLCG1, MAP2K4, XBP1, CEBPB, SERPINA3, FYN, PGR, AP1B1, HSPA1L, ESRRA, TMBIM6, AGTR1, RASA3, CHST10, CASP3, BBS1, BTRC, BBS5, BBS4, CASP9, NEDD4L, UPF3A, ABCC8, MAPT, MLPH, MYO7A, BCL2
	yellow	HIST1H2BM, H2AFJ
	grey60	PTK2B, PITPNM1, ASAP1
	black	RPN1, EXO1, PLK1, PKMYT1, KIF4A, IKBKE, BORA, TPX2, SHMT2, ESPL1, CENPM, CENPI, CENPF, CENPE, CENPA, IP6K2, KIFC1, NKIRAS1, KIF23, NCAPG, ACTB, TOP2A, YBX1, NFKBIB, ZWINT, FEN1, PPM1G, XPO5, CCNE1, TIMELESS, TTK, DERL3, KIF2C, CHEK1, INCENP, BUB1, CDC25C, CDC25A, SGOL1, NCAPD2, BLM, TRRAP, TK1, ZWILCH, IRF7, UBE2C, CDC20, UBE2S, CDT1, DSN1, POLE, FOXM1, KIF11, FANCD2, SKP2, GAS2L3, RACGAP1, KNTC1, POLD1, PTTG1, UHRF1, CDC45, KIF20B, KIF20A, BIRC5, PSMD2, CHAF1A, PRC1, SMARCA4, RMI2, HJURP, CRY2, AURKA, AURKB, CDCA8, ASF1B, TRAF2, MCM10, MCM6, MCM5, MCM4, MCM2, CASC5, CCNA2, TRIP13, CCNB2, CCNB1, E2F3, KHSRP, NSUN2
	green	CD38, FNBP1, CD36, IFNAR2, LAPTM5, TIGIT, CD226, CD40LG, CD300LF, CANX, PLIN1, CXCR3, TNFRSF13B, RBPJ, PSMB10, LTA, FGR, PIK3CG, ORAI3, GRAP2, PAG1, LYN, PIP4K2A, CD28, CTSL, CD22, CD27, LY9, SERPINB9, FCHSD2, LPL, FASLG, PRF1, SIGLEC10, CXCL11, SOCS1, CXCL13, MSN, LAX1, MYD88, INPP5D, CD5, SYK, FCER2, C1QC, C1QB, C1QA, TNFSF13B, CXCL9, ANGPTL5, PTPN7, LAT, CCR4, MYO5A, CD300A, CD3D, ELMO1, IL7R, RASGRP3, DOCK8, TNFAIP8L2, CCL5, DOCK2, HLA-A, RASSF2, ITGA4, GAB3, HLA-E, TNFRSF17, TRAF3, ARHGAP4, RAC2, IFNL1, AIF1, LILRB2, TNFRSF13C, RUNX3, SEMA4D, PILRA, CARD8, HLA-DQB1, PIK3AP1, TRAF3IP3, LGALS9B, CCL22, KLRC2, RASSF4, CTLA4, ORAI1, CSK, VNN2, STAG3, SLAMF7, BLK, HCK, POU2AF1, RHOG, PTPN6, CR2, IRF1, CR1, TNFRSF1B, GZMB, IRF4, VAV1, CD274, TNFAIP3, KIR2DL3, KIR2DL1, CYBA, ITGAL, PRDM1, SNX20, IL15RA, MATK, BTK, CD74, NCF1, CD70, CD72, NCF4,

		SH2D1B, CSF1R, CYTH4, PTPN22, CD4, CCR1, CD6, ARRB2, CCR5, CD2, SRGN, PSTPIP1, CD79B, KCNA3, IKZF3, CD79A, FCER1G, SPI1, NLRP1, DEF6, STAT1, CGN, GRAP, CD80, BTLA, SLA2, CD84, CD86, MAPK1, ICAM3, TNFRSF6B, PDCD1, IPCEF1, ITGAX, IKZF1, RASSF5, AIM2, SPIB, LTB, LGALS9, VCAM1, CD8A, CD8B, WAS, BANK1, IL2RA, IL2RB, TJP1, SLC17A9, CCL17, KLRD1, GJD3, IL16, RIPK2, CORO1A, PSMB9, PSMB8, HLA-DPA1, DUSP2, EBI3, SLAMF1, CD53, IDO1, CYTIP, LPXN, LILRB4, CD244, PLCG2, CD247, NLRC4, LIPE, IL18BP, FYB, HLA-DRA, UBASH3B, CASQ2, FHL1, TRAT1, SELPLG, DOK2, DOK3, IL2RG, ZAP70, FCRL3, FCRL5, KLRC1, TRAF1, KLRC3, ITGB2, PIM2, ITGB7, CLNK, CARD11, PRKCB, CD40, CYBB, TNF, PIK3CD, CYTH1, LCP2, LCP1, SLA, SELL, TCL1A, CASP5, GFI1, CASP1, GPIHBP1, IL27RA, CLEC4C, SH2D2A, LCK, REC8, PTPRC, MMP9, STK4, HLA-DMB, HAVCR2, HLA-DMA
	magenta	SNX3, AP1M2, PPP1CC, CELF1, HABP4, LY96, SERPING1, WASL, AP1S2, PECAM1, CERS2, TFAP2A, S1PR1, VPS29, DDX56, TPRN, ABCB1, HCLS1, MAGI1, CXCR4, C3, MBNL1, MAPRE1, MTA1, FBXW7, GALT, MTA3, EPB41L3, SCNN1A, PIM1, PRKCA, HLA-DRB3, CD69, PLA2G2A, RRP12, ASGR2, POMC, CHST1, CXCL12, CHST7, ZEB2, TBPL1, RPS6KA3, NCK1, CFH, CFI, MBP, VPS26B, ADRB2, SREBF1, GNG2, TLR4, SELE, C1S, C1R, WIPF1, MAPRE2, SELP, CFP
	red	PPP2R1A, SYNM, WBSCR22, EFNB3, PPP2R3B, COPS4, CCNG1, POLR3A, CLSTN1, BCR, DHX37, EIF1B, SNRPA, PPFIA1, EIF3B, ANAPC10, DISC1, PPME1, RELN, ATG9A, PTBP1, DDB1, CLUH, ULK1, MAT2A, MRPS2, THOP1, MTOR, PCNT, BRF1, COPS7B, MRPS16, AKT1, CALML3, CDC26, KIT, KLC2, PRKAR1A
METABRIC validation	blue	PTK6, AP1M2, FYN, CELF1, HABP4, FOXO1, SERPING1, MAP2K7, AP1S2, SKAP2, KCNA5, RAB8B, OPTN, TFAP2A, FAS, S1PR1, RAB3IP, CNKSR1, ADRB2, RAB11A, STAP2, ETS1, KLF2, KCNS3, C3, MBNL1, FBXW7, CBLC, CSF1R, PRKCA, CD69, IL34, PRKCZ, STAT5A, LLGL2, BMP2, SOCS3, CFI, SELE, ITGAV, PRLR, CD14, SREBF1, GNG2, TLR4, MAF, C1S, REEP6, SELP
	brown	TOMM40, PLK4, PKMYT1, KIF2C, MIS18A, TPX2, ESPL1, LIG1, BRCA1, WDR4, OIP5, BARD1, CENPF, CENPE, SPAG5, CENPA, CENPU, KIFC1, NME1, KIF23, GSK3B, CCNF, AMMECR1, NCAPG, ACTB, TOP2A, DTL, ZWINT, FEN1, TRIB3, XPO5, KHSRP, TIMELESS, TTK, CHEK1, HMMR, INCENP, GSPT1, CDC25C, CDC25A, TRRAP, NCAPD2, TK1, ZWILCH, UBE2I, SIK3, GTPBP4, PSMB3, UBE2C, TUBB, CDC20, PFN1, KNTC1, UBE2S, AIFM1, MYH10, CFL1, RFC4, DSN1, FOXM1, KIF11, FANCD2, CDC6, SKP2, GAS2L3, RACGAP1, CDT1, PSMA6, PSMA4, PTTG1, TCEB1, FANCI, RBL1, CKS2, TCEA2, UHRF1, BCAP31, METTL1, GSTM2, GSTM3, GSTM4, CDC45, KIF20B, PSMB2, CENPO, TCEB3, BIRC5, MSH6, EZH2, CHAF1A, CHAF1B, PRC1, SMARCA4, IQGAP3, YWHAZ, CCNA2, ECT2, NOP56, KIF4A, SKA3, SKA2, AURKA, CDCA8, ASF1B, MCM10, SUV39H1, MCM7, MCM6, MCM4, MCM3, MCM2, CASC5, HJURP, TRIP13, E2F7, CCNB2, CCNB1, CASP3, E2F8, SAE1, TDG, DKC1, ATF5
	greenyellow	MYH11, EPHB1, CNN1, ACTG2, EFNB3, DST, NTRK2, KRT15, KRT14, KRT6B, RELN, KRT5, NTF4, COL17A1
	lightyellow	SSR1, EEF1G, EEF1D, RPL13A, RPL10, RPL12, RPS3
	purple	IGF1R, MYB, S100A8, ESR1, VAV3, TFF1, PGR, PADI4, MAPT, S100A9, AKR7A2, AKR7A3, BIN1, GATA3, XBP1
	salmon	PPP2CA, TLE1, TCF7L1, TCF7L2, MID1
	yellow	ANGPTL4, LIPE, KCNAB1, HBG2, HBB, LPL, AKR1C2, PLIN1
	green	RPS13, MCFD2, MAD2L1, EHD1, CBLB, SMAD4, NOP2, GLRX3,

		ATP5O, TOMM20, IKBKE, BTF3L4, TANK, MICAL1, CIAPIN1, PSMA3, AURKB, NFKB2, MYD88, ADRM1, ATP5G3, RPS27, NIFK, TRAF3, IL4R, RQCD1, VPS16, SP1, XPO6, HMGA1, BLM, RPS6KA3, RECQL, POLR2B, CHFR, VASP, PTPN6, YAP1, SOCS5, RANBP6, VPS18, PSMD14, KIF5B, TACC3, H2AFX, CDK1, RUNX1, KPNA2, MBD2, LMAN1, SSB, ATP5F1
	orange	DNAJC27, ARPC1B, CACNA1E, BTRC, C1QC, C1QB, C1QA, ARPC4, MAPK1, ARRB2, MAP2K4, EFHC1, NFKBIE
	black	BCL2L1, YWHAQ, HDAC7, WWP1, EGFR, GAPDH, RARA, SMAD3, IKBKB, FBP1, UQCRH, ATP5L, TSC2, HSPA1L, ATP5C1, BRIX1, KLF11, MRE11A, BCL2L11, TFDP1, MZF1, FZD7, SLC9A3R1, SCAMP1, ATP6AP1, MAPK3, GLI3, PSMC2, NDUFS5, ATP6V0C, HSPB1, SPOP, ARPC2, PRKCD, NDUFA9, SCAND1, PSMG1, UCHL5, PTPN1, UQCRFS1, SQSTM1, E2F3, NCK1, CNN3, NME3, WNT3, CRYAB, RHBDF1, ACTR3B, WDR12, ATP6V0A1, RAD50, ABCC4, PPIE, XAB2, CALCOCO2, RBM28
	tan	PTGS2, PDGFRA, TIMP2, PDGFRB, LAMA2, SPRY2, SPRY1, HAND2, SEPT4, GRASP, FBLN2, FBLN1, CAV2, LOX, FBLN5, CAV1, DCN, APOD, TEK, SNCAIP, ITSN1, TWIST1, TWIST2, PEAR1, JAM3, MEOX1, ETS2, MEOX2, MSX1, PRELP, CDH5, SLIT2, TGFBR2, NR3C1, ELN, JAM2, NDN, OSR1, OSR2, LEPR, FGF7, CYTH3, LAMC1, FGF1, COL15A1, LRP1, EMILIN1, FEZ1, ROBO3, FEZ2, COL14A1, GULP1, PTPRB, ANGPTL1, SNCA, PTRF, NOV, MMP2
	red	CD38, TAP1, TGM2, TAP2, UBE2J1, LAT2, FNBP1, IFNAR2, TIGIT, CD226, RAPGEF1, SEMA4D, MS4A1, DOK2, TNFRSF13B, PSMB10, PIK3CD, FGR, ARHGEF1, GRAP2, RELB, BANK1, CD28, DIAPH1, CD22, CTSC, CD27, TRIM21, SERPINB9, FCHSD2, FASLG, PRF1, TNFRSF25, CXCL11, SOCS1, CXCL13, POU2AF1, LAX1, INPP5D, FCER2, SYK, IL2RG, PTK2B, CSF2, MPDZ, TNFRSF17, CXCL9, KPNA1, MYO5C, CYTIP, CD3D, CD3E, CD3G, CSF2RB, RASGRP3, AUP1, RASSF2, TNFAIP8L2, CCL5, DOCK2, DOCK1, HLA-G, ABCC8, HLA-E, IRS1, TCL1A, ARHGAP4, TSHR, SUMF2, IFNL1, RASSF5, AIF1, TNFRSF13C, IKZF1, GAB3, CARD8, HLA-DQB1, SCNN1A, TNFSF13B, CD47, RASSF4, CTLA4, PLEKHA2, TTC8, GLRB, LAT, COL4A3, BLK, HCK, ITPR1, RHOG, IPCEF1, PTPN4, CR2, IRF1, CR1, TNFRSF1B, GZMB, IRF4, VAV1, CD274, PINK1, IRF8, TNFAIP3, ELMO1, PDE3B, CYBA, ITGAL, NRROS, KIR2DL4, IL15RA, LCK, CD19, CD74, CD52, NCF1, EOMES, CD40LG, SUMF1, CD72, NCF4, SH2D1B, PAG1, SH2D1A, TMC8, DOCK8, PTPN22, CD4, CD5, CD6, CCR4, CD2, CCR7, SRGN, PSTPIP1, PIK3CG, CD79B, KCNA3, IKZF3, CD79A, PTPRCAP, SPI1, DEF6, STAT1, GRAP, BTLA, SLA2, CYTH4, ICAM3, SIT1, CST7, PDCD1, NFKBIA, LYL1, ITGA4, IL7R, RAC2, AIM2, SPIB, LTB, LTA, IL3RA, POMC, CD8A, CD8B, WAS, LYN, RAB25, IL2RA, IL2RB, ITK, RAB11FIP5, MSN, CCL19, ARPC5L, IL15, IL16, TNFRSF6B, CD247, PSMB9, HLA-DPA1, SLAMF6, SLAMF1, RIPK4, MAP4K1, CD53, KLRD1, LPXN, BIRC3, CD244, PLCG2, CORO1A, WASL, NLRC4, IL18BP, FYB, HLA-DRA, UBASH3B, TRIM8, TRAT1, SELPLG, CXCR3, DOK3, ZAP70, HLA-DRB3, HCLS1, BCKDHB, FCRL3, PRDM1, KLRC1, TRAF1, KCNJ11, PIM2, CD48, ITGB7, CLNK, CARD11, PRKCB, CD40, LY9, TNF, CYTH1, LCP2, LCP1, SLA, SELL, SLAMF7, MATK, GFI1, CASP1, BBS1, IL27RA, MAP3K14, ACTR2, NEDD4L, PTPRC, GNA13, BCKDHA, STK4, WIPF1, MLPH, HLA-DMB, HLA-DQA1, HLA-DMA

Interactions were filtered using the InWeb_IM high confidence cutoff >= 0.156.
